# Random Access Memory (RAM) Contacts Waste Catalyzes Organic Reactions

**DOI:** 10.1002/gch2.202500069

**Published:** 2025-05-08

**Authors:** Daniel Pérez de los Cobos‐Pérez, Marta Mon, Antonio Leyva‐Pérez

**Affiliations:** ^1^ Instituto de Tecnología Química (UPV−CSIC) Universitat Politècnica de València−Agencia Estatal Consejo Superior de Investigaciones Científicas Avda. de los Naranjos s/n València 46022 Spain

**Keywords:** catalysis, e‐waste, gold, one‐pot reactions, RAM contacts

## Abstract

The direct utilization of metals from electronic waste (e‐waste) in catalysis is a barely explored concept that, however, should be feasible for reactions where the catalytically active species can be formed in situ from the e‐waste metal pieces. This approach circumvents any capture or isolation of particular metals, thus saving additional treatments (extractions, neutralization, separations, washings, …) and valorizing the e‐waste in its own. Here, it is shown that a metallic contact (≈1 mg) of a computer´s random‐access memory (RAM) catalyzes a variety of organic reactions in high yields. For instance, one RAM contact catalyzes the one‐pot esterification‐hydration reaction between acyl chlorides, propargyl alcohols, and water, at room temperature in 93–99% yields with turnover frequencies >0.5 million per hour. In this way, >50 kg of organic products could be prepared with just the RAM contacts discarded per year in the Institute´s recycling bin. These results open the way to directly use e‐waste in catalysis for organic synthesis.

## Graphical abstract

Wasted RAM contacts catalyze different organic reactions in good to excellent yields, with turnover numbers >0.5 million in some cases. The RAM contacts are used without any previous treatment and are recovered and reused. Kilograms of organic products could be produced with the RAM contacts of just five discarded laptops. These results open the way to use directly e‐waste in catalysis for organic synthesis.

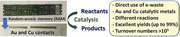

## 1 Introduction

Around 70 million tonnes of e‐waste are expected to be generated during 2024, to engross the nearly 400 million tonnes accumulated worldwide,^[^ ^1^ ^]^ with their associated harmfulness.^[^ ^2^ ^]^ Valuable metals constitute a significant part of this e‐waste, indeed, these metals can be found in higher concentration than in the corresponding ores in some cases. Unfortunately, just 1 wt.% of the e‐waste metals, which include precious metals such as Au, Pd, Pt, and others, is estimated to be recovered and recycled. The e‐waste metal recycling problem is so relevant that artificial intelligence approaches are being adopted.^[^ ^3^ ^]^ Despite new metal separation methods are continuously discovered,^[^ ^4^ ^]^ the tedious and costly processes required to extract and separate the individual metals in the e‐waste are in part behind this lack of recyclability since, in general, the market demands purified metals for most applications.^[^ ^5^ ^]^ For instance, researchers at the University of Saskatchewan have developed a very fast method to extract gold from e‐waste sources based on the simultaneous leaching and solvent extraction of gold, and also on gold leaching in acidified water‐miscible organic solvents.^[^ ^5c^ ^]^ However, a high purity of the metal is not required for other applications, which include catalytic processes, provided that metal impurities do not affect the reaction.^[^ ^6^ ^]^

**Figure 1:**
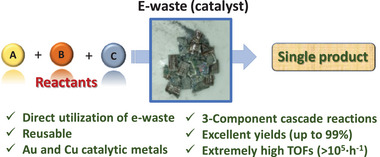
Direct use of e‐waste as a catalyst for a relatively complex organic reaction, with the associated advantages. The photograph shows real e‐waste taken from our Institute´s recycling bin.

Catalysis by metals extracted from e‐waste components is a nascent research topic with high potential.^[^ ^7^ ^]^ By definition, a catalytic process requires a small amount of metal (catalyst) to perform the reaction, thus the metal amounts that provide the e‐waste pool could be more than enough to run a chemical process even at high scales. The need of previous steps to isolate the metals in the e‐waste^[^ ^8^ ^]^ discourages researchers of routinely perform the catalytic reactions with recovered metals in the laboratory,^[^ ^9^ ^]^ not to mention in industry.^[^ ^10^ ^]^ Only few cases report the extraction and separation of metals by trapping within a solid support, and the use of this supported metal material as a catalyst of the reaction.^[^ ^11^ ^]^ Thus, any strategy towards the direct utilization of e‐waste metals in catalysis, without any previous treatment, would constitute a step forward in the valorization of the e‐waste, as shown in **Figure** **1**.^[^ ^12^ ^]^

A variety of organic reactions are catalyzed by metal species formed in situ from different metal precursors.^[^ ^13^ ^]^ Our group has looked intensively for these processes, to achieve extremely high catalytic activities after speciation of the in situ formed active species and optimization of their formation.^[^ ^14^ ^]^ One example of this has been the Au‐catalyzed one‐pot esterification‐hydration reaction of acyl chlorides with propargyl alcohols, which is catalyzed with just parts‐per‐million (ppm) of ultrasmall Au clusters (3–5 atoms, Au_3‐5_), when the clusters are formed under the proper reaction conditions, i.e., under acid conditions.^[^ ^15^ ^]^ The starting source of Au can be a salt, a complex or even nanoparticles.^[^ ^16^ ^]^ Thus, we wondered here if the macroscopic Au present in the contacts of a random‐access memory (RAM), a typical e‐waste component, could catalyze the one‐pot organic reaction, without any previous treatment of the golden contact, just a physical separation from the memory device. If so, the concept could be expanded to other organic reactions catalyzed not only by Au but also by the Cu content in the RAM contacts.[^17^]

## 2 Results and Discussion

### 2.1 One‐Pot Esterification‐Hydration Reaction

**Table** **1** shows the catalytic results for the one‐pot reaction between 4‐chlorobutanoyl chloride **1a** and 2‐methylbut‐3‐yn‐2‐ol **2** in the presence of the RAM golden contacts, at room temperature (20 °C) and without any protecting atmosphere. The dichlorinated starting acyl halide **1a** was chosen to concomitantly study the esterification and the nucleophilic substitution reactions, thus the chemoselectivity of the process. The RAM contacts were physically separated from the memory with an electric soldering iron (Figure S1, Supporting Information), and the analysis by inductively coupled plasma atomic emission spectroscopy (ICP‐AES, Table S1, Supporting Information) of different contacts after disaggregation in *aqua regia* shows that the main constituent of the metal contact is Cu (74 wt.%), and that the Au content is very significant (3.5 wt.%, Table S2, Supporting Information).

**Table 1:** Catalytic results for the one‐pot reaction between 4‐chlorobutanoyl chloride **1a** (1 mmol), 2‐methylbut‐3‐yn‐2‐ol **2** (10 mmol, solvent), and water (1.1 mmol) with RAM golden contacts under the indicated acidic reaction conditions.

Entry	RAM contacts (number, Au mol%)	HCl (µL, mol%)	HNO_3_ (µL, mol%)	Time (h)	3a (%)^a)^
1^b)^	–	–	–	150	0
2^b)^	–	10 (10)	10 (20)	150	0
3^b)^	–	10 (10)	–	150	0
4^b)^	–	–	10 (20)	150	0
5	20 (0.3)	–	–	150	12
6	20 (0.3)	10 (10)	10 (20)	4	92
7	20 (0.3)	–	10 (20)	4	>99
8	20 (0.3)	–	5 (10)	4	>99
9	20 (0.3)	–	1 (2)	150	53
**10**	**1 (0.015)**	**–**	**1 (2)**	**150**	**93**

^a)^ GC results, mass balance is completed with unreacted **1a** and alkyne intermediate **3a´**;

^b)^ **3a´** is observed as the only product.

The results in Table 1 show that the one‐pot reaction does not proceed in the absence of the RAM contacts after prolonged times (150 h) regardless the acid added to the medium (concentrated HCl or HNO_3_, entries 1–4), and that the only product observed is the alkyne intermediate **3a´** in variable amounts (10–30%), formed after the esterification reaction of **1a** with **2**. In contrast, the one‐pot reaction slightly proceeds in the presence of 20 RAM contacts (≈20 mg, containing a 0.3 mol% of Au respect to the limiting reactant **1a**) without any acid added, to give product **3a** (12%, entry 5). The alkyne intermediate **3a´** is not observed, which indicates that the metal catalyst triggers the hydration reaction. Remarkably, the yield of **3a** boosts to 92%, in just 4 h reaction time, when 10 µl of conc. HCl and HNO_3_ (10 and 20 mol%, respectively) are added to the reaction medium (entry 6), and to 99% if HCl is not added and the amount of HNO_3_ is decreased to 5 µl (entries 7 and 8). It is noteworthy to mention here that the formation of intermediate **3a´** generates 1 equivalent of HCl (100 mol%), thus the necessary acid mixture (aqua regia) for disaggregation of the RAM contact is present in the reaction medium without the need of adding external HCl. Indeed, the RAM contact‐catalyzed one‐pot reaction proceeds after adding just 1 µl of conc. HNO_3_ (2 mol%), not only with 20 contacts (53% yield) but also with just 1 RAM contact (93% yield), after prolonged reaction time (150 h, compare entries 9 and 10). It is noteworthy to remark here that the catalytic process does not use *aqua regia* anymore to extract the gold but just tiny amounts of HNO_3_. Indeed, the process is basically extracting the gold in an organic solvent, in accordance with the discovery of “organic aqua regia,”^[^ ^18a^ ^]^ a mixture of thionyl chloride (SOCl_2_) with organic solvents/reagents (i.e., pyridine, *N*,*N*–dimethylformamide, and imidazole) that can dissolve different noble metals. This last result, perhaps counterintuitive, is explained by the easier leaching of Au species at higher HNO_3_/metal ratio,^[^ ^18b^ ^]^ and gives a remarkable turnover number (TON) = 6650 (considering than one RAM contact provides 0.015 mol% of Au respect to **1a**). Control experiments with the isolated metal salts majorly present in the RAM contacts, i.e., Cu(NO_3_)_3_, Ni(NO_3_)_3_, and HAuCl_4_, and also with conc. HNO_3_, confirms that Au is the metal responsible for the catalysis (Table S3, Supporting Information). It must be remarked here that our aim is not recovering any Au from the RAM contact but demonstrating its direct use in catalysis, where minor impurities of other metals can poison the catalytic process.

**Figure 2:**
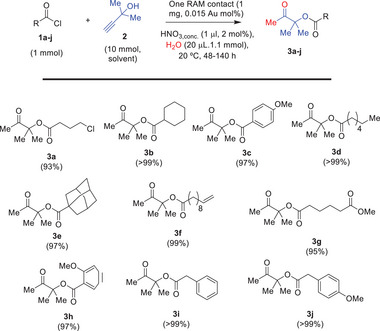
Scope for the RAM contact‐catalyzed one‐pot esterification‐hydration reaction of acyl chlorides **1a–j** with propargyl alcohol **2** and H_2_O, at room temperature (20 °C), under the indicated reaction conditions. Isolated yields.

The alkyl chloride moiety in **1a** was tolerated in all cases under the reaction conditions employed above. Indeed, **Figure** **2** (see Table S4, Supporting Information for details and Figure S2, Supporting Information for individual kinetic profiles) shows that one RAM contact is enough to catalyze the one‐pot esterification‐hydration reaction in excellent yields (93 to >99%) for a variety of acyl chlorides, including alkyl halide (**3a**), cycloalkane (**3b**), anisole (**3c**), alkyl and polycyclic chain (**3d,e**), alkene (**3f**), methyl ester (**3g**), other methoxy aromatic substituent (**3h**) and benzylic groups (**3i,j**).

**Figure 3:**
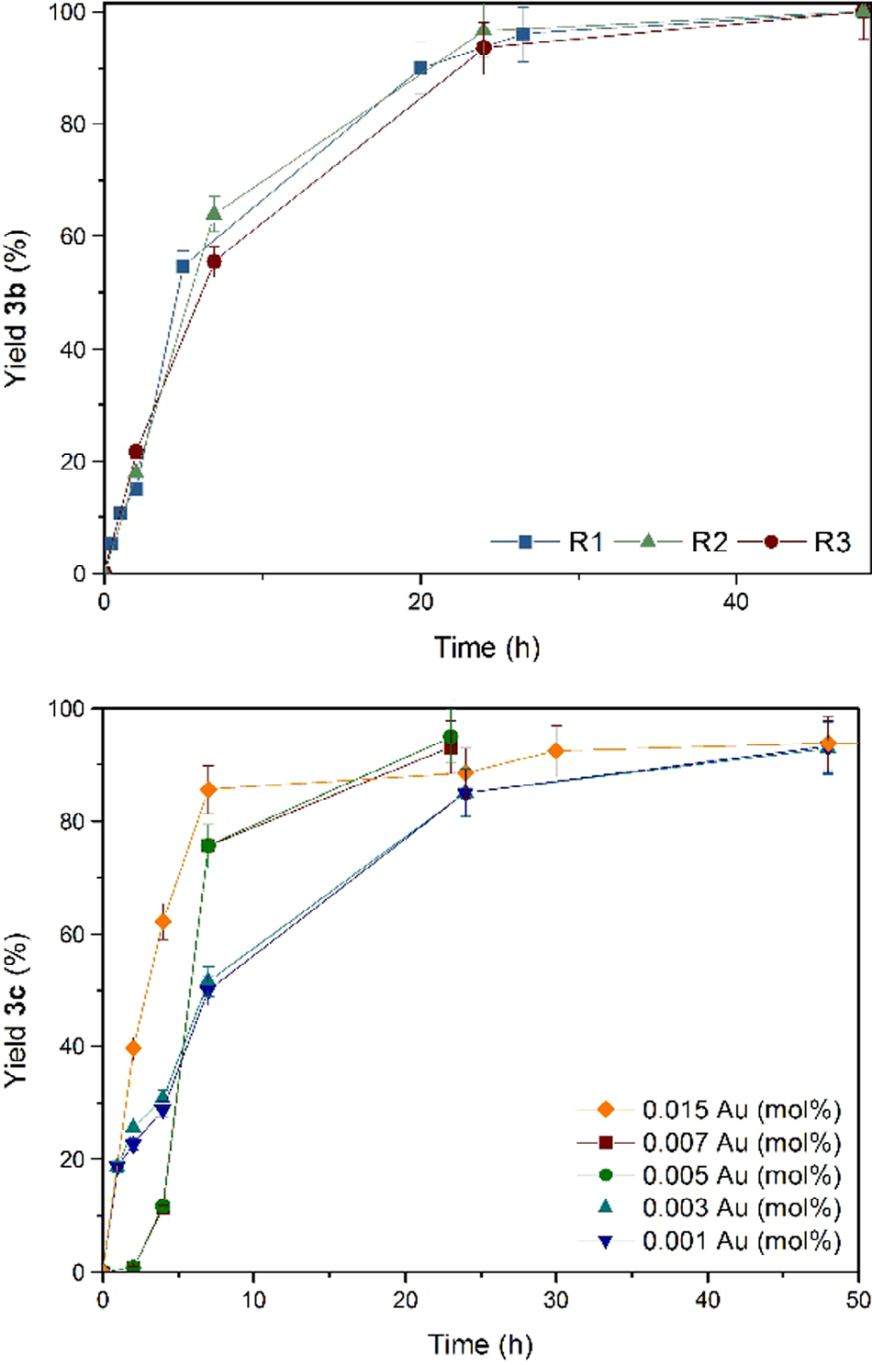
Top: Kinetic plots for the one‐pot esterification‐hydration reaction of cyclohexanecarbonyl chloride **1b** with **2** and H_2_O, catalyzed by three different contacts of a same RAM memory (R refers to the number of RAM contact). Bottom: Kinetic plots for the one‐pot esterification‐hydration reaction of 4‐methoxybenzoyl chloride **1c** with **2** and H_2_O, catalyzed by different relative amounts of RAM contact and, thus, of Au catalyst (the amount of reactants were increased respect to one RAM contact). All reactions were performed at room temperature (20 °C) and under the optimized reaction conditions in Figure 2 above. GC yields. Error bars account for a 5% uncertainty.

**Figure** **3** (top) shows the yield versus time plot for the one‐pot esterification‐hydration reaction of cyclohexanecarbonyl chloride **1b** and **2** catalyzed by three different RAM contacts extracted from a same memory, and it can be seen that the kinetic profile is very similar for the three of them. The same results were obtained for but‐3‐enoyl chloride **1f** (Figure S3, Supporting Information). Conversely, contacts from different RAM memories catalyze similarly the reaction of **1b**, with the same kinetic profile (Figure S4, Supporting Information). These results strongly support the consistency of RAM contacts to catalyze the one‐pot esterification‐hydration reaction of acyl chlorides with **2** and water.

**Figure 4:**
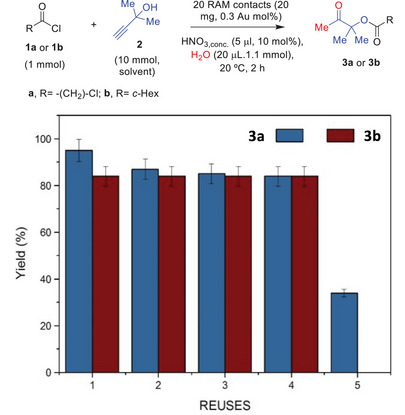
Reuses of 20 RAM contacts during the one‐pot esterification‐hydration reaction of acyl chlorides **1a** or **1b** with **2** and H_2_O, at room temperature (20 °C), under the indicated reaction conditions. GC yields. Error bars account for a 5% uncertainty.

The RAM contact was, visually, not much deteriorated after reaction. A filtration test with acyl chloride **1b** as the starting material (Figure S5, Supporting Information) shows that the catalytic activity corresponding to the Au leached into solution accounts for a 42%, while the solid RAM catalyzes the reaction in the resting 58% of the total activity. This result suggests that the solid RAM contacts catalyze the reaction in high extent and, thus, they could be reused after reaction. **Figure** **4** shows the results for the one‐pot reaction with 20 RAM contacts (0.3 Au mol%), tested with 10 mol% of HNO_3_ (since this amount of HNO_3_ is optimal for 20 contacts, see entry 8 in Table 1) and for two different substrates (**1a** and **1b**). The reuse tests were stopped before total conversion, at intermediate reaction times (2 h), to better assess any potential decrease in the catalytic activity of the RAM contacts. After one use, the contacts were simply cleaned with dichloromethane and dried with dry air, to be used again.

The results in Figure 4 show that the RAM memories can be reused four times without any decrease in the final yield of the β‐ketoester product, either **3a** or **3b**, in accordance with the partial leaching results. However, also in accordance with this partial leaching, the weight of the recovered contacts after use shows losses throughout the uses, as the contacts get partially dissolved under reaction conditions (Table S5, Supporting Information). This occurs for both substrates, and that is the reason why the yield of product **3a** decreases to more than a half after the fifth use, and why the fifth use could not be carried out for **1b**.

**Figure 5:**
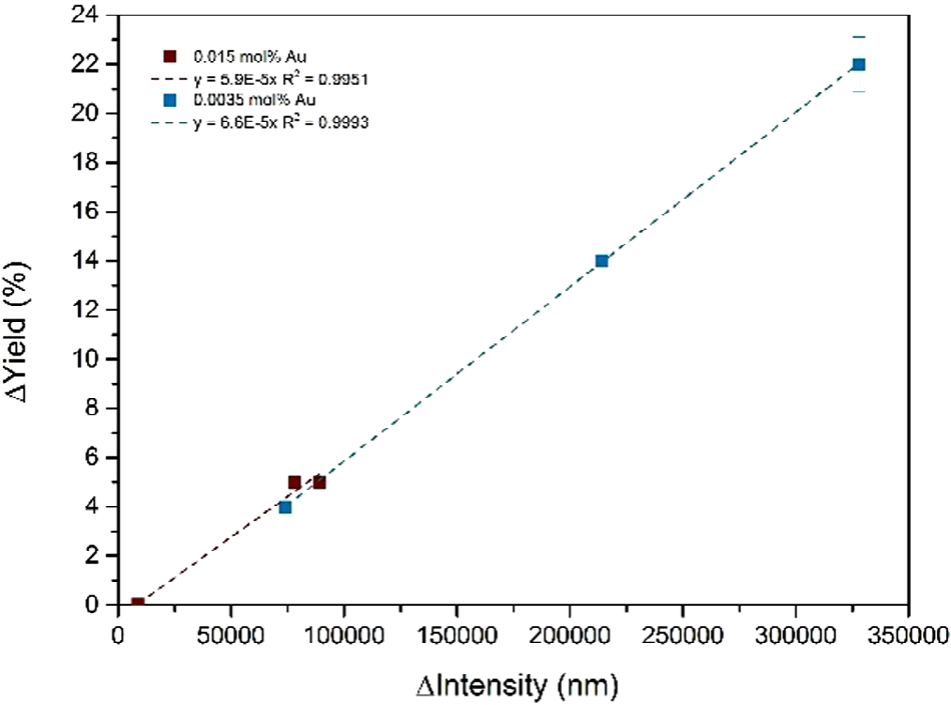
Linear variation in the yield during the one‐pot esterification‐hydration reaction of 4‐methoxybenzoyl chloride **1c** with propargyl alcohol **2** and H_2_O, catalyzed by one RAM contact at room temperature (20 °C) and under the optimized reaction conditions, with the variation in fluorescence intensity.

The fact that the RAM contacts act as a mixed heterogeneous catalyst and reservoir for Au during the one‐pot reaction is an advantage if one considers that this reaction is reported to be catalyzed by ultrasmall Au clusters in solution,^[^ ^15^, ^16^ ^]^ thus the progressive leaching of tiny amounts of Au to the reaction medium might favor the in situ formation of these ultrasmall Au species. If this is so, decreasing more and more the amount of RAM memory respect to reactants may increase, until some value, the catalytic efficiency. Figure 3 (bottom) shows that the catalytic efficiency of the RAM contact does not diminish, indeed increases, when adding up to ten times more amounts of the reactants 4‐methoxybenzoyl chloride **1c** and **2**, under optimized reaction conditions (2 mol% HNO_3_ and 20 °C), and that the same final 91% yield of **3c** is obtained with either 0.015 or 0.0015 Au mol% after 48 h reaction time. Moreover, the turnover frequency reaches 4200 h^−1^ at room temperature when a 0.005 Au mol% is added through the RAM contact, since the catalytic ultrasmall Au clusters are better formed in solution, as assessed by emission ultraviolet–visible spectrophotometry measurements (fluorescence, Figure S6, Supporting Information). Indeed, **Figure** **5** shows that a linear correlation is obtained after representing the increase in fluorescence signal with the reaction yield, confirming the catalytic activity of the fluorescent clusters. These clusters are very tiny (<7 atoms) and better formed at lower Au amounts (Figure S6E, Supporting Information). Matrix‐assisted laser desorption/ionization time‐of‐flight mass spectrometry (MALDI‐TOF MS) measurements confirm the extremely low atomicity of the formed Au clusters, since the corresponding spectra at different reaction times (Figure S7, Supporting Information) show the increase in the Au atomicity with time but just until three Au atoms, coordinated to the starting material (**1c** in this case), HCl, water and the diluting solvent (acetonitrile). It must be noticed here that the Cu content in the RAM does not interfere neither in the Au cluster formation nor in the catalysis.

The catalytic behavior of the Au in the RAM contacts, i.e., the higher catalytic activity at lower amounts, is general for most substrates in Figure 2, either by showing a faster initial rate, higher final yield, or both (see comparative kinetics for compounds **1a,b**, **1e,** and **1** **h** in Figures S8–S11, Supporting Information). The benefits of using less amount of Au for the reaction, thus of RAM contacts, were confirmed by employing as a catalyst the acid solution of the disaggregated contacts (Table S6, Supporting Information). The results show that the Au dissolved from the RAM contact catalyzes the reaction as well as AuCl (compare entries 1 and 4 in Table S6, Supporting Information), and that just a 0.00015 Au mol% (1.5 ppm of Au) of the dissolved RAM contact catalyzes the formation of the β‐ketoester product **3c** in 86% yield after 48 h reaction time, which constitutes a TON = 573 300. These results confirm the advantage of using the solid RAM contact as a reservoir of Au during reaction, since the catalytic Au species are smoothly formed in solution after slow dissolution of the metals in the contact. This can be applied to other reactions where tiny amounts of Au (or Cu) catalyst are required (see ahead).^[^ ^19^ ^]^

With the above numbers in mind, one can calculate the amount of product **3a** that could be produced with the available e‐waste. For instance, a conservative estimation gives that >2400 RAM contacts (5 laptops, 2 RAMs per 8G laptops, ≈240 contacts per RAM) can be recycled in our Institute per year. Thus, using the TON numbers shown above, 58 Kg of **3b** could be produced with the RAMs recycled in our Institute in one year. This calculation showcases the huge potential of our approach to valorize e‐waste directly through catalysis, not only for this reaction but also for related ones.^[^ ^19^, ^20^ ^]^

### 2.2 Other Organic Reactions

Other Au‐catalyzed organic reactions involving alkynes were evaluated, which include the intramolecular hydroamination of aromatic alkynes,^[^ ^21^ ^]^ the hydration and hydrochlorination of phenylacetylenes and alkyl alkynes,^[^ ^22^ ^]^ the aldehyde‐amine‐alkyne coupling reaction (A^3^ reaction)^[^ ^23^ ^]^ and the Meyer–Schuster rearrangement to chalcones.^[^ ^24^ ^]^

**Figure 6:**
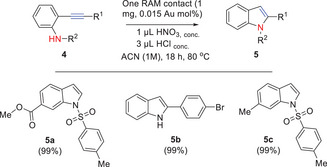
Scope for the RAM contact‐catalyzed intramolecular hydroamination reaction of aromatic alkynes **1a–c** under the indicated reaction conditions. Isolated yields. ACN: acetonitrile.

**Figure** **6** shows the results for the intramolecular hydroamination of aromatic alkynes, and the aniline derivatives **4a–c**, containing ester, sulfone, and bromide groups, cyclize in the presence of one RAM contact to generate the indoles **5a–c** after 18 h under reflux of acetonitrile, in quantitative yields (99%) in all cases (see Table S7, Supporting Information for details).

**Figure 7:**
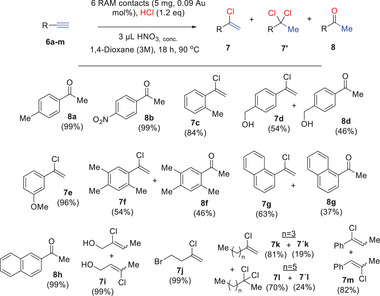
Scope for the RAM contact‐catalyzed hydration or hydrochlorination reaction of alkynes **6a–m**, under the indicated reaction conditions. GC yields.

**Figure** **7** shows the results for the hydration reaction, and the reaction of alkynes **6a–m** proceeds in very high yields when using six RAM contacts at 90 °C in 1,4‐dioxane solvent (see Table S8, Supporting Information for details). In some cases, the reaction gives the vinyl chloride products **7**, coming from the addition of HCl. For instance, the *para*‐substituted phenylacetylenes **6a,b,h** give the acetophenone products **8a,b,h** while the *metha*‐ and *ortho*‐substituted phenylacetylenes **6c,e** give the vinyl chloride products **7c,e**, all in quantitative yields. A mixture of acetophenone and vinyl chloride products is found after complete conversion of phenylacetylenes **6d,f,g**, where a hydroxyl‐directing group or different substitution positions can be found. Alkyl alkynes **6i–m** also react quantitatively in the presence of the RAM catalyst, and vinyl chlorides are exclusively found as products. These results indicate that the attack of the Cl^−^ anion as a nucleophile is favored over H_2_O for alkyl alkynes, and that the vinyl chloride products are not intermediates of the ketone products, since their hydrolysis to the latter is more favored in alkyl rather than aromatic alkynes.^[^ ^25^ ^]^ In this way, vinyl chlorides **7i–m** could be formed in very high yields without any ketone product, as vinyl chloride mixtures. For alkyne hydrocarbon chains (**6k,l**), the *gem*‐dichloride products **7k^´^,l**´ could be found in significant yields, confirming the stability of the chloride products from alkyl alkynes under the present reaction conditions. When the mixed aromatic‐alkyl alkyne **6m** was employed as a reactant, the vinyl chloride product **7** **m** was majorly found (82%). Overall, the RAM contact‐catalyzed hydration/hydrochlorination reaction of alkynes proceeds in excellent yields and tolerate a variety of functional groups (nitro, alcohol, methoxy, and bromide).

**Figure 8:**
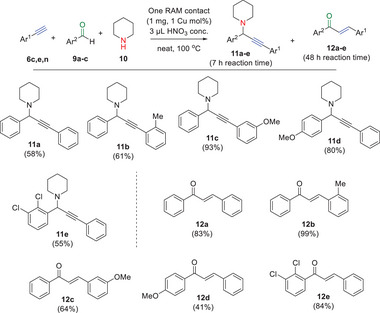
Scope for the one RAM contact‐catalyzed A^3^ coupling reaction (after 7 h reaction time) or for the Meyer–Schuster rearrangement to chalcones (after 48 h reaction time) of aromatic alkynes **6c,e,n**, aldehydes **9a–c** and piperidine **10** under the indicated reaction conditions. GC yields.

The A^3^ coupling reaction catalyzed by one RAM contact was then studied, and the results are shown in **Figure** **8**. Here, the Cu content in addition to the tiny amounts of Au present in one RAM contact was expected to also catalyze efficiently the reaction.^[^ ^26^ ^]^ Good to very good results were obtained when alkynes **6c,e,n**, aldehydes **9a–c,** and piperidine **10** were employed as reactants (other substrates were not tested), to give the corresponding propargyl amine products **11a–e** after 7 h reaction time. Kinetic experiments (Figure S12, Supporting Information) show that product **11a** smoothly evolves to the corresponding chalcone product **12a** by a Meyer–Schuster rearrangement under the acid reaction conditions. The rearrangement is catalyzed not only by the metal but also by the protons in the medium, as assessed by kinetic experiments without added HNO_3_ acid, where the amount of rearranged chlacone product **12a** is significantly diminished (Figure S13, Supporting Information). In any case, good to very good yields of chalcones **12a–e** could be obtained after elonging the reaction time to 48 h, with just one RAM contact as the catalyst. The use of Cu and Au salts, and also of the Cu extracts from the RAM contact (the Au extracts were too diluted in water) confirmed that the catalytic activity for the A^3^ coupling reaction comes from both the Cu and Au content of the RAM contact (Figure S14, Supporting Information). In this way, and considering the same amount of e‐waste just generated in our Institute in one year, >60 Kg of product **11c** could be produced.

### 2.3 Material, Economic, and Eco‐Scale Balance

**Table** **2** shows an economic and eco‐scale balance for the Au employed here during the one‐pot esterification‐hydration reaction compared to previous reports on AuCl_3_‐catalyzed reactions^[^ ^15^ ^]^ and studies on Au recovery from RAM contacts.^[^ ^27^ ^]^ The results, based on a established parametrization,^[^ ^28^ ^]^ show that our method here is not only the most economic (entries 8–10) when considering all the reagents involved, one order of magnitude cheaper than the AuCl_3_ catalysis (compare entries 8 and 9), but also as eco‐bening as other Au methods reported.^[^ ^27^ ^]^

**Table 2:** Balance of matter, and economic and eco‐scale calculations for the one‐pot esterification‐hydration reaction, compared when starting with AuCl_3_ as a catalyst^[^ ^15^ ^]^ and also with examples 9 and 10 for Au extraction in Ref. [27]

Entry	Compound	Our Work	AuCl_3_	Example 9	Example 10
1	HCl	‐	‐	0.35	0.35
2	HNO_3_	0.06	‐	‐	0.26
3	H_2_O_2_	‐	‐	0.42	0.42
4	H_2_O	1.1	1.1	2	2
5^a)^	Acyl	1	1	1	1
6	Alcohol	10	10	10	10
7^a)^	Product	1	1	1	1
**8**	***Price (€/mol)***	**158.1**	**5665.4**	**184.0**	**184.9**
**9**	***Price (€/Kg)***	**765.6**	**27 435.4**	**891.2**	**895.29**
**10**	***Eco‐scale***	**83**	**83**	**70**	**70**

^a)^ Acyl and product refers to compounds **1a** and **3a**, respectively.

The amount of acid and oxidants employed for the Au extraction/dissolution was studied in detail. **Table** **3** shows the results, and although the exact volume of extracting acid employed in some studies is not clearly indicated,^[^ ^27^ ^]^ the amount of acid/oxidant employed in general in any extracting procedure for Au seems to be one order of magnitude higher than the one here employed (compare entries 1–3). In detail, 6 grams of acids would often be necessary to dissolve 1 gram of metal, and taking into account a density of 1.3 g·mL^−1^ for *aqua regia* (average density of the mixture of concentrated HCl and HNO_3_ acids generally used), 4.6 mL of acid per gram of Au metal would be employed for extracting all the Au from the RAM contact. We indeed started from one RAM contact with total mass = 1 mg, thus our reaction requires much lower amounts of acids/oxidants and, besides, dissolves the entire metal part and not exclusively the Au. Consequently, the reported procedures^[^ ^27^ ^]^ would require 4.6 µL of acids compared to our 1.0 µL, which is a 4.5 times larger volume of acids/oxidants than during our catalytic procedure.

**Table 3:** Comparison of mol of acid/oxidants per mol of Au for the one‐pot esterification‐hydration reaction, compared with Ref. [27].

Entry	Study	Au (wt.%)	Au (mol)	Acid/oxidant (mol)	mol_Ox_/mol_Au_
1	Our work	0.0351	0.00018	0.02	112.3
2^a)^	Example 9	0.0018	0.009	21.3	2334.5
3^a)^	Example 10	0.0018	0.009	18.4	2013.8

^a)^ Ref. [27].

## 3 Conclusion

RAM contacts directly obtained from our Institute´s recycling bin catalyze a series of Au‐catalyzed and Cu‐catalyzed organic reactions in good to excellent yields (38 different products obtained here), with TONs >0.5 million in some cases. The RAM contact is directly used in the catalytic reaction, without any metal separation, capture or speciation, and it can be recovered and reused. Kilograms of organic products could be produced with the RAM contacts discarded just in our Institute in 1 year. In a simple projection, the e‐waste of some chemical companies could fulfill their own needs for particular metal‐catalyzed reactions, avoiding further extractions at origin. These results open the way to use the metals of recycled e‐waste pieces in catalysis without any previous treatment, and we hope that researchers, and perhaps industry in the future, may look into the discarded electronic pieces as the more convenient and sustainable source of catalytic metals for some reactions.

## Conflict of Interest

The authors declare no conflict of interest.

## Supporting information



Supporting Information

## Data Availability

The data that support the findings of this study are available from the corresponding author upon reasonable request.
