# Supplementary material for: Random Access Memory (RAM) Contacts Waste Catalyzes Organic Reactions
Source: Glob Chall. 2025 May 8;9(6):2500069. doi: 10.1002/gch2.202500069 (PMC12151793; doi:10.1002/gch2.202500069)
Supplement: Supplementary file 1 — Supporting Information [file GCH2-9-2500069-s001.pdf]

Global
Challenges
Open Access

Supporting Information

for *Global Challenges.*, DOI 10.1002/gch2.202500069

Random Access Memory (RAM) Contacts Waste Catalyzes Organic Reactions

Daniel Pérez de los Cobos-Pérez, Marta Mon and Antonio Leyva-Pérez**

Supporting Information
©Wiley-VCH 2019
69451 Weinheim, Germany

Random Access Memory (RAM) Contacts Waste Catalyzes Organic Reactions

Daniel Pérez de los Cobos−Pérez, Marta Mon* and Antonio Leyva−Pérez*

Abstract: The direct utilization of metals from electronic waste (e–waste) in catalysis is a barely explored concept which, however, should be feasible for reactions where the catalytically active species can be formed in situ from the e–waste metal pieces. This approach circumvents any capture or isolation of particular metals, thus saving additional treatments (extractions, neutralization, separations, washings, …) and valorizing the e–waste in its own. Here, we show that a metallic contact (≈1 mg) of a computer´s random–access memory (RAM) catalyzes a variety of organic reactions in high yields. For instance, one RAM contact catalyzes the one–pot esterification–hydration reaction between acyl chlorides, propargyl alcohols and water, at room temperature in 93–99% yields with turnover frequencies >0.5 million per hour. In this way, >50 kg of organic products could be prepared with just the RAM contacts discarded per year in our Institute´s recycling bin. These results open the way to directly use e–waste in catalysis for organic synthesis.

DOI: XXXXXXXXXX

Table of contents

Experimental section 3
Supporting Figures 5
Supporting Tables 20
Compoud characterization 28
NMR spectra 32
Author Contributions 50

Experimental section

Materials: All reagents (≥97% purity) and solvents (≥99% purity) were purchased from Sigma Aldrich-Merck and used as received unless otherwise indicated. The RAM memories were obtanied from the recycling bin at the ITQ.

Physical techniques: ^{1}H and ^{13}C nuclear magnetic resonance (NMR) spectra were recorded at room temperature on a 400 MHz spectrometer (Bruker Ascend 400) using the appropriate deuterated solvent. Gas chromatographic analyses were performed in an instrument (Shimadzu GC-2025) equipped with a 25 m capillary column of 50 %-phenyl- 50 %-dimethylpolysiloxane. *N*-dodecane was used as an external standard. GC/MS analyses were performed on a spectrometer equipped with the same column as the GC (Agilent GC 6890 N coupled with Agilent MS-5973) and operated under the same conditions. Absorption spectra were recorded on a Cary 60 UV-Vis spectrophotometer at room temperature in a wavelength range of 300-800 nm. Fluorescence spectra were obtained with a LP S-220B (Photon Technology International) equipped with 75 W Xe lamp. The photophysical measurements were performed under air at room temperature in a quartz cell of 1.0 cm optical path length. The metal content of the samples was determined after the acid treament through inductively coupled plasma-optical emission spectroscopy (ICP-OES) with spectrophotometer Varian 715 (Palo Alto, CA, EE.UU.). A 5800 Mass spectrometer with MALDI ionization source and time-of-flight analyzer [MS MALDI TOF, AMALDI TOFTOF (ABSciex)] was employed in reflector positive mode, in a range 400-1000 m/z, at 2800-3500 of laser intensity.

Separation and analysis of the RAM contacts. The golden contacts were physically separated from the RAM with an electric soldering iron. Later, the contacts (50 mg) were treated with aqua regia (6 mL) for 24 h at r.t. The solution was diluted with H_2O milliq. (24 mL) and was sent to ICP-OES for metal content analysis.

Reaction procedures

Metal (gold and copper) extraction of contacts. The golden contacts were physically separated from the RAM with an electric soldering iron. Later, treatments with HNO_3 3M (6 mL) as carried out for 20 h at 60 ^{o}C (Cu extracts). The remaining solid was treated with Aqua Regia (6 mL) for 24 h at r.t. (Au extracts). For the metal analysis, an aliquot (2 mL) of solutions (**EX-Y**) was diluted with H_2O miliq. (28 mL) and were sent to ICP-OES for analysis.

General procedure for the one-pot esterification-hydration reaction. The metal catalyst, the acyl chloride (1 mmol) and propargylic alcohol **2** (10 mmol) were placed in a 2 ml vial equipped with a magnetic stir bar. The vial was sealed, and the resulting mixture was magnetically stirred for 2 h at room temperature (20 ^{o}C). Then, water (1 mmol) was added, and the mixture was stirred for 1-5 days at room temperature (20 ^{o}C) until complete conversion of the ester. The reaction mixture was quenched with a saturated solution of Na_2CO_3 and then extracted three times with DCM. The combined organic layers were dried over anhydrous $MgSO_4$, filtered, and the solvent was removed under vacuum to give the desired compound in typically >90 % purity. Additional purification by column chromatography on silica was performed if needed.

General procedure for the synthesis of β-ketoesters with one RAM contact. One contact (≈1 mg), the acyl chloride (1 mmol), propargylic alcohol **2** (10 mmol), and HNO_3 conc. (1 μL) were placed in a 2 ml vial equipped with a magnetic stir bar. The vial was sealed, and the resulting mixture was magnetically stirred for 2 h at room temperature (20 ^{o}C). Then, water (1 mmol) was added, and the mixture was stirred for 1-5 days at room temperature (20 ^{o}C) until complete conversion of the ester. The reaction mixture was quenched with a saturated solution of Na_2CO_3 and then extracted three times with DCM. The combined organic layers were dried over anhydrous $MgSO_4$, filtered, and the solvent was removed under vacuum to give the desired compound in typically >90 % purity. Additional purification by column chromatography on silica was performed if needed.

Reusing tests. The study was carried out with 20 contacts (≈20 mg) without previous treatment and 5 μL of concentrated HNO_3. Following the general reaction procedure shown above, the contacts were cleaned with dichloromethane at room temperature and dried in air. The amounts of starting materials in the next reaction were maintained constant. After the reuse, the weight of the recovered contacts was assessed.

Leaching test. Two parallel reactions with one contact, acyl chloride **1a** (1 mmol), propargylic alcohol **2** (10 mmol), and HNO_3 conc. (1 µL) at room temperature (20 ^oC) were placed in 2 ml vials equipped with a magnetic stir bar, sealed and magnetically stirred for 2 h at room temperature (20 ^oC). Then, water (1 mmol) was added in each mixture until the conversion was 30%. Then, the contact of one of the reactions, was separated magnetically and the mixture was stirred for additional 48 h.

General procedure for the formation of indoles 5a-c (intramolecular hydromaination reaction). One contact (≈1 mg), HCl conc. (3 µL) and HNO_3 conc. (1 µL) were placed in a 2 ml vial equipped with a magnetic stir bar. After that, ACN (1 mL) and the amine (1 mmol) were added. The resulting mixture was magnetically stirred for 12 h at 80 ^oC. The reaction mixture was quenched with Na_2CO_3 solid, filtered, and the solvent was removed under vacuum to give the desired compound in typically >90 % purity. Additional purification by column chromatography on silica was performed if needed.

General procedure for the hydration and hydrochlorination reactions. Six contacts (≈5 mg), HCl conc. and HNO_3 conc. (3 µL) were placed in a 2 ml vial. Then, the corresponding alkyne (1 mmol) and hydrochloric acid 4 M in 1,4-dioxane (1.2 mmol) were added. The vial was sealed, and the resulting mixture was placed in a pre–heated oil bath at 90 ºC and magnetically stirred overnight. The solvent was removed under vacuum at room temperature and the residue was analyzed by GC and GC-MS.

General procedure for A^3 coupling and Meyer–Schuster rearrangement with acid. The aldehyde (1 mmol), piperidine (1 mmol), alkyne (1 mmol), HNO_3 conc. (3 µL) and one contact (≈1 mg) were placed in a 2 ml vial equipped with a magnetic stir bar, in the absence of solvent. The reaction mixture was warmed to 100 °C without the exclusion of air. EtOAc was added to the resulting mixture, followed by filtration through Celite and washing with additional EtOAc. The mixture was analyzed by GC and GC-MS.

General procedure for the A^3 coupling and Meyer–Schuster rearrangement without acid. The aldehyde (1 mmol), piperidine (1 mmol) and alkyne (1 mmol), and one contact (≈1 mg), were placed in a 2 ml vial equipped with a magnetic stir bar, in the absence of solvent. The reaction mixture was heated to 100 °C without the exclusion of air. EtOAc was added to the resulting mixture, followed by filtration through Celite and washing with additional EtOAc. The mixture was analyzed by GC and GC-MS.

SUPPORTING INFORMATION

Supporting Figures

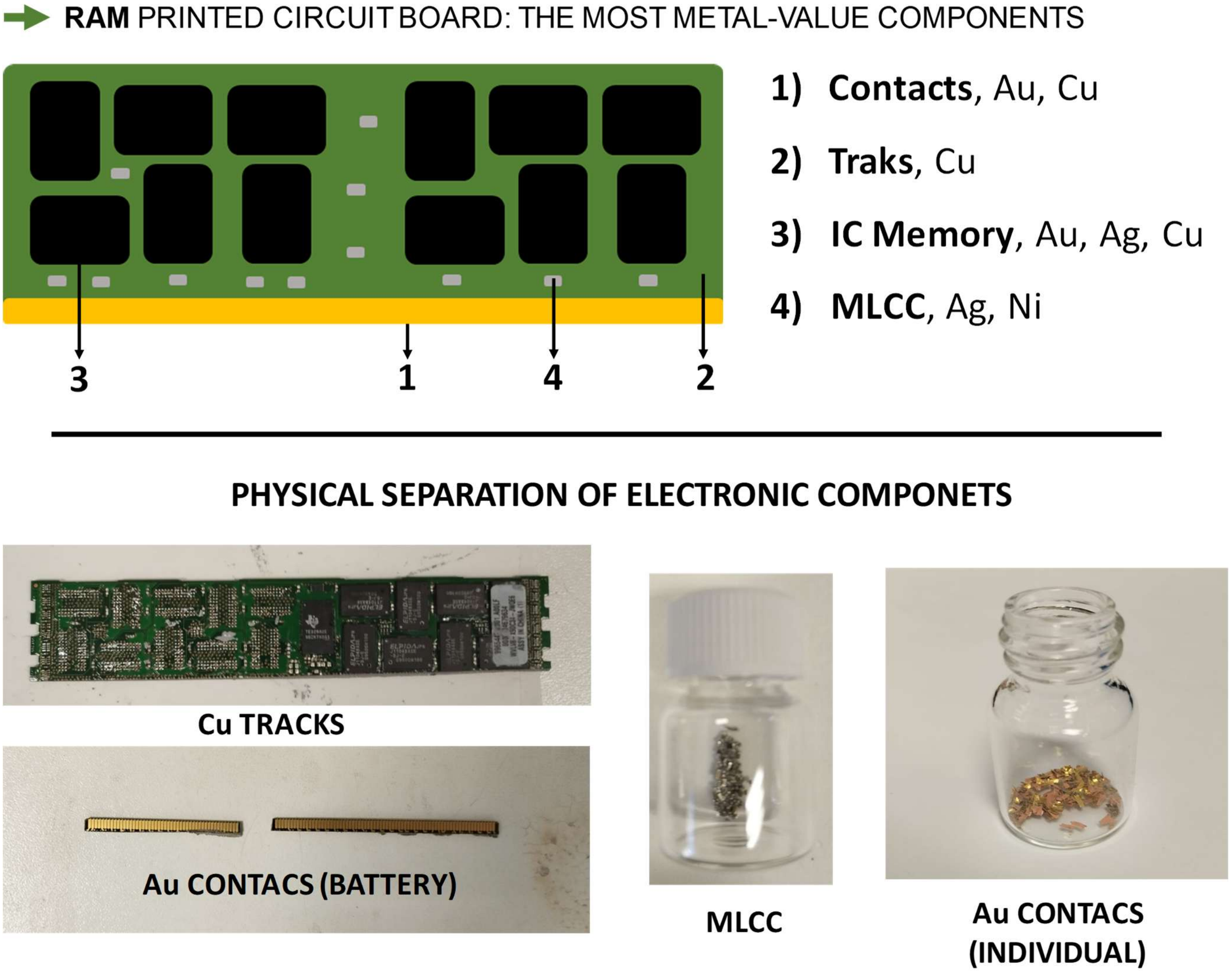

Figure S1. Top: pieces of a RAM memory (IC:..., MLCC;...), indicating the main metal components. Bottom: photographs of the physically separated pieces during our study.

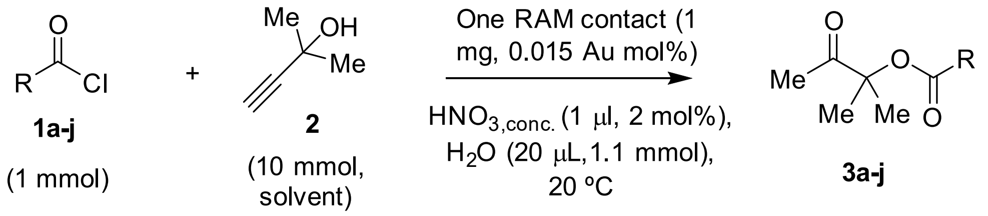
O
R
Cl
1a-j
(1 mmol)
+
Me
OH
Me
2
(10 mmol, solvent)
One RAM contact (1 mg, 0.015 Au mol%)
HNO3,conc. (1 µl, 2 mol%), H2O (20 µL,1.1 mmol), 20 ºC
O
Me
O
R
Me Me O
3a-j

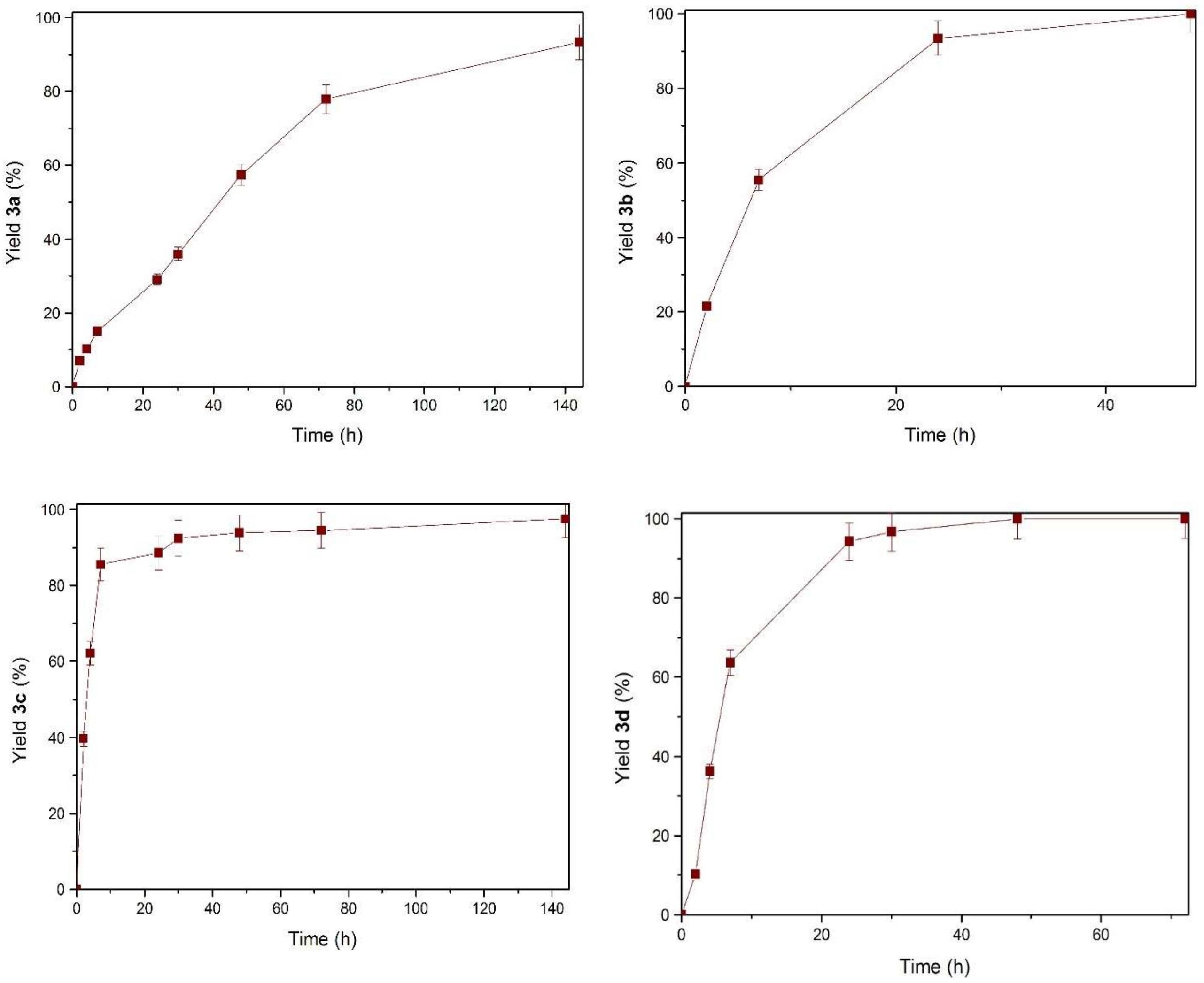
Yield 3a (%)
Time (h)
Yield 3b (%)
Time (h)
Yield 3c (%)
Time (h)
Yield 3d (%)
Time (h)

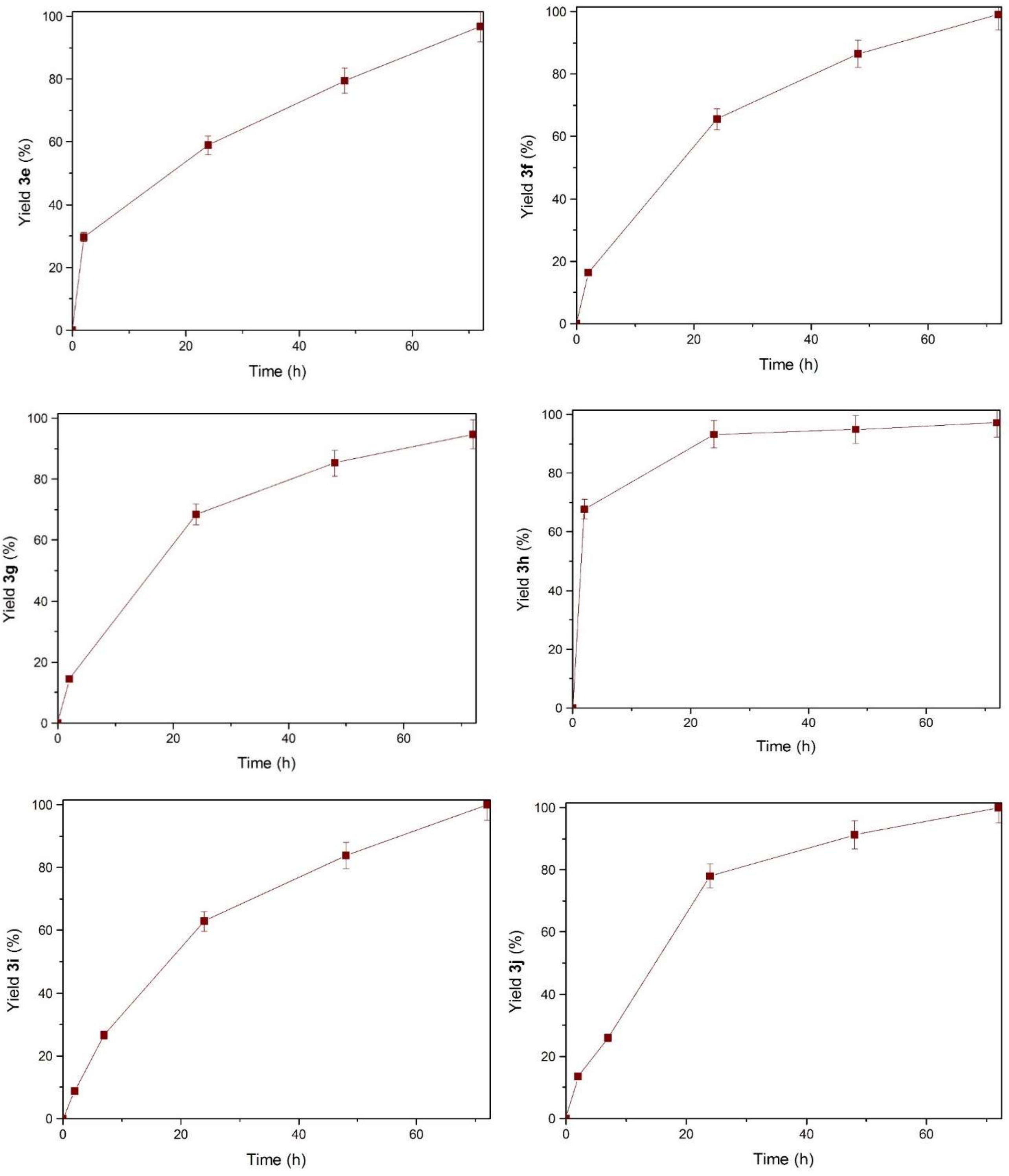

Figure S2. Kinetics for the one RAM contact-catalyzed one-pot esterification-hydration reaction of acyl chlorides **1a,c,e,g,i** with propargyl alcohol **2** and H_2O, at room temperature (20 ºC), under the indicated reaction conditions. GC yields. Error bars account for a 5% uncertainty.

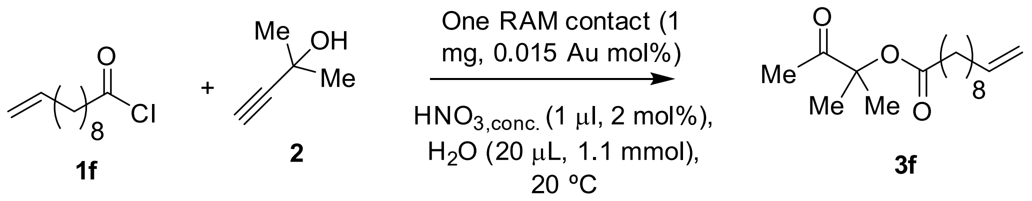

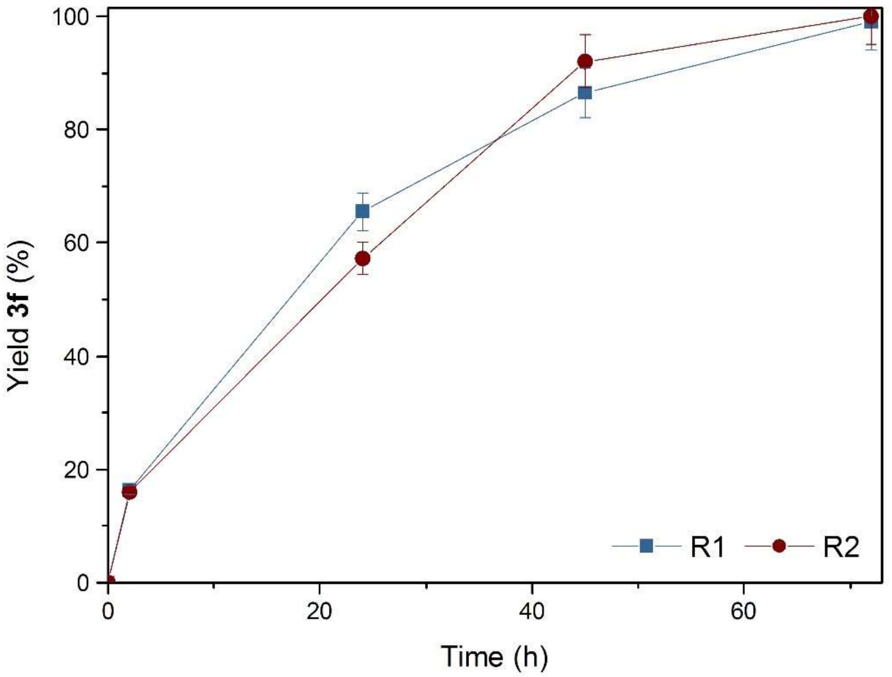

Figure S3. Kinetics for the one-pot esterification-hydration reaction of but-3-enoyl chloride **1f** with propargyl alcohol **2** and H_2O, catalyzed by two different contacts of a same RAM memory (R refers to the number of RAM contact) at room temperature (20 ºC), under the indicated reaction conditions. GC yields. Error bars account for a 5% uncertainty.

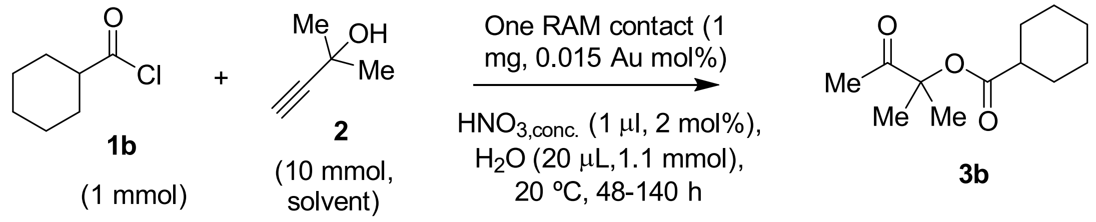

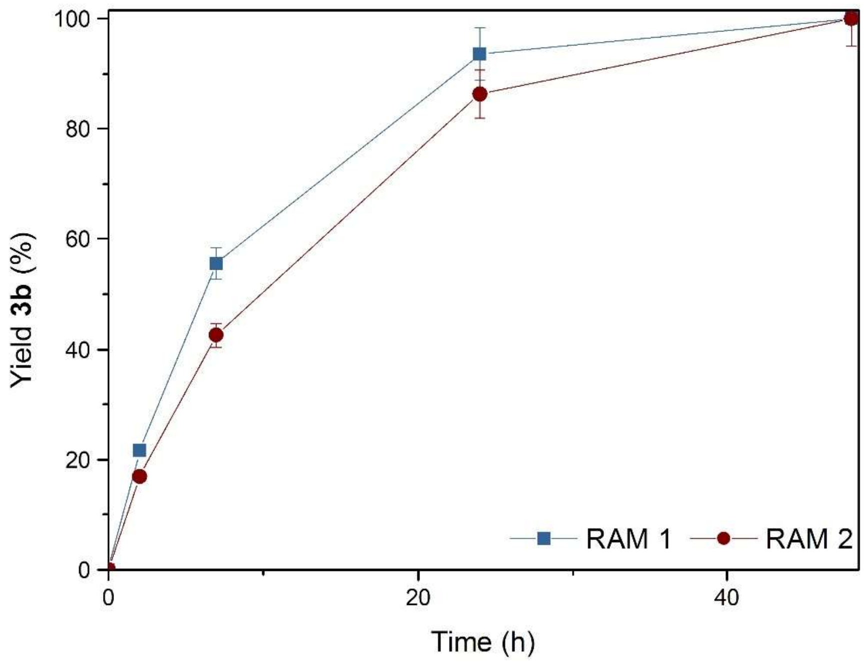

Figure S4. Kinetics for the one-pot esterification-hydration reaction of cyclohexanecarbonyl chloride **1b** with propargyl alcohol **2** and H_2O, catalyzed by one contact of two different RAM memories (R refers to the number of RAM memory) at room temperature (20 ºC), under the indicated reaction conditions. GC yields. Error bars account for a 5% uncertainty.

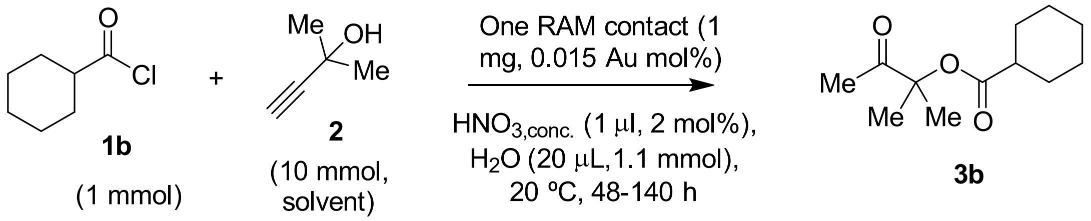

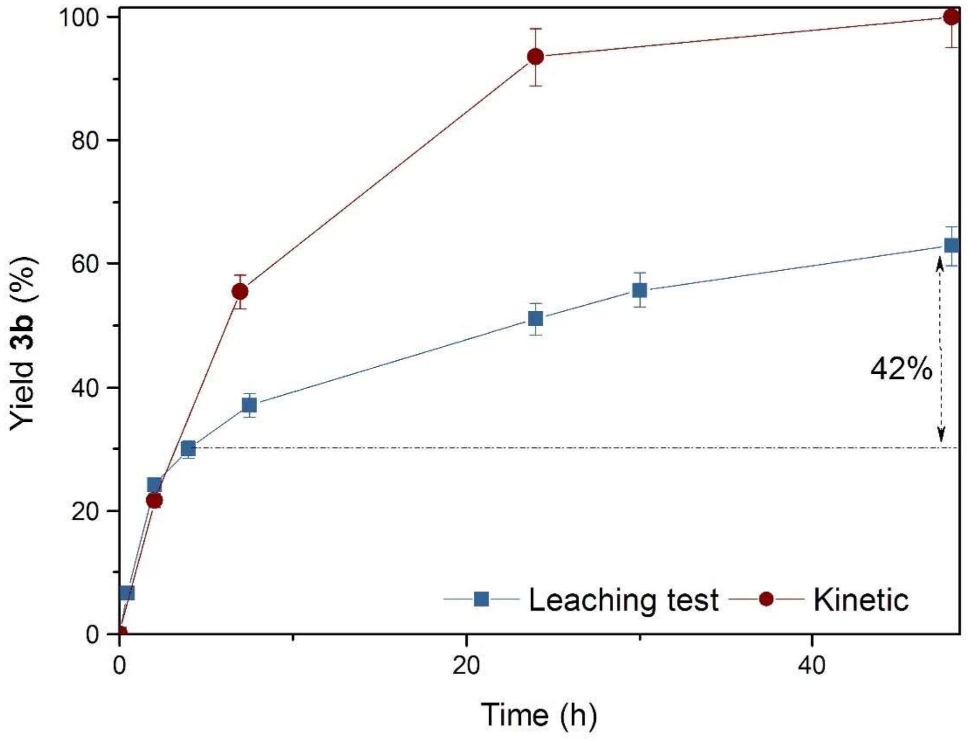

Figure S5. Kinetics for the leaching test during the one RAM contact-catalyzed one-pot esterification-hydration reaction of acyl chloride **1b** with propargyl alcohol **2** and H_2O, at room temperature (20 ºC), under the indicated reaction conditions. Filtration point for the parallel reaction (blue squares) is at 30% yield. GC yields. Error bars account for a 5% uncertainty.

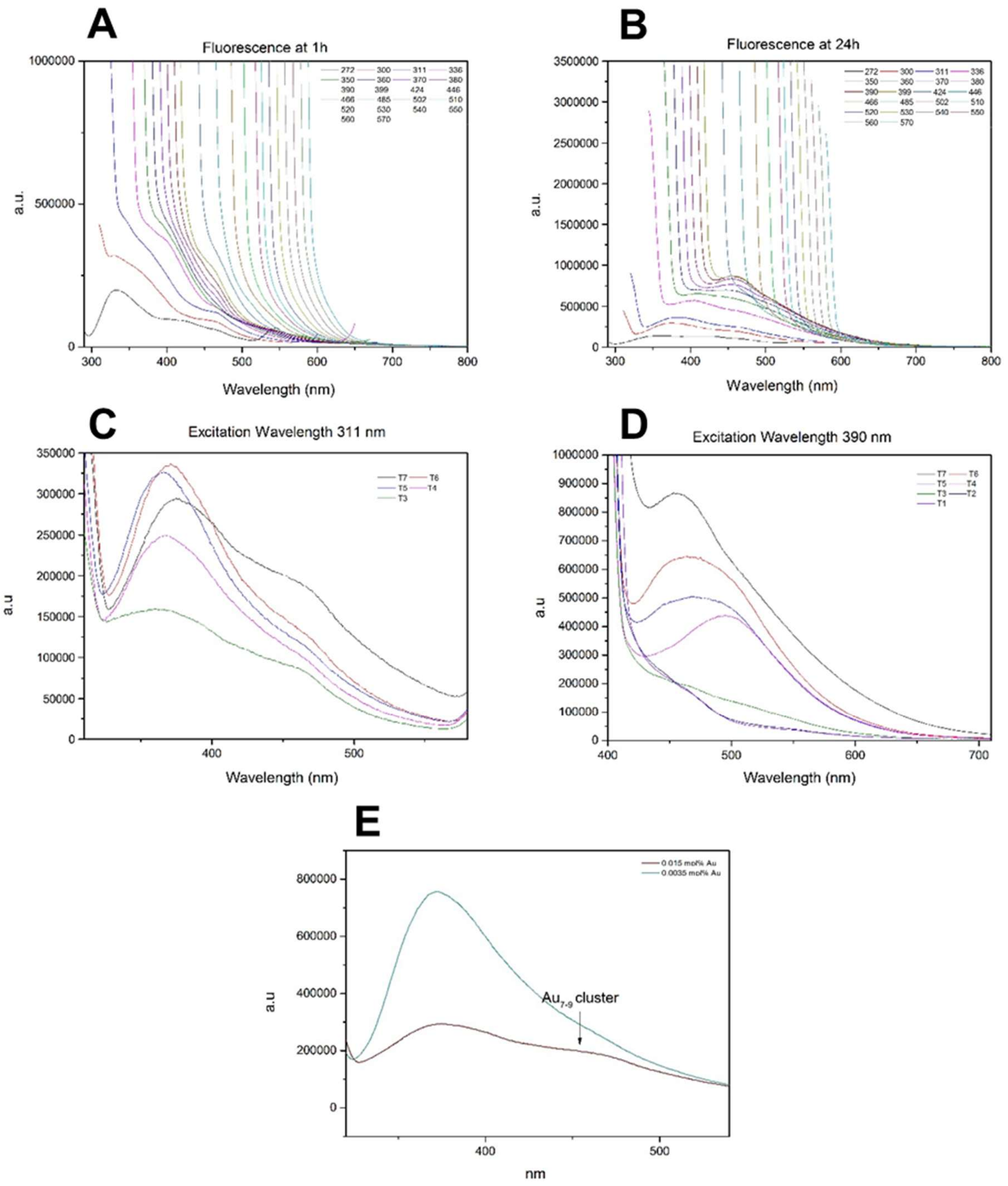

Figure S6. Fluorescence (emission ultraviolet-visible spectrophotometry) spectra of the Au species formed during the one-pot esterification-hydration reaction of 4-methoxybenzoyl chloride **1c** with propargyl alcohol **2** and H_2O, catalyzed by one RAM contact at room temperature (20 ºC), under the optimized reaction conditions (see Figures 2 and 3 in the main text). A) Fluorescense at 1 h reaction time. B) Fluorescense at 24 h reaction time. C) Fluorescense after irradiating at 311 nm for different times. D) Fluorescense after irradiating at 390 nm for different times. E) Comparison of the fluorescense after irradiating at 311 nm for different concentrations of Au [0.0035 mol% Au (blue) and 0.015 mol% Au (red)].

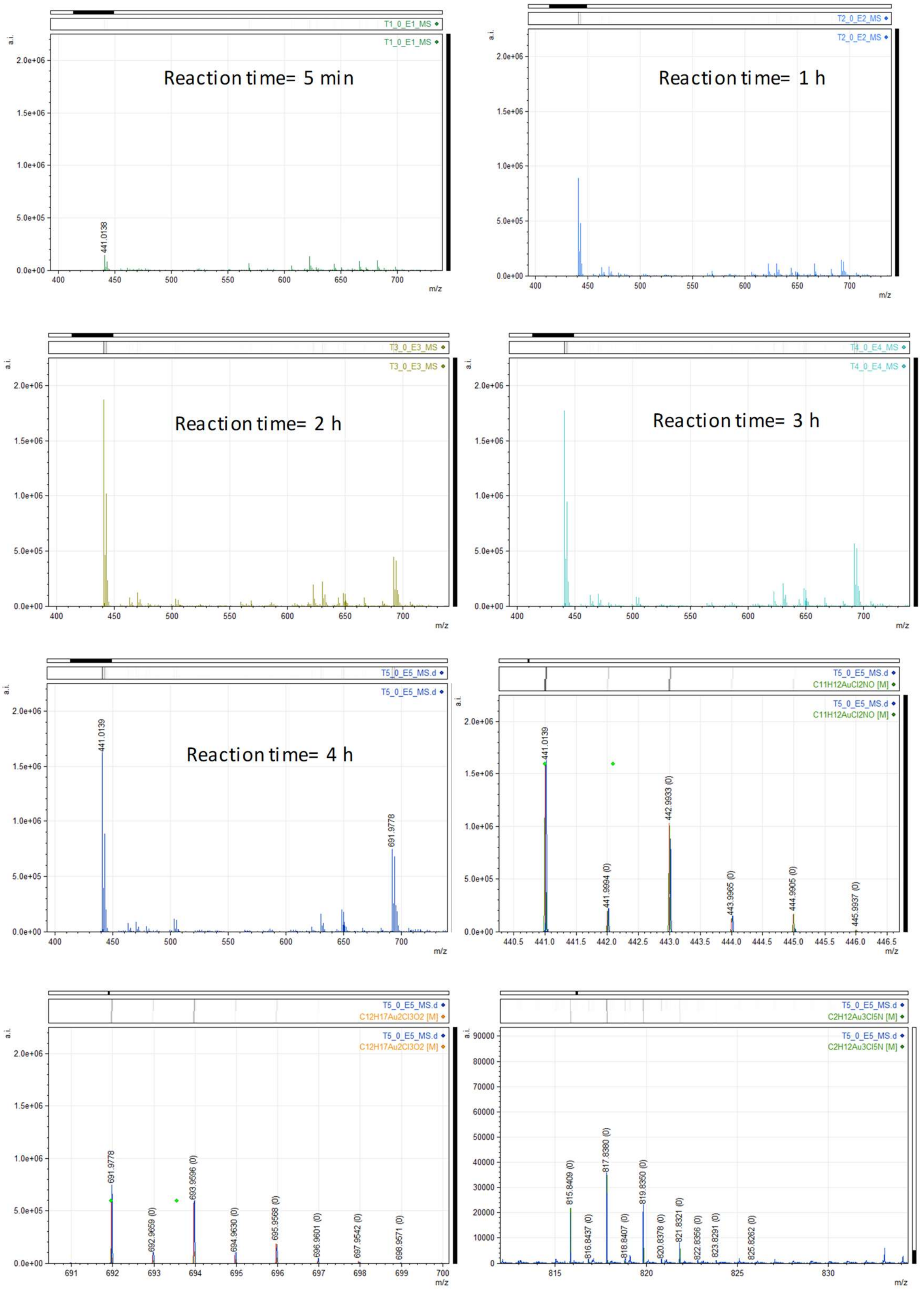

Figure S7. Matrix assisted laser desorption/ionization time-of-flight mass spectra (MALDI-TOF MS) of the Au species formed during the one-pot esterification-hydration reaction of 4-methoxybenzoyl chloride **1c** with propargyl alcohol **2** and H_2O, catalyzed by one RAM contact at room temperature (20 ºC), under the optimized reaction conditions and at different reaction times (see Figures 2 and 3 in the main text), after diluting in acetonitrile solvent, and simulated spectra for particular ultrasmall Au clusters, where Au is generally coordinated to **1c**, HCl, water and acetonitrile.

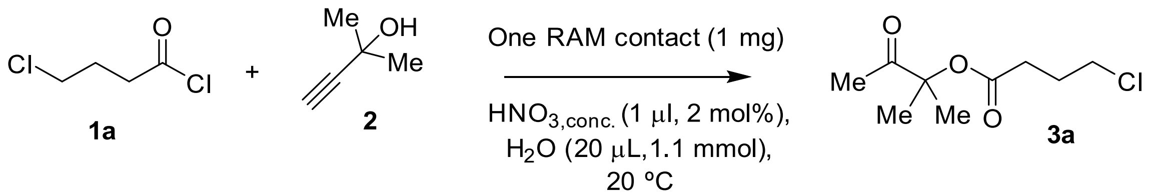

Entry	mmol 1a	mmol 2	Au (mol%)	t (h)	3a (yield, %)	TON
**1**	1	10	0.015	144	93	6560
**2**	3	30	0.0035	140	23	4315

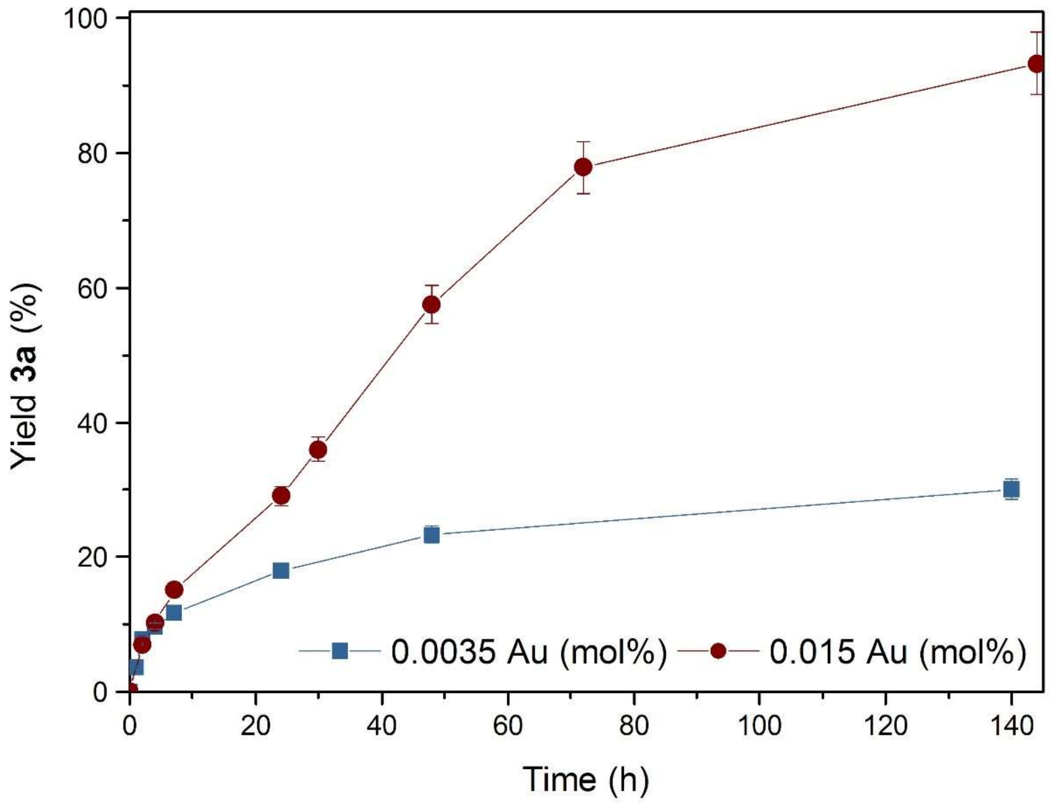

Figure S8. Results and kinetics for the one-pot esterification-hydration reaction of 4-chlorobutanoyl chloride **1a** with propargyl alcohol **2** and H_2O, catalyzed by one RAM contact at room temperature (20 ºC), under the indicated reaction conditions. GC yields. Error bars account for a 5% uncertainty.

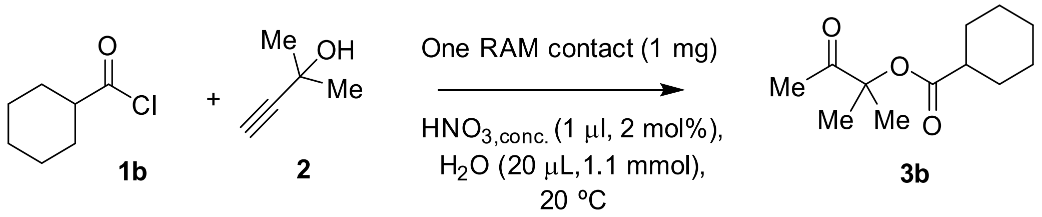

Entry	mmol 1b	mmol 2	Au (mol%)	t (h)	3b (yield, %)	TON
**1**	1	10	0.015	48	>99	6226
**2**	2	20	0.0075	48	68	9222

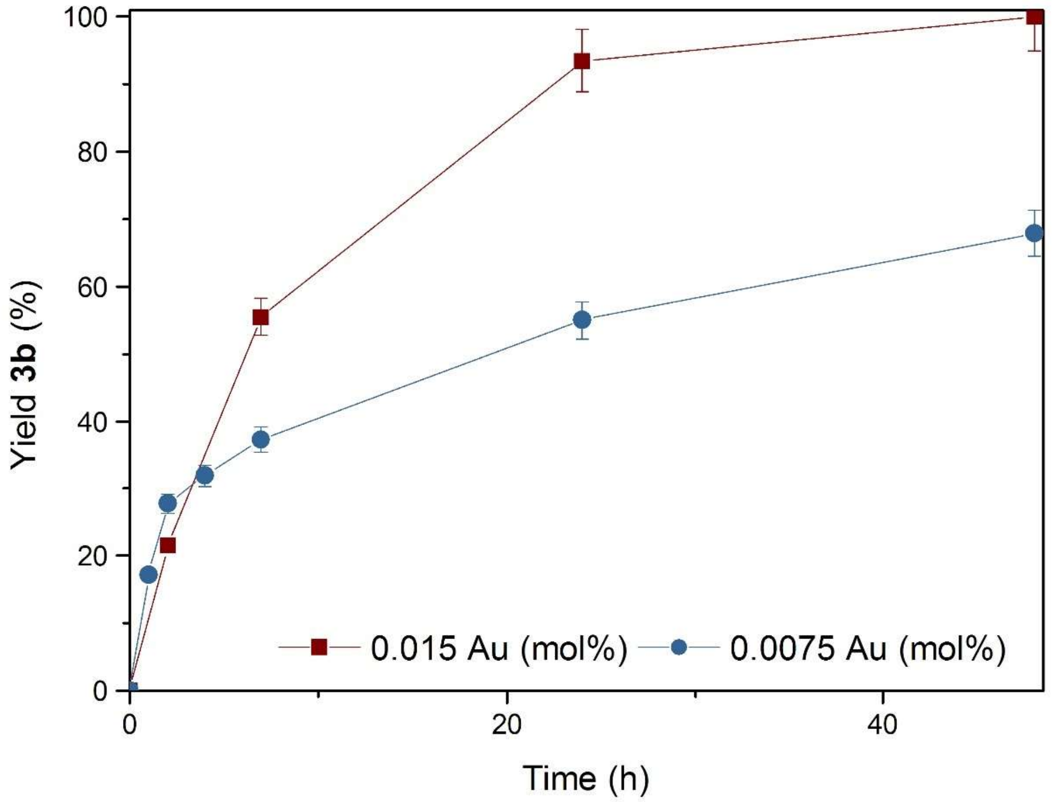

Figure S9. Results and kinetics for the one-pot esterification-hydration reaction of cyclohexanecarbonyl chloride **1b** with propargyl alcohol **2** and H_2O, catalyzed by one RAM contact, at room temperature (20 ºC), under the indicated reaction conditions. GC yields. Error bars account for a 5% uncertainty.

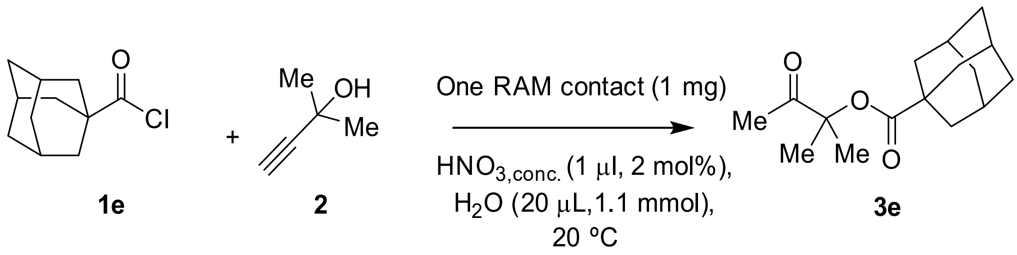

Entry	mmol 1e	mmol 2	Au (mol%)	t (h)	3e (yield, %)	TON
**1**	1	10	0.015	72	97	6411
**2**	2	20	0.0075	72	93	13530

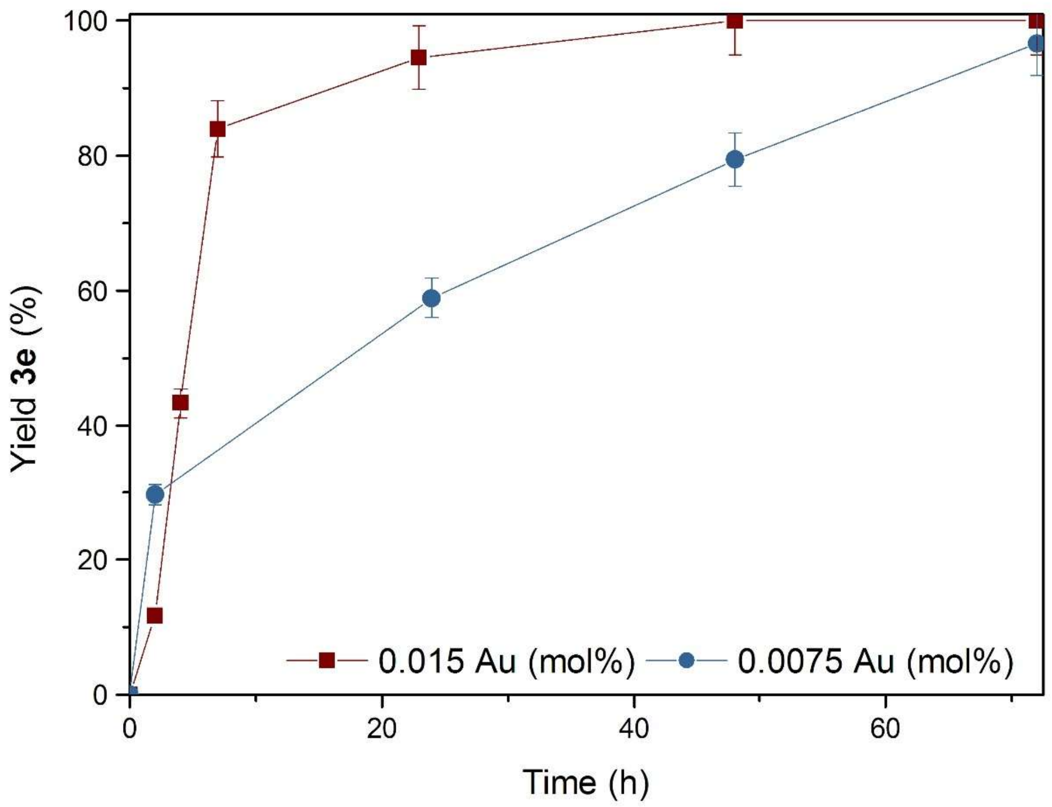

Figure S10. Results and kinetics for the one-pot esterifcation-hydration reaction of adamantane-1-carbonyl chloride **1e** with propargyl alcohol **2** and H_2O, catalyzed by one RAM contact, at room temperature (20 ºC), under the indicated reaction conditions. GC yields. Error bars account for a 5% uncertainty.

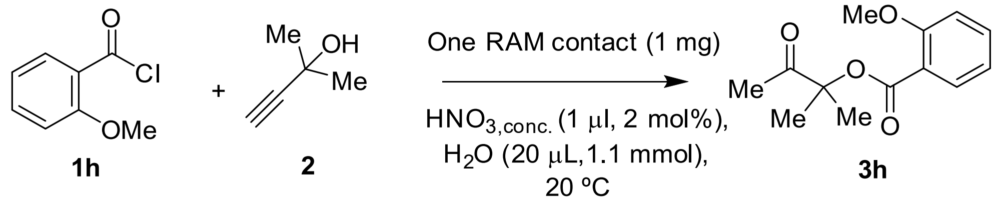

Entry	mmol 1h	mmol 2	Au (mol%)	t (h)	3h (yield, %)	TON
**1**	1	10	0.015	72	97	6357
**2**	3	30	0.005	48	94	19566

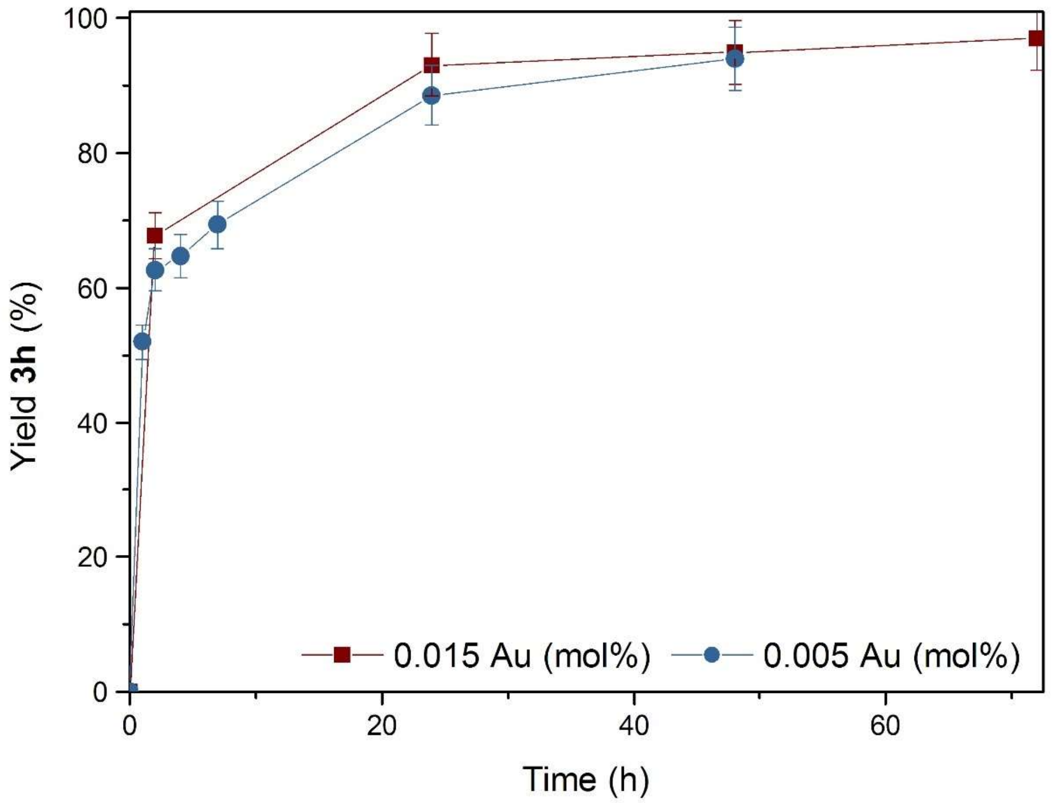

Figure S11. Results and kinetics for the one-pot esterification-hydration reaction of 2-methoxybenzoyl chloride **1h** with propargyl alcohol **2** and H_2O, catalyzed by one RAM contact, at room temperature (20 ºC), under the indicated reaction conditions. GC yields. Error bars account for a 5% uncertainty.

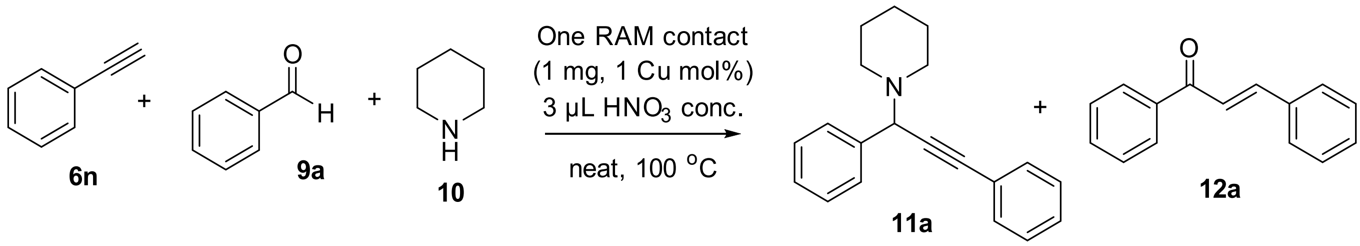

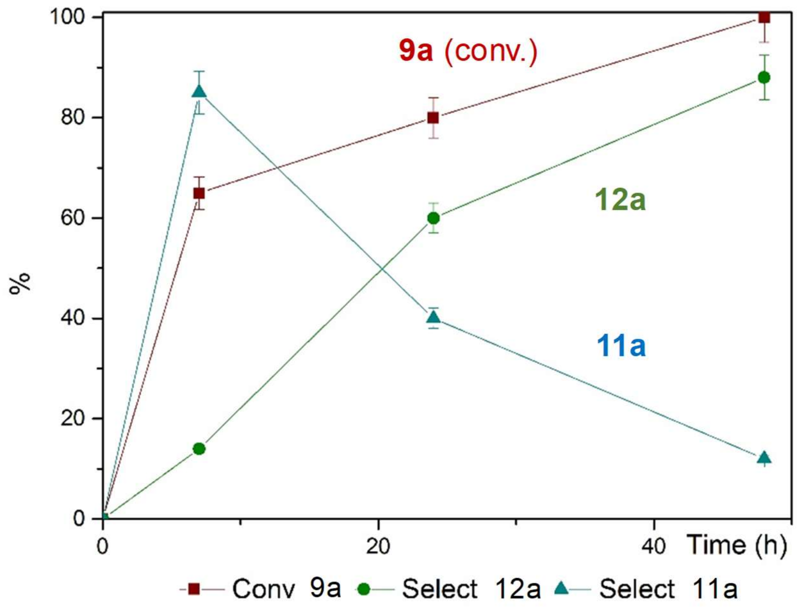

Figure S12. Kinetics for one RAM contact-catalyzed A^3 coupling reaction and concomitant Meyer–Schuster rearrangement of phenylacetylene **6n**, benzaldehyde **9a** and piperidine **10** under the indicated reaction conditions. GC yields. Error bars account for a 5% uncertainty.

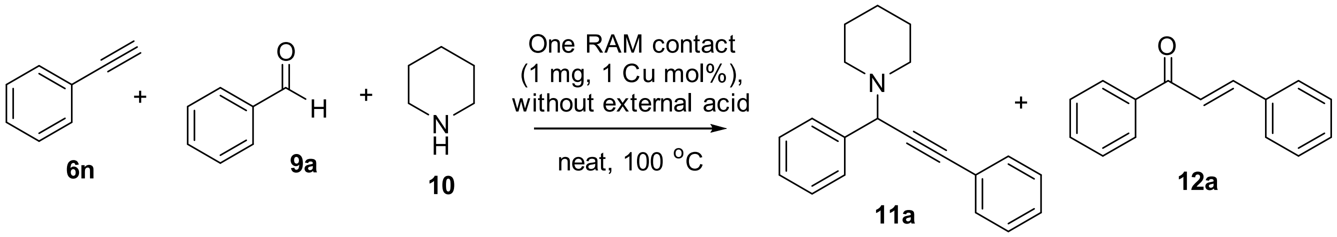

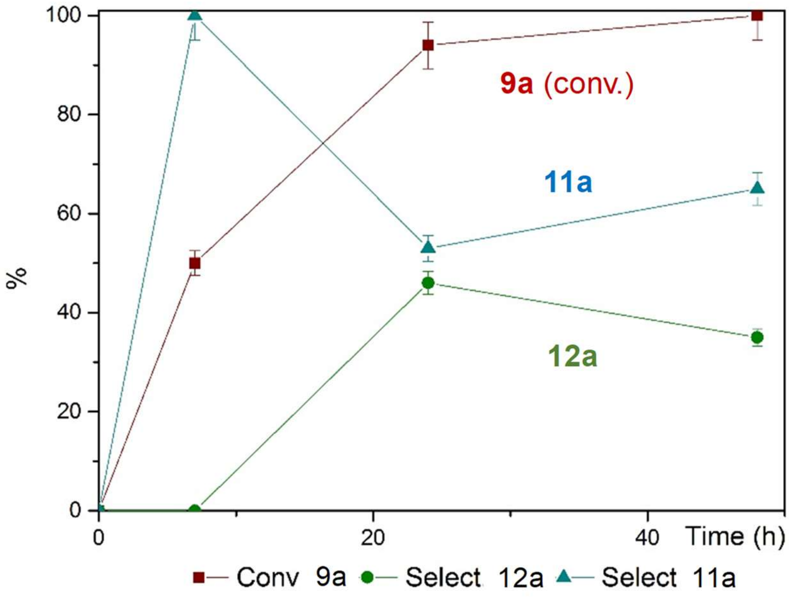

Figure S13. Kinetics for one RAM contact-catalyzed A^3 coupling reaction and concomitant Meyer–Schuster rearrangement of phenylacetylene **6n**, benzaldehyde **9a** and piperidine **10** without any added external acid, under the indicated reaction conditions. GC yields. Error bars account for a 5% uncertainty.

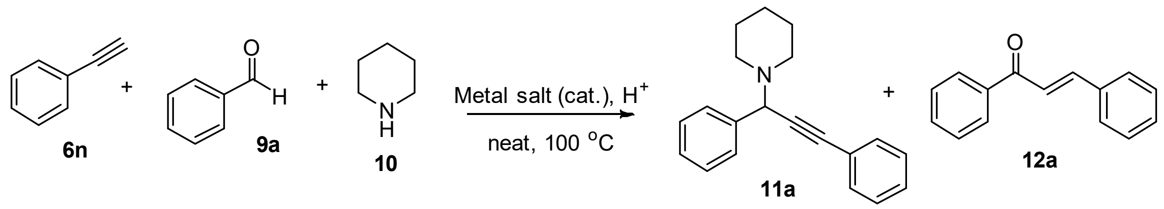

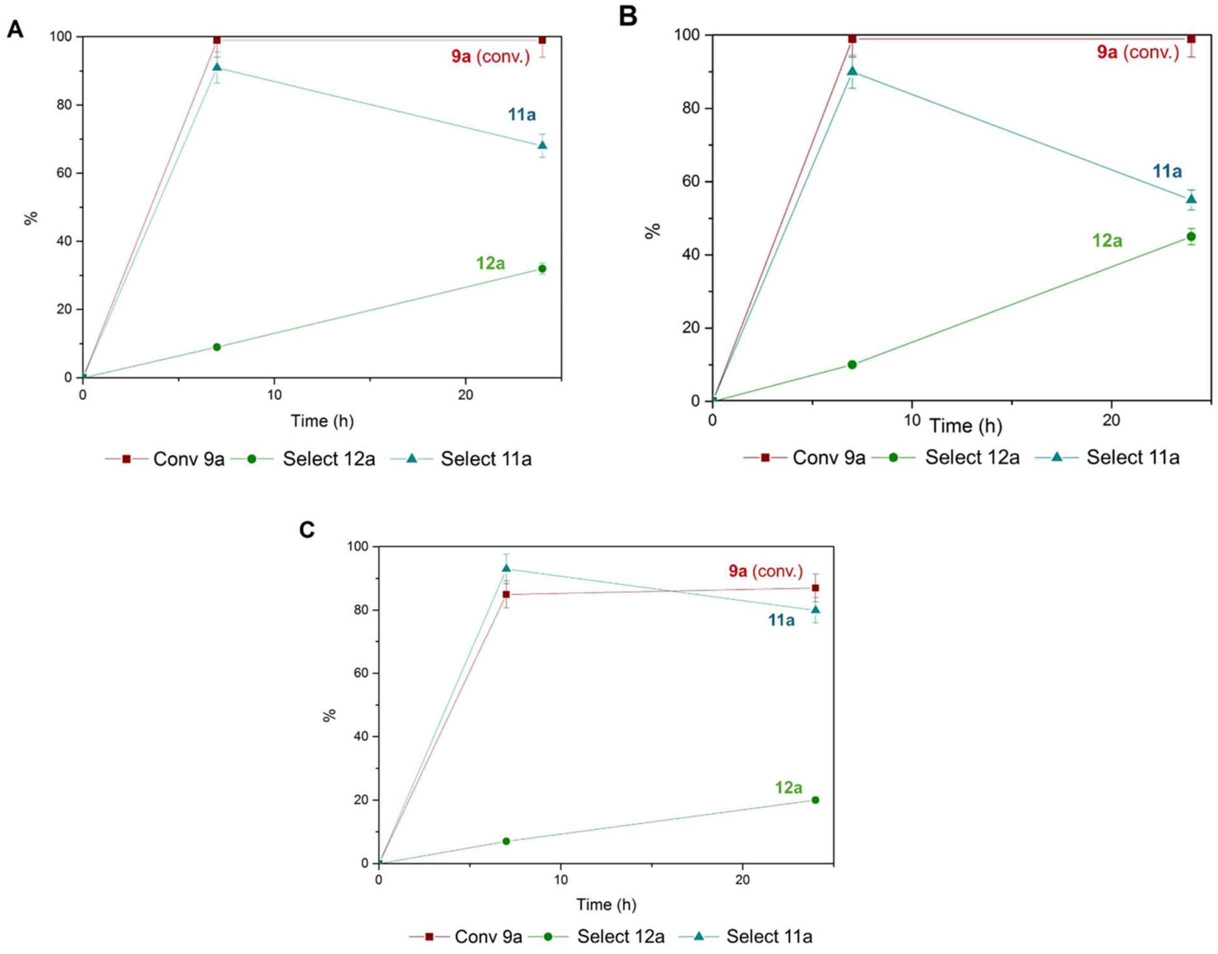

Figure S14. Results for the A[3] coupling reaction and concomitant Meyer–Schuster rearrangement of phenylacetylene **6n**, benzaldehyde **9a** and piperidine **10** with $CuCl_2$ (1 mol%, **A**), $HAuCl_4$ (0.015 mol%, **B**) and Cu extracts from one RAM contact (1 mol%, **C**), under the indicated reaction conditions. GC yields. Error bars account for a 5% uncertainty.

Supporting Tables

Table S1. Inductively coupled plasma optical emission spectroscopy *(*ICP-OES*)* analysis of the RAM contacts.

Entry	Metal	wt%
1	**Sn**	0.19
2	**Zn**	0.12
3	**Ni**	10.64
4	**Pb**	0.00
5	**Au**	3.51
6	**Mn**	0.00
7	**Fe**	0.01
8	**Cu**	74.32
9	**Ag**	0.10
10	**Ti**	0.03
11	**Pd**	0.02
12	**Al**	0.05
13	**Ba**	0.11
		89.10

Table S2. Inductively coupled plasma optical emission spectroscopy (ICP-OES) analysis of the Au present in three different RAM contacts.

Entry	m Contacts (mg)	Au (ppm)	Au (wt%)
1	51.6	34,680.414	3.468
2	50.7	35,010.253	3.510
3	53.7	35,018.161	3.502
			3.490±0.019

Table S3. Catalytic results with HNO_3, $Cu(NO_3)_2$, $Ni(NO_3)_2$, and $HAuCl_4$ for the one-pot reaction between 4-chlorobutanoyl chloride **1a** (1 mmol), 2-methylbut-3-yn-2-ol **2** (10 mmol, solvent) and water (1.1 mmol), under the indicated acidic reaction conditions.

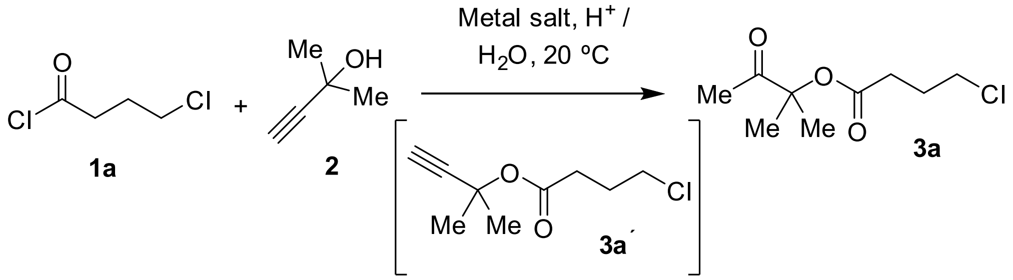

Entry	Catalyst	mol%	3a (yield, %)
**1**	$HAuCl_4$	0.015	85
**2**	$Ni(NO_3)_2$	0.16	24
**3**	$Cu(NO_3)_2$	1.0	21
**4**	HCl	2.0	18

SUPPORTING INFORMATION

Table S4. Detailed scope for the RAM contact-catalyzed one-pot esterification-hydration reaction of acyl chlorides **1a-j** with propargyl alcohol **2** and H_2O, at room temperature (20 ºC), under the indicated reaction conditions. Isolated yields.

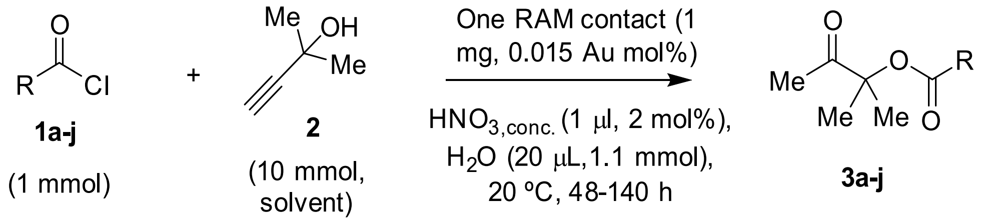

Entry		Substrate 1	Au (mol%)	Time (h)		Product 3	Yield (%)	TON
**1**	**1a**	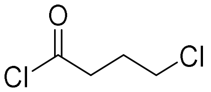	0.015	140	**3a**	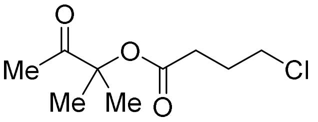	**93**	6560
**2**	**1b**	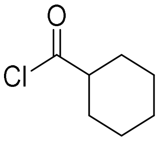	0.015	48	**3b**	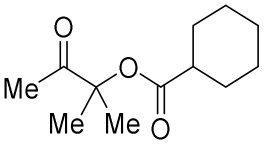	**>99**	6226
**3**	**1c**	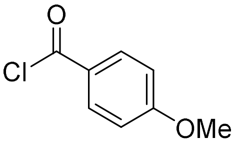	0.015	140	**3c**	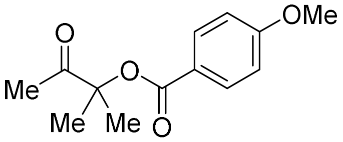	**97**	6029
**4**	**1d**	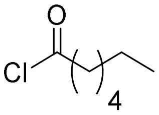	0.016	72	**3d**	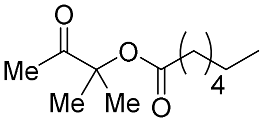	**>99**	6219
**5**	**1e**	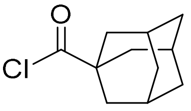	0.015	72	**3e**	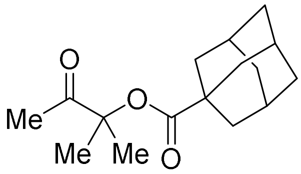	**97**	6411
**6**	**1f**	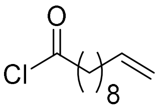	0.016	72	**3f**	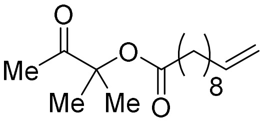	**99**	6492
**7**	**1g**	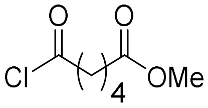	0.015	72	**3g**	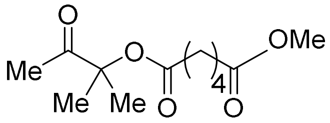	**95**	6661
**8**	**1h**	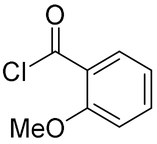	0.015	72	**3h**	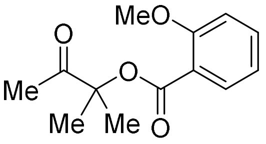	**97**	6353
**9**	**1i**	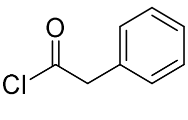	0.015	72	**3i**	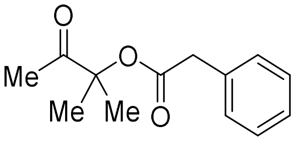	**>99**	6268
**10**	**1j**	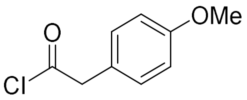	0.015	72	**3j**	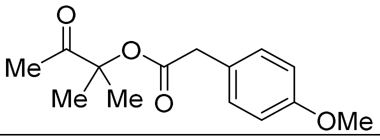	**>99**	6630

.

Table S5. Weight difference after using the RAM contacts as a catalyst for the one-pot reaction between 4-chlorobutanoyl chloride **1a** (1 mmol), 2-methylbut-3-yn-2-ol **2** (10 mmol, solvent) and water (1.1 mmol), under the indicated acidic reaction conditions.

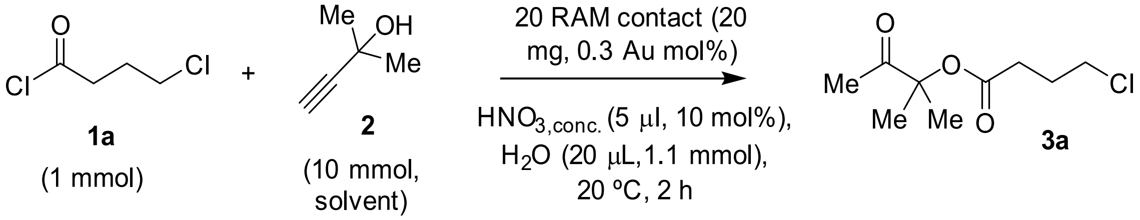

Entry	Use	m Contacts (mg)	Yield (%)
**1**	Use 1	20.1	95
**2**	Use 2	16.4	87
**3**	Use 3	10.3	85
**4**	Use 4	8.2	83
**5**	Use 5	1.4	34

Table S6. Catalytic results for the one-pot esterification-hydration reaction of 4-methoxybenzoyl chloride **1c** with propargyl alcohol **2** and H_2O, catalyzed by different amounts of the acidic solution of disaggregated RAM contacts, at room temperature (20 ºC), under the indicated reaction conditions. GC yields.

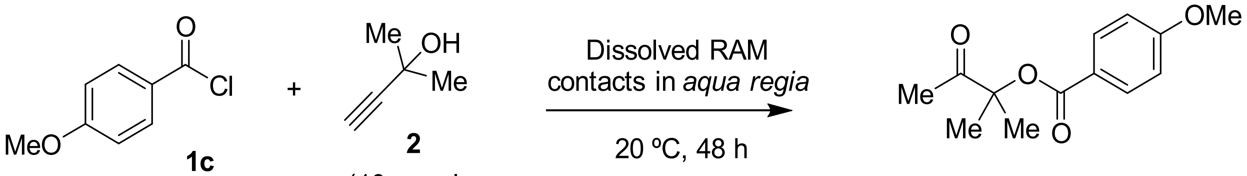

1c (1 mmol) **2** (10 mmol, solvent) **3c**

Entry	Catalyst	Volume of solution (µL)	Au mol%	3c (yield, %)	TON
**1**	AuCl	-	0.0005	81	161990
**2**	Dissolved	1.0 (+ 7.0 H_2O)	0.0005	41	81995
**3**	contacts	1.5	0.00075	76	101327
**4**		1.0	0.0005	82	163990
**5**		0.5	0.00025	88	351979
**6**		0.4	0.0002	86	429975
**7**		0.2	0.00015	86	573300

Table S7. Detailed scope for the RAM contact-catalyzed intramolecular hydroamination reaction of aromatic alkynes **4a-d** to form indoles **5a-c** in acetonitrile solvent at 80 ºC, under the indicated reaction conditions. Isolated yields.

Entry		Substrate	Au (mol%)		Product	Yield (%)	TON
**1**	**4a**	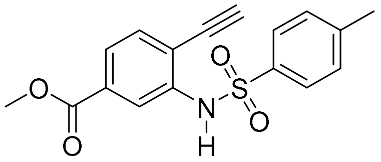	0.015	**5a**	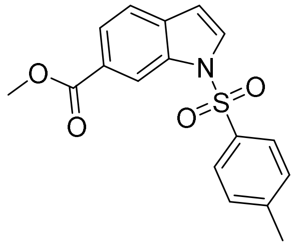	**>99**	6560
**2**	**4b**	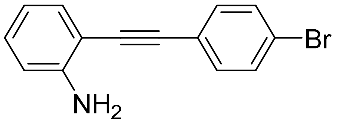	0.015	**5b**	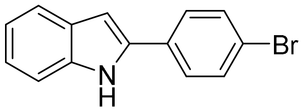	**>99**	6560
**3**	**4c**	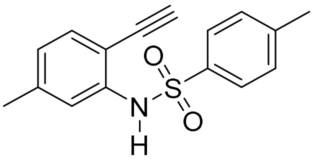	0.015	**5c**	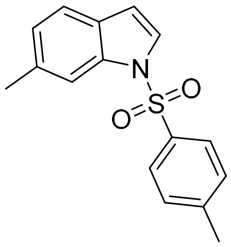	**>99**	6560
**4**	**4d**	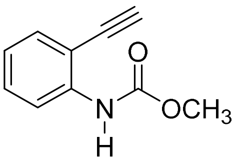	0.016	**5d**	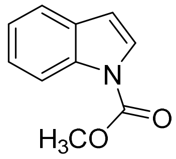	**0**	0

Table S8. Detailed scope for the RAM contact-catalyzed hydration or hydrochlorination reaction of alkynes **6a-m**, under the indicated reaction conditions. GC yields.

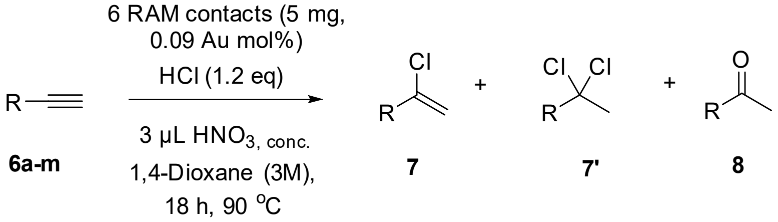

Entry		Substrate	Au (mol%)	Yield (7+7') (%)	Yield 8 (%)	TON
**1**	**6a**	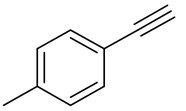	0.09	**0**	**>99**	1062
**2**	**6b**	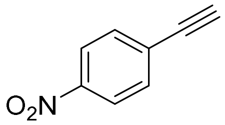	0.10	**0**	**>99**	1005
**3**	**6c**	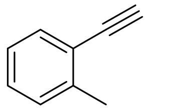	0.10	**84**	**16**	1042
**4**	**6d**	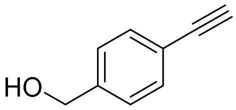	0.10	**54**	**46**	1023
**5**	**6e**	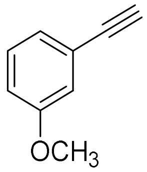	0.10	**96**	**4**	1023
**6**	**6f**	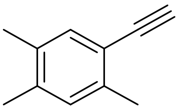	0.10	**54**	**46**	1005
**7**	**6g**	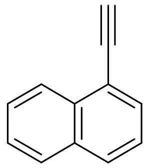	0.10	**63**	**37**	1005
**8**	**6h**	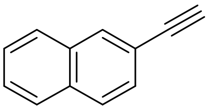	0.09	**0**	**>99**	1062
**9**	**6i**	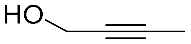	0.09	**>99**	**0**	1082
**10**	**6j**	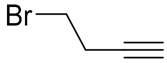	0.10	**>99**	**0**	1023
**11**	**6k**	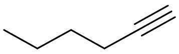	0.10	**>99 (7/7':81/19)**	**0**	1005
**12**	**6l**	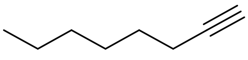	0.10	**94 (7/7':75/25)**	**6**	1042
**13**	**6m**	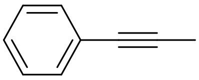	0.10	**82**	**18**	1005

Compound characterization

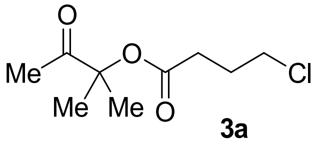

3a

2-Methyl-3-oxobutan-2-yl 4-chlorobutanoate (3a). Yield: 93.0%. ^{1}H NMR (300 MHz, $CDCl_3$): δ = 3.59 ($ClCH_2$, 2H, dd, J = 6.3, 6.3 Hz), 2.52 ($COCH_2$, 2H, dd, J = 7.2, 6.9 Hz), 2.10 ($COCH_3$, 3H, s), 2.09-2.02 (aliphatic CH_2, 2H, m), 1.46 (CH_3, 6H, s) ppm. ^{13}C NMR (75 MHz, $CDCl_3$): δ = 206.66 (ketone, C), 172.11 (ester, C), 83.96 (aliphatic, C), 44.00 (aliphatic, CH_2Cl), 31.37 (aliphatic, $COCH_2$), 27.54 (aliphatic, CH_2), 23.82 (methyl, $COCH_3$), 23.54 (methyl, $2CH_3$) ppm.

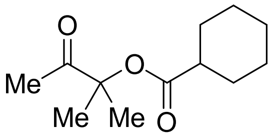

3b

2-Methyl-3-oxobutan-2-yl cyclohexanecarboxylate (3b). Yield: >99.0%.^{1}H NMR (300 MHz, $CDCl_3$): δ = 2.37-2.24 (CHCO, 1H, m), 2.07 ($COCH_3$, 3H, s), 1.95-1.83 (aliphatic CH_2, 2H, m), 1.80-1.68 (aliphatic CH_2, 2H, m), 1.52-1.44 (aliphatic CH_2, 2H, m), 1.43 (CH_3, 6H, s), 1.38-1.11 (aliphatic CH_2, 4H, m) ppm. ^{13}C NMR (75 MHz, $CDCl_3$): δ = 207.09 (ketone, C), 175.47 (ester, C), 83.33 (aliphatic, C), 43.17 (cyclohexane, CH), 28.91 (cyclohexane, $2CH_2$), 25.79 (cyclohexane, CH_2), 25.42 (cyclohexane, $2CH_2$), 23.56 (methyl, $COCH_3$), 23.43 (methyl, $2CH_3$) ppm.

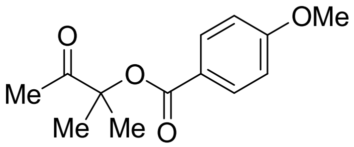

3c

2-Methyl-3-oxobutan-2-yl 4-methoxybenzoate (3c). Yield: 97.0%. ^{1}H NMR (300 MHz, $CDCl_3$): δ = 7.99 (CH, 2H, ddd, J = 7.2, 2.1, 2.1 Hz), 6.92 (CH, 2H, ddd, J = 9.0, 2.1, 2.1 Hz), 3.87 (OCH_3, 3H, s), 2.16 ($COCH_3$, 3H, s), 1.58 (aliphatic CH_3, 6H, s) ppm. ^{13}C NMR (75 MHz, $CDCl_3$): δ = 207.30 (ketone, C), 165.68, (aromatic, C), 163.83 (ester, C), 132.01 (aromatic, 2CH), 122.27 (aromatic, C), 113.84 (aromatic, 2CH), 84.03 (aliphatic, C), 55.61 (aliphatic, OCH_3), 23.70 (methyl, $COCH_3$), 23.61 (methyl, $2CH_3$) ppm.

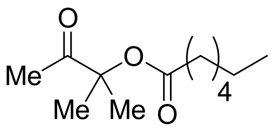

3d

2-Methyl-3-oxobutan-2-yl heptanoate (3d). Yield: >99.0%. ^{1}H NMR (300 MHz, $CDCl_3$): δ = 2.30 ($COCH_2$, 2H, dd, J = 9.6, 9.6 Hz), 2.08 ($COCH_3$, 3H, s), 1.67-1.53 (aliphatic CH_2, 2H, m), 1.43 ($2CH_3$, 6H, s), 1.34-1.21 (aliphatic CH_2, 6H, m), 0.91-0.80 (aliphatic CH_3, 3H, m) ppm. ^{13}C NMR (75 MHz, $CDCl_3$): δ = 206.98 (ketone, C), 173.28 (ester, C), 83.52 (aliphatic, C), 34.46 (aliphatic, CH_2), 31.5 (aliphatic, CH_2), 28.84 (aliphatic, CH_2), 24.89 (aliphatic, CH_2), 23.65 (methyl, $COCH_3$), 23.46 (methyl, $2CH_3$), 22.53 (aliphatic, CH_2), 14.07 (methyl, CH_3) ppm.

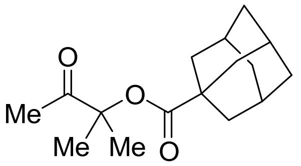

3e

2-Methyl-3-oxobutan-2-yl-adamantane-1-carboxylate (3e). Yield: 97.0%. ^{1}H NMR (300 MHz, $CDCl_3$): δ = 2.05 ($COCH_3$, 3H, s), 2.03-2.01 (aliphatic CH, 3H, m), 1.92-1.87 (aliphatic CH_2, 6H, m), 1.73-1.70 (aliphatic CH_2, 6H, m), 1.42 (aliphatic CH_3, 6H, s) ppm. ^{13}C NMR (75 MHz, $CDCl_3$): δ = 207.2 (ketone, C), 177.0 (ester, C), 83.2 (aliphatic, C), 40.7 (aliphatic, C), 38.9 (aliphatic, $3CH_2$), 36.6 (aliphatic, $3CH_2$), 28.0 (aliphatic, 3CH), 23.4 (methyl, $COCH_3$), 23.31 (aliphatic, $2CH_3$) ppm.

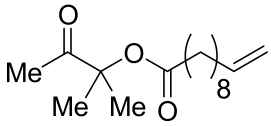

3f

2-Methyl-3-oxobutan-2-yl undec-10-enoate (3f). Yield: 99.0%. ^{1}H NMR (300 MHz, $CDCl_3$): δ = 5.87-5.69 ($CH=CH_2$, 1H, m), 5.03-4.85 ($CH=CH_2$, 2H, m), 2.30 ($COCH_2$, 2H, dd, J = 7.8, 7.2 Hz), 2.09 ($COCH_3$, 3H, s), 2.01 (aliphatic CH_2, 2H, dd, J = 3.0, 3.0 Hz), 1.67-1.55 (aliphatic CH_2, 2H, m), 1.44 (CH_3, 6H, s), 1.37-1.22 (aliphatic CH_2, 10H, m) ppm. ^{13}C NMR (75 MHz, $CDCl_3$): δ = 207.02 (ketone, C), 173.28 (ester, C), 139.21 ($C\mathit{H}=CH_2$, CH), 114.26 ($CH=C\mathit{H}_2$, CH_2), 83.52 (aliphatic, C), 34.45 (aliphatic, CH_2), 33.87 (aliphatic, CH_2), 29.35 (aliphatic, CH_2), 29.27 (aliphatic, CH_2), 29.16 (aliphatic, CH_2), 29.12 (aliphatic, CH_2), 28.96 (aliphatic, CH_2), 24.92 (aliphatic, CH_2), 23.67 (methyl, $COCH_3$), 23.47 (methyl, $2CH_3$) ppm.

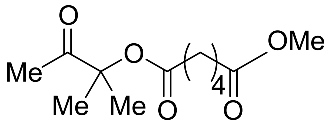

3g

Methyl (2-methyl-3-oxobutan-2-yl) adipate (3g). Yield: 95.0%. ^{1}H NMR (300 MHz, $CDCl_3$): δ = 3.66 ($-OCH_3$, 3H, s), 2.37-2.31 (CH_2, 4H, m), 2.09 ($COCH_3$, 3H, s), 1.68-1.64 (CH_2, 4H, m), 1.45 (CH_3, 6H, s) ppm. ^{13}C NMR (75 MHz, $CDCl_3$): δ = 206.9 (ketone, C), 173.8 (ester, C), 172.8 (ester, C), 83.7 (aliphatic, C), 51.7 (-OMe), 34.1 (aliphatic, CH_2), 33.7 (aliphatic, CH_2), 24.4 (aliphatic, CH_2), 24.4 (aliphatic, CH_2), 23.8 (methyl, $COCH_3$), 23.5 (aliphatic, $2CH_3$) ppm.

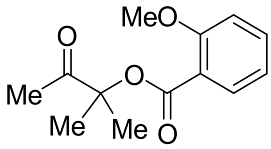

3h

2-Methyl-3-oxobutan-2-yl 2-methoxybenzoate (3h). Yield: 97.0%. ^{1}H NMR (300 MHz, $CDCl_3$): δ = 7.80 (CH, 1H, dm, J = 7.8 Hz), 7.48 (CH, 1H, m), 6.98 (CH, 2H, m), 3.88 (OCH_3, 3H, s), 2.19 ($COCH_3$, 3H, s), 1.56 (aliphatic CH_3, 6H, s) ppm. ^{13}C NMR (75 MHz, $CDCl_3$): δ = 207.25 (ketone, C), 165.40 (ester, C), 159.60 (aromatic, C), 134.04 (aromatic, CH), 131.73 (aromatic, CH), 120.20 (aromatic, CH), 119.59 (aromatic, C), 112.22 (aromatic, CH), 84.21 (aliphatic, C), 56.02 (aliphatic, OCH_3), 23.67 (methyl, $COCH_3$), 23.5 (methyl, $2CH_3$) ppm.

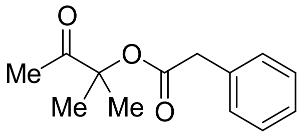

3i

2-Methyl-3-oxobutan-2-yl 2-phenylacetate (3i). Yield: >99.0%. ^{1}H NMR (300 MHz, $CDCl_3$): δ = 7.29-7.19 (aromatic CH, 5H, m), 3.58 (COCH, 2H, s), 1.93 ($COCH_3$, 3H, s), 1.29 (CH_3, 6H, s) ppm. ^{13}C NMR (75 MHz, $CDCl_3$): δ = 206.7 (ketone, C), 170.9 (ester, C), 133.6 (aromatic, C), 129.3 (aromatic, CH), 128.7 (aromatic, CH), 127.3 (aromatic, CH), 84.2 (aliphatic, C), 41.6 (aliphatic, CH_2), 23.5 (methyl, $COCH_3$), 23.4 (aliphatic, $2CH_3$) ppm.

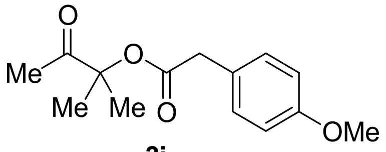

3j

2-Methyl-3-oxobutan-2-yl 2-(4-methoxyphenyl)acetate (3j). Yield: >99.0%. ^{1}H NMR (300 MHz, $CDCl_3$): δ = 7.22 (CH, 2H, dm, J = 7.2 Hz), 6.87 (CH, 2H, dm, J = 8 Hz), 3.79 (OCH_3, 3H, s), 3.57 ($COCH_2$, 2H, s), 1.99 ($COCH_3$, 3H, s), 1.44 (CH_3, 6H, s) ppm. ^{13}C NMR (75 MHz, $CDCl_3$): δ = 206.85 (ketone, C), 171.25 (ester, C), 158.91 (aromatic, C), 130.36 (aromatic, 2CH), 125.63 (aromatic, C), 114.17 (aromatic, 2CH), 84.18 (aliphatic, C), 55.36 (aliphatic, OCH_3), 40.71 (aliphatic, CH_2), 23.59 (methyl, $COCH_3$), 23.42 (methyl, $2CH_3$) ppm.

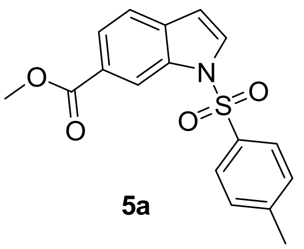

5a

Methyl 1-tosyl-1*H*-indole-6-carboxylate (5a). Yield: >99.0%.^{1}H NMR (401 MHz, $CDCl_3$): 8.66 (indole CH, 1H, s), 7.88 (indole CH ,1H, m), 7.76 (toluene CH, 2H, dm, J = 7.7 Hz), 7.68 (indole CH, 1H, m), 7.52 (indole CH, 1H, m), 7.20 (toluene CH, 2H, dm, J = 7.2 Hz), 6.67 (indole CH, 1H, m), 3.93 (OCH_3, 3H, s), 2.30 (touene CH_3, 3H, s) ppm. ^{13}C NMR (101 MHz, $CDCl_3$): 167.28 (ester, C), 145.38 (aromatic, C), 135.05 (indole, C), 134,46 (indole, C), 134,31 (aromatic, C), 130.08 (aromatic, 2CH), 129.31 (indole, CH), 126.92 (aromatic, 2CH), 126.48 (indole, C), 124.46 (indole, CH), 121.16 (indole, CH), 115.27 (indole, CH), 108.7 (indole, CH), 52.28 (aliphatic, OCH_3), 21.58 (methyl, CH_3) ppm.

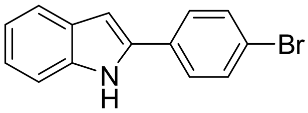

5b

2-(4-Bromophenyl)-1*H*-indole (5b). Yield: >99.0%.^{1}H NMR (401 MHz, $CDCl_3$): 8.35 (N-H, 1H, s), 7.62 (5H, m), 7.47 (1H, d), 7.36 (2H, m), 6.82 (1H, s) ppm.^{13}C NMR (101 MHz, $CDCl_3$): 137.07 (C), 133.27 (C), 133.05 (CH), 132.28 (CH), 131.77 (C), 131.46 (C), 130.92 (CH), 126.74 (CH), 122.85 (CH), 122.55 (C), 120.9 (CH), 120.6 (CH), 111.11 (CH), 100.65 (CH) ppm.

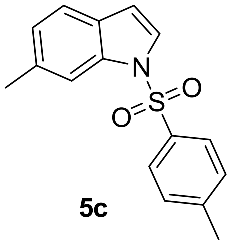

5c

6-Methyl-1-tosyl-1*H*-indole (5c). Yield: >99.0%. ^{1}H NMR (401 MHz, $CDCl_3$): 7.80 (indole CH, 1H, m), 7.74 (toluene CH , 2H, m), 7.48 (indole CH, 1H, m), 7.38 (indole CH, 1H, m), 7.20 (toluene CH, 2H, m), 7.06 (indole CH, 1H, m), 6.59 (indole CH, 1H, m), 2.47 (indole CH_3, 3H, s), 2.33 (toluene CH_3, 3H, s) ppm. ^{13}C NMR (101 MHz, $CDCl_3$): 144.92 (aromatic, C), 135.61 (aromatic, C), 135.39 (indole, C), 134.82 (indole, C), 129.98 (aromatic, 2CH), 128.57 (indole, C), 126.88 (aromatic, 2CH), 125.83 (indole, CH), 124.96 (indole, CH), 121.02 (indole, CH), 113.75 (indole, CH), 109.06 (indole, CH), 22.07 (indole, CH_3), 21.67 (methyl, CH_3) ppm.

NMR spectra

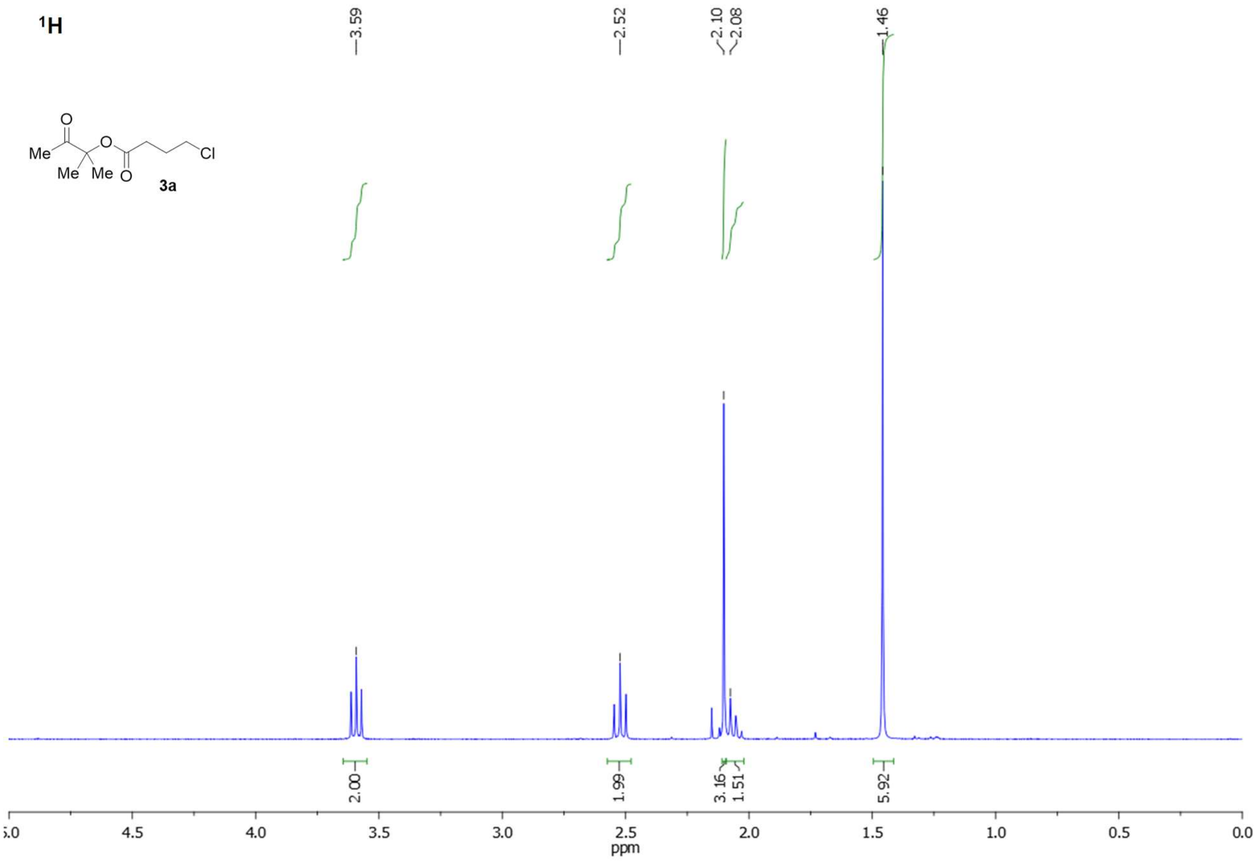
1H
3.59
2.52
2.10
2.08
1.46
Me
O
O
Me
Me
O
Cl
3a
2.00
1.99
3.16
1.51
5.92
ppm

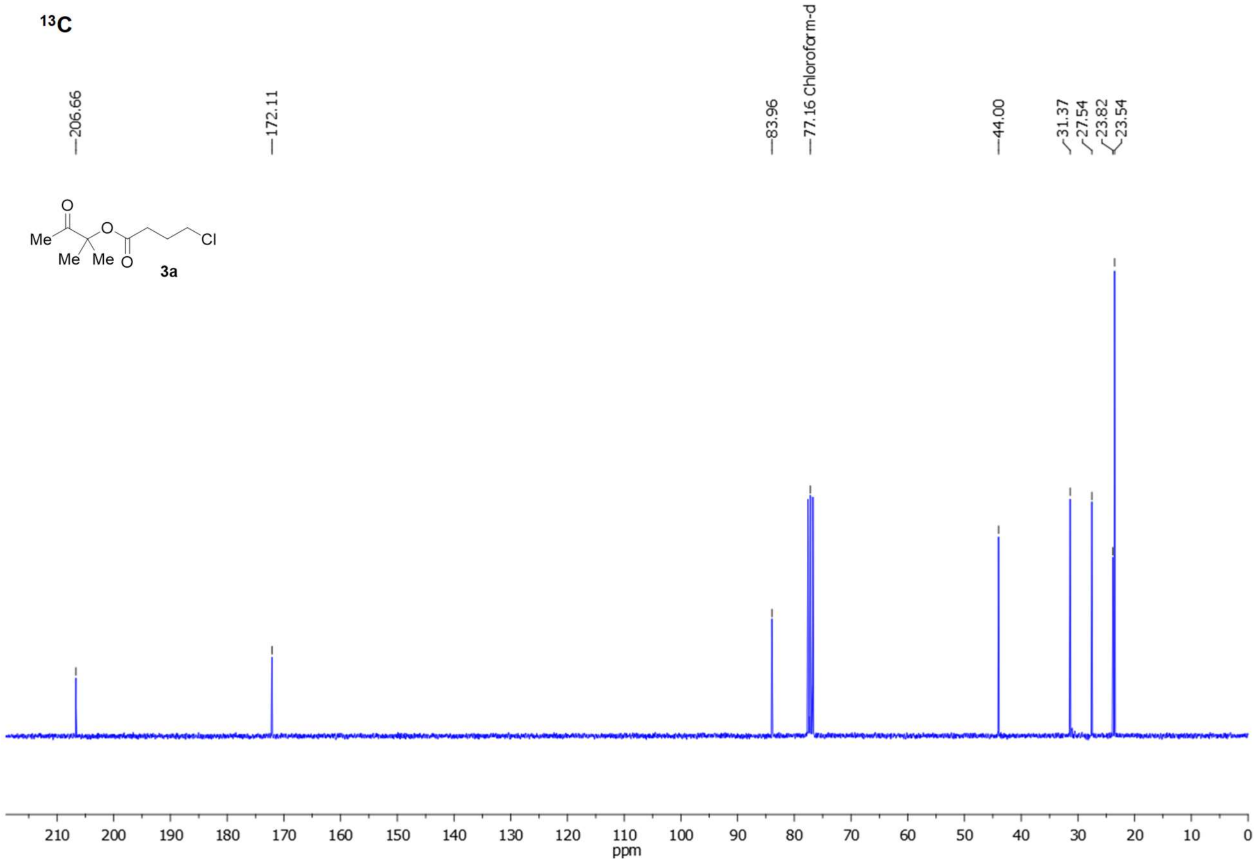
13C
206.66
172.11
83.96
77.16 Chloroform-d
44.00
31.37
27.54
23.82
23.54
Me
O
O
Me
Me
O
Cl
3a
ppm

DEPT

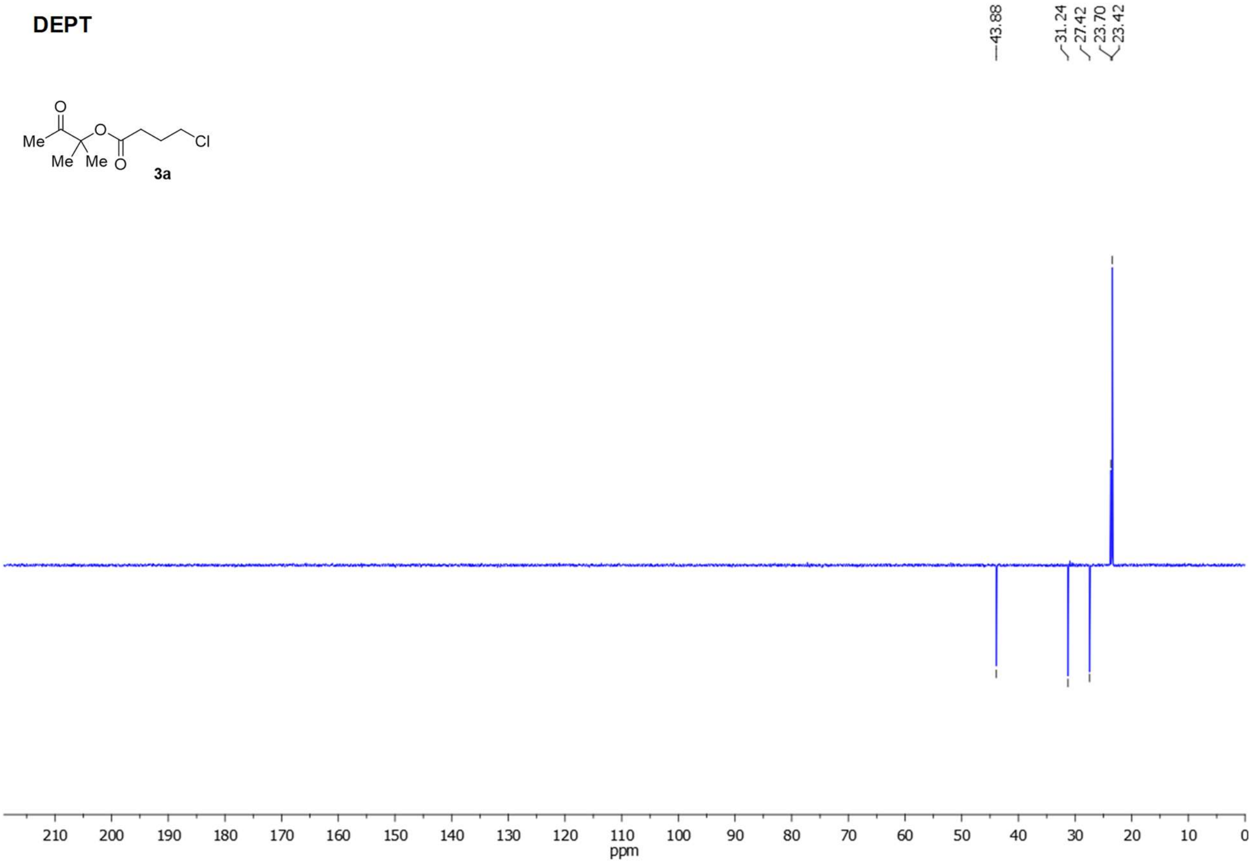
43.88
31.24
27.42
23.70
23.42
Me
Me
Me
O
O
O
Cl
3a
210
200
190
180
170
160
150
140
130
120
110
100
90
80
70
60
50
40
30
20
10
0
ppm

^{1}H

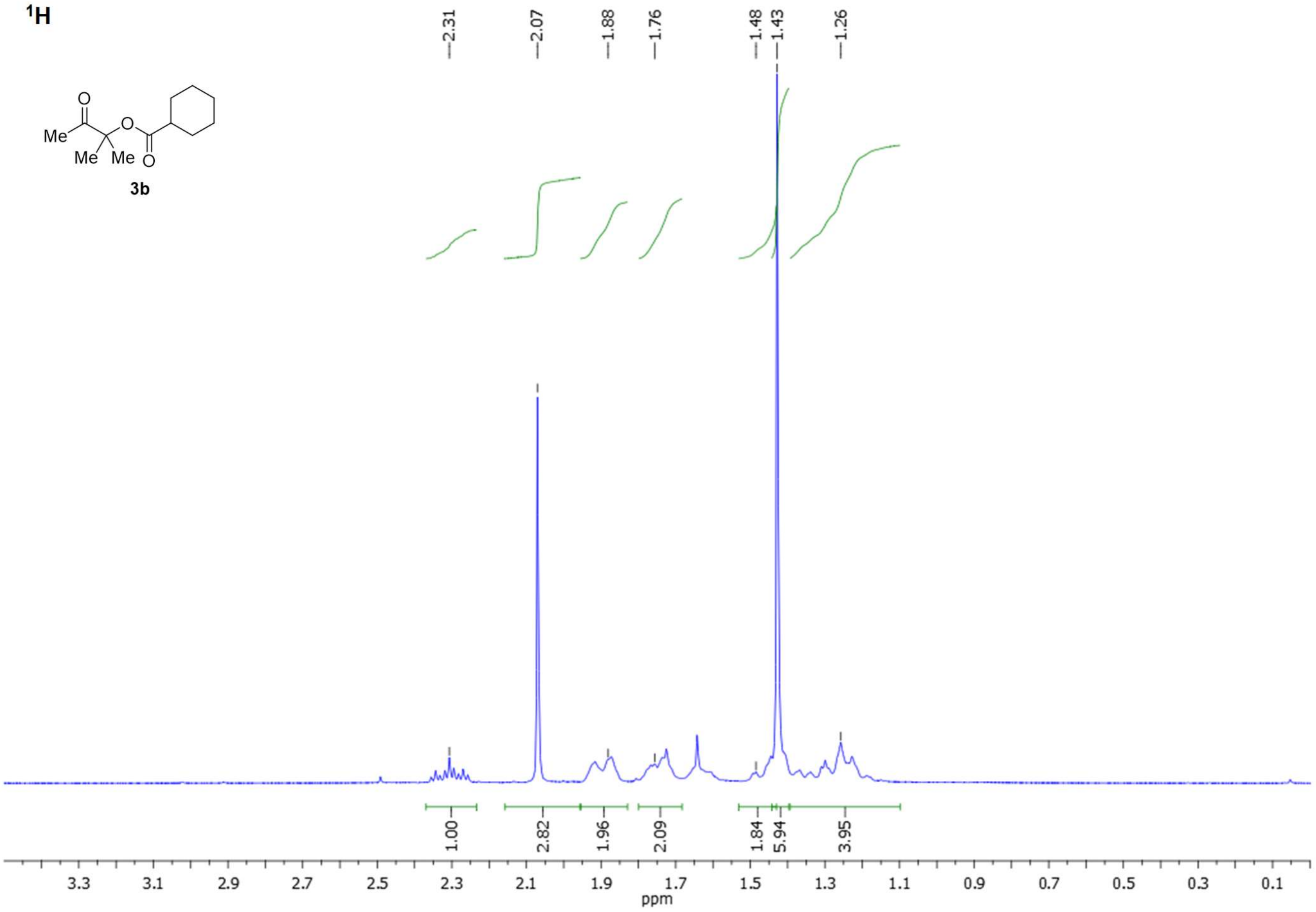
2.31
2.07
1.88
1.76
1.48
1.43
1.26
Me
Me
Me
O
O
O
3b
1.00
2.82
1.96
2.09
1.84
5.94
3.95
3.3
3.1
2.9
2.7
2.5
2.3
2.1
1.9
1.7
1.5
1.3
1.1
0.9
0.7
0.5
0.3
0.1
ppm

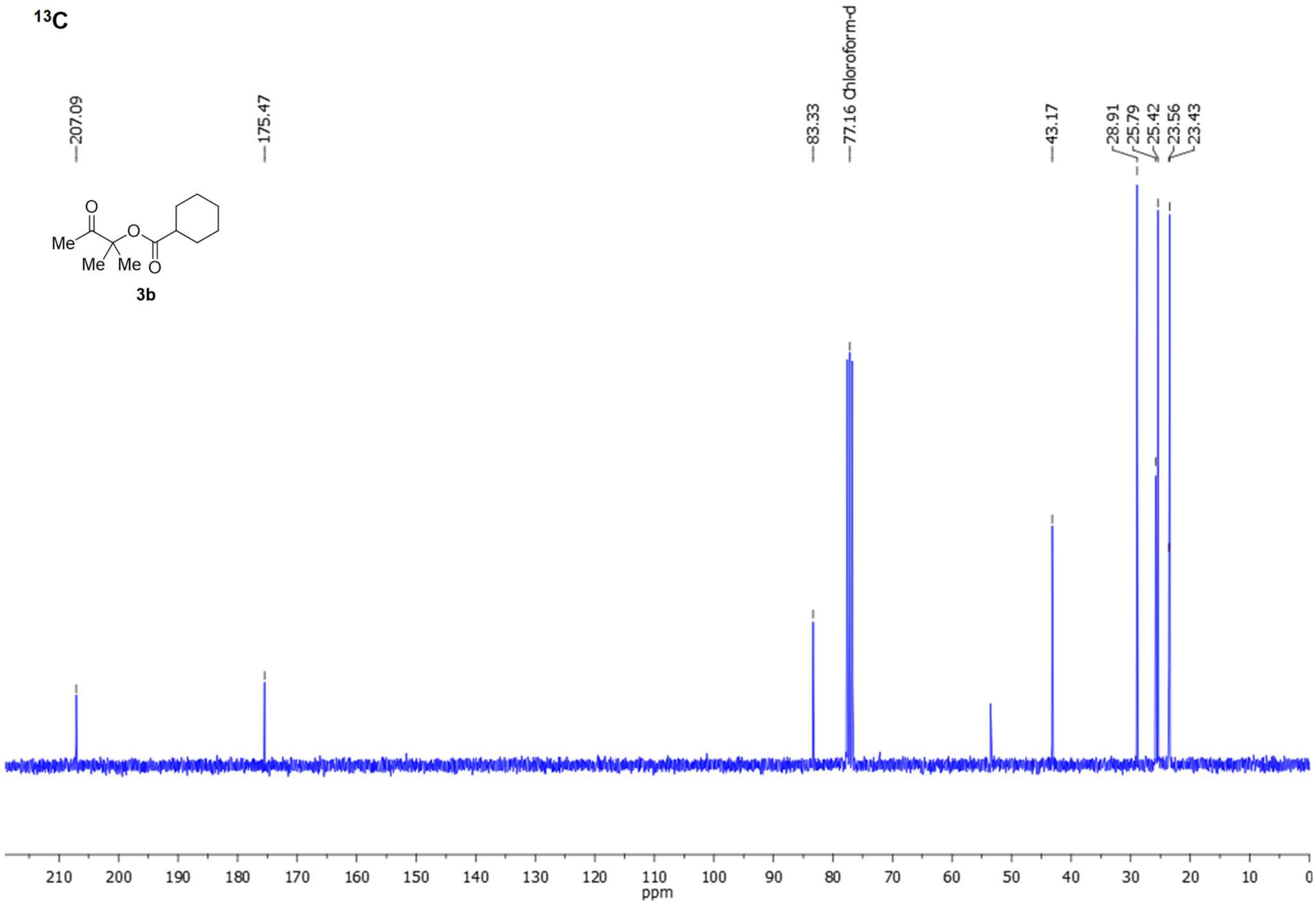
13C
207.09
175.47
83.33
77.16 Chloroform-d
43.17
28.91
25.79
25.42
23.56
23.43
Me
O
O
Me
Me
O
3b
210 200 190 180 170 160 150 140 130 120 110 100 90 80 70 60 50 40 30 20 10 0
ppm

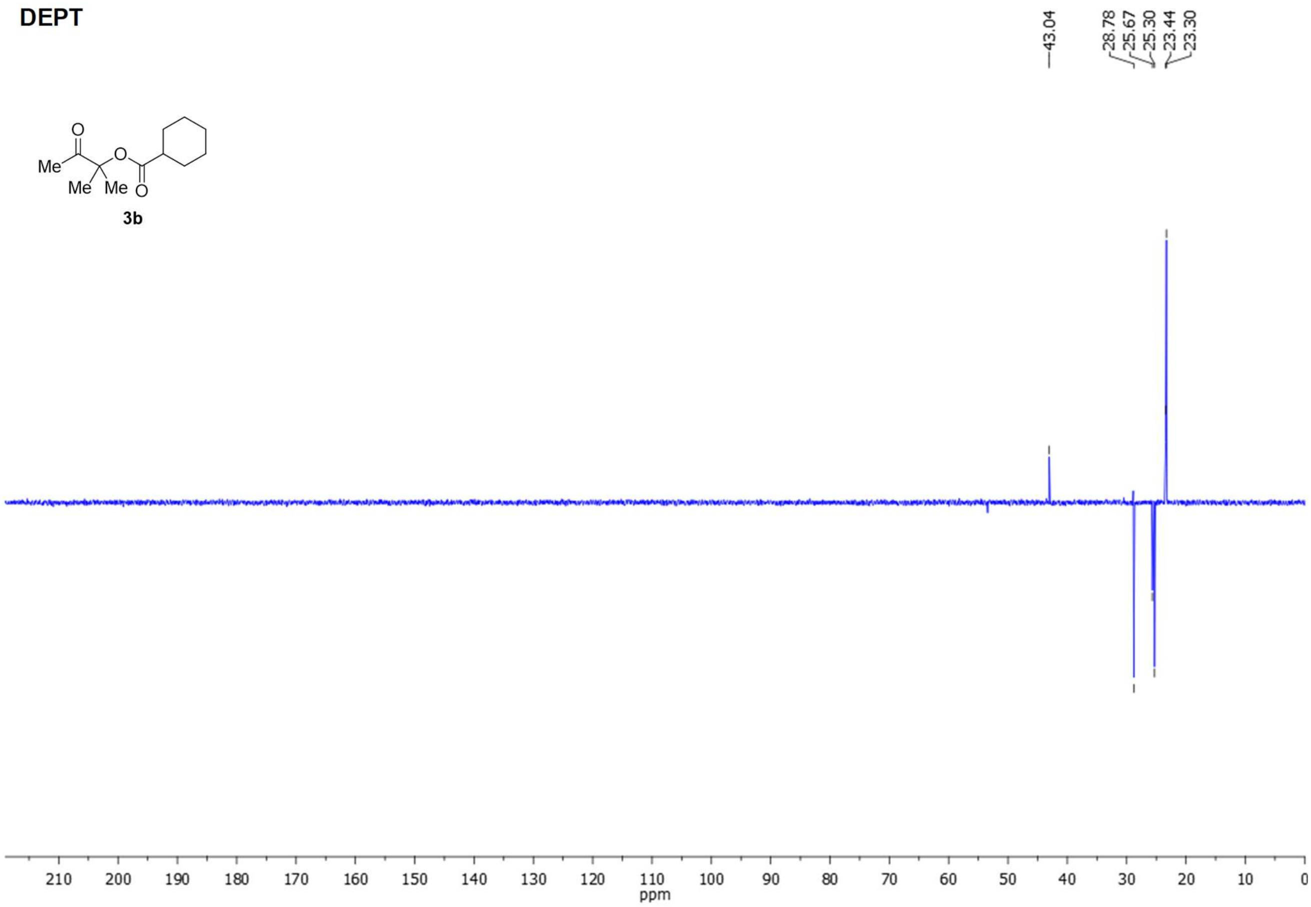
DEPT
43.04
28.78
25.67
25.30
23.44
23.30
Me
O
O
Me
Me
O
3b
210 200 190 180 170 160 150 140 130 120 110 100 90 80 70 60 50 40 30 20 10 0
ppm

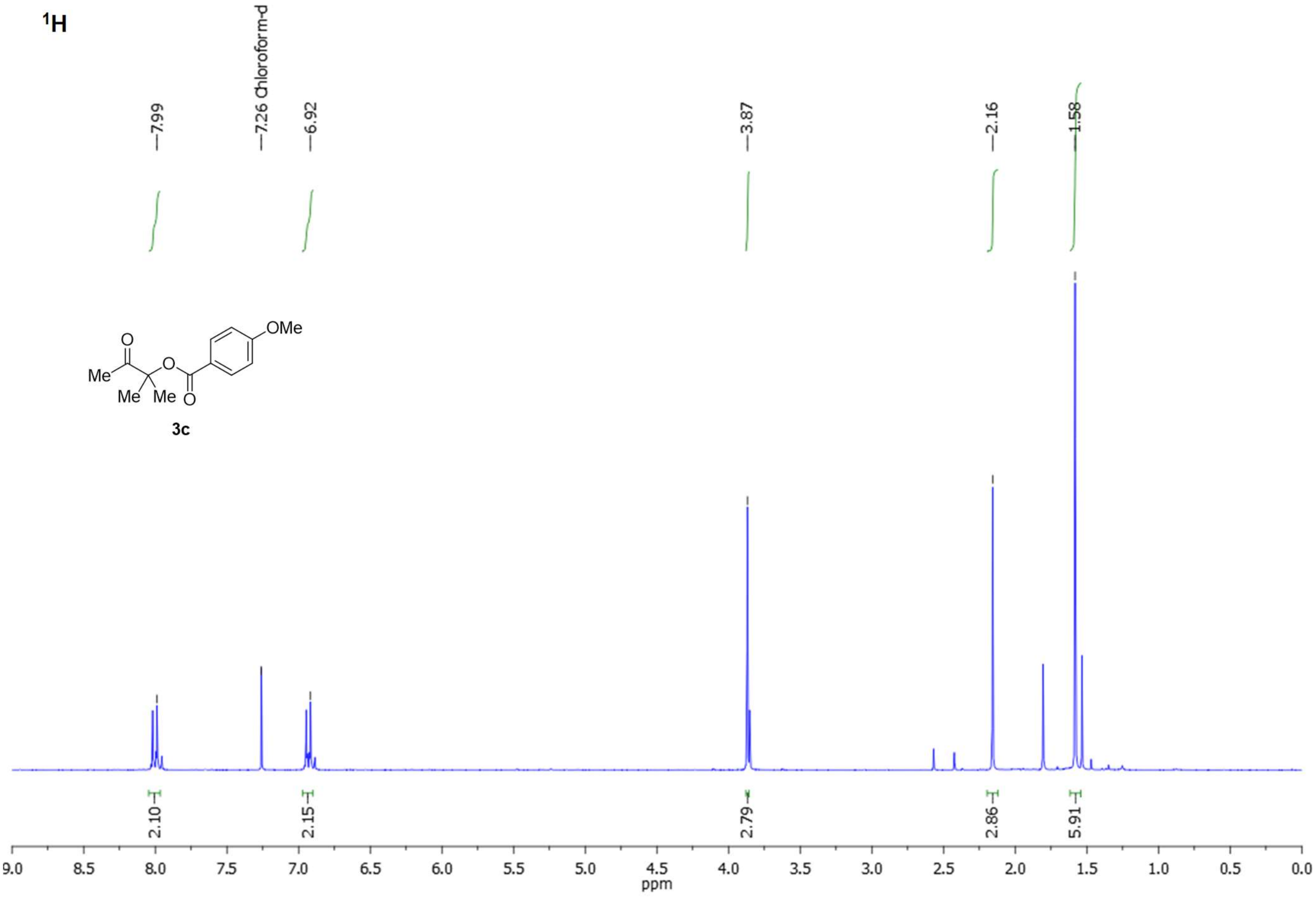
1H
7.99
7.26 Chloroform-d
6.92
3.87
2.16
1.58
OMe
Me
Me
Me
O
O
O
3c
2.10
2.15
2.79
2.86
5.91
ppm

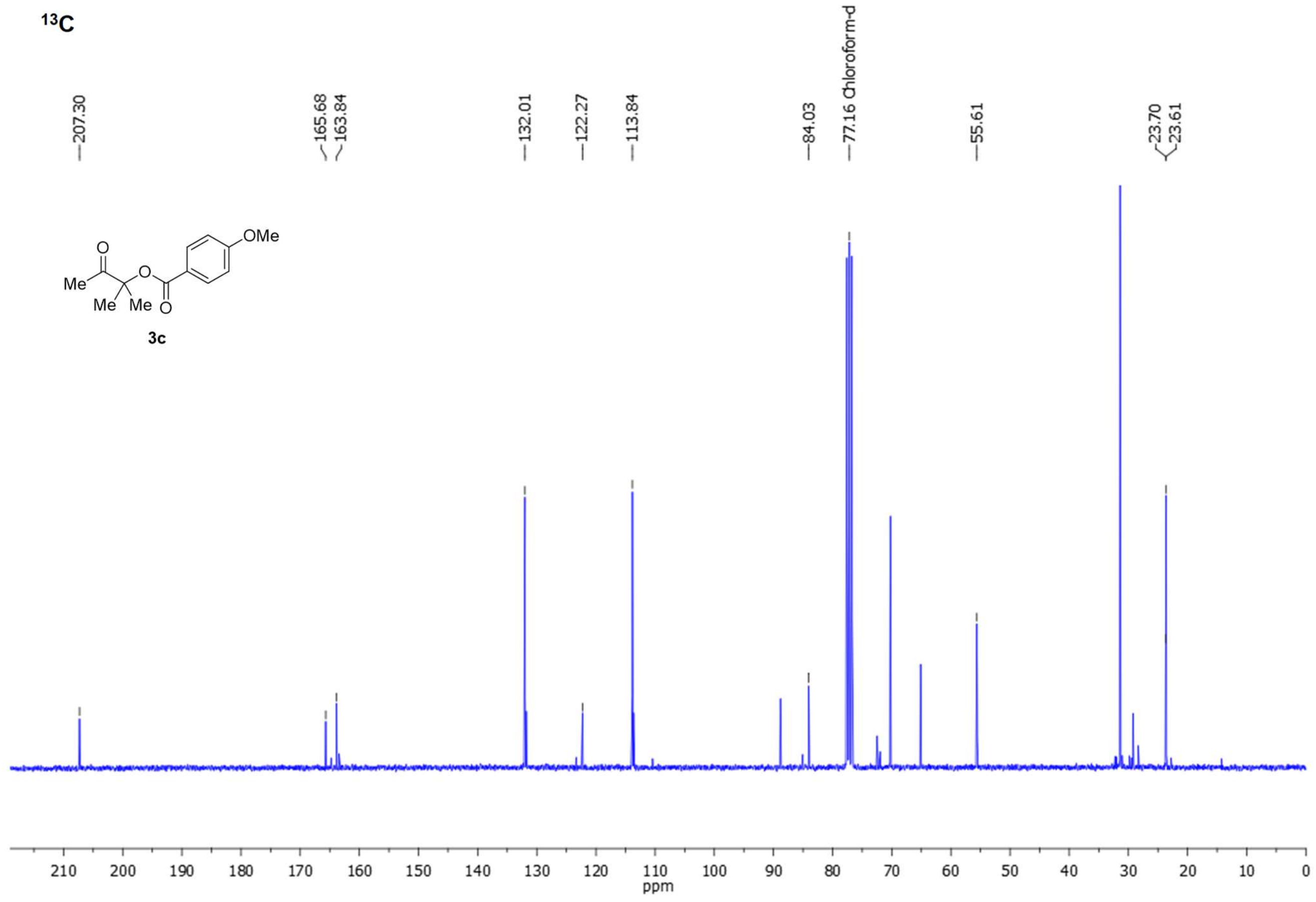
13C
207.30
165.68
163.84
132.01
122.27
113.84
84.03
77.16 Chloroform-d
55.61
23.70
23.61
OMe
Me
Me
Me
O
O
O
3c
ppm

DEPT

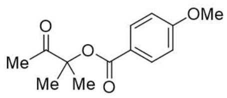

3c

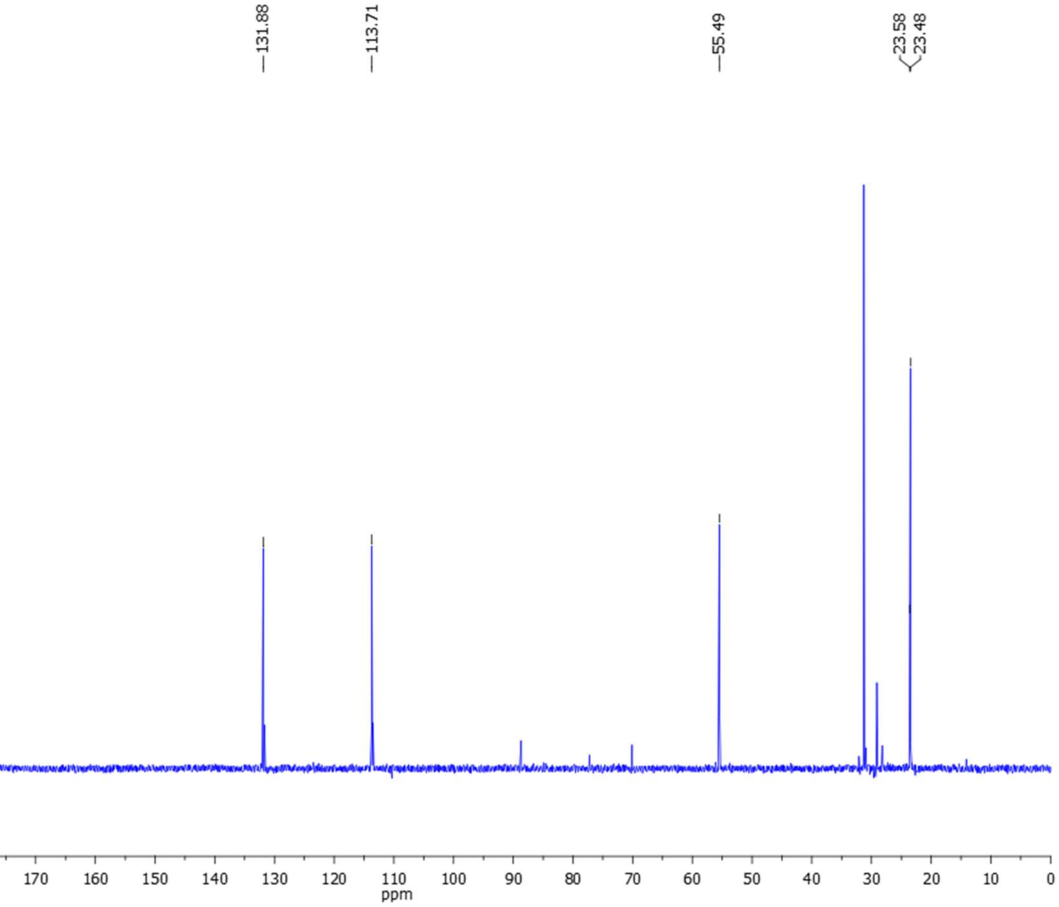
131.88
113.71
55.49
23.58
23.48
210
200
190
180
170
160
150
140
130
120
110
100
90
80
70
60
50
40
30
20
10
0
ppm

^{1}H

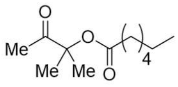

3d

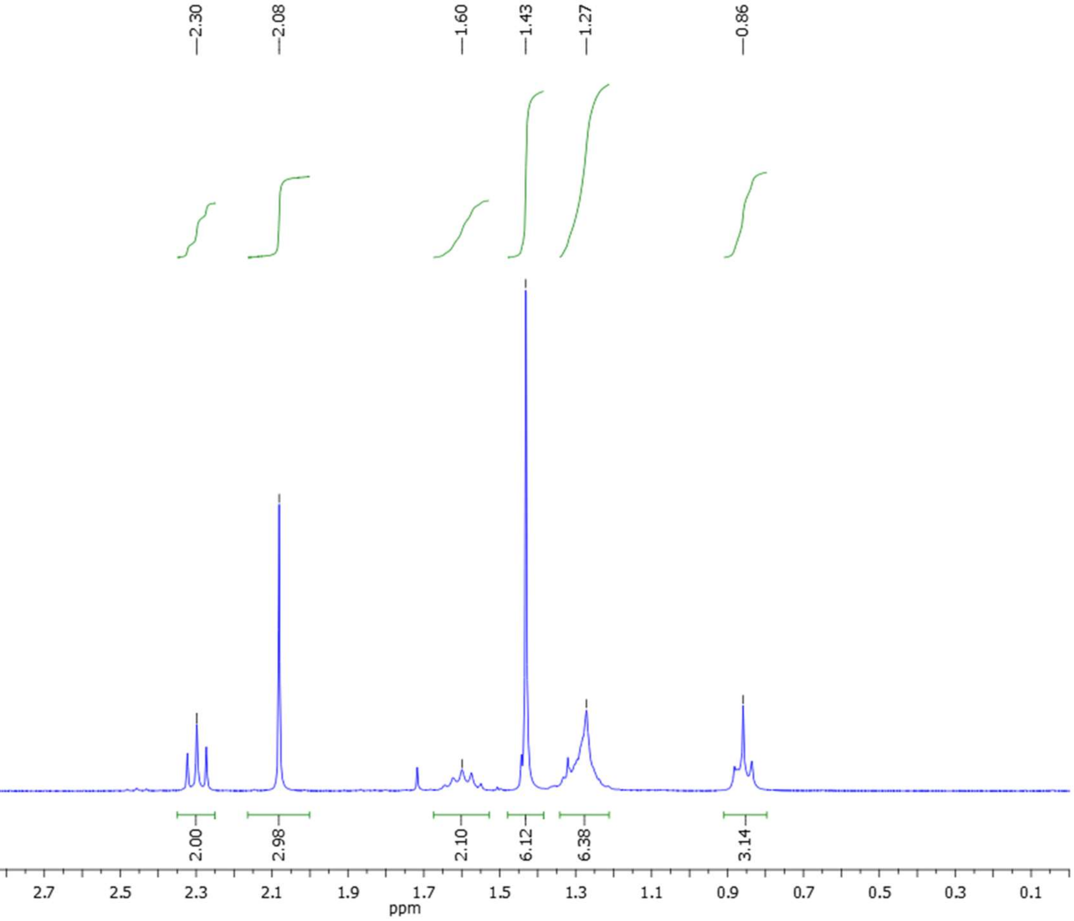
2.30
2.08
1.60
1.43
1.27
0.86
2.00
2.98
2.10
6.12
6.38
3.14
3.3
3.1
2.9
2.7
2.5
2.3
2.1
1.9
1.7
1.5
1.3
1.1
0.9
0.7
0.5
0.3
0.1
ppm

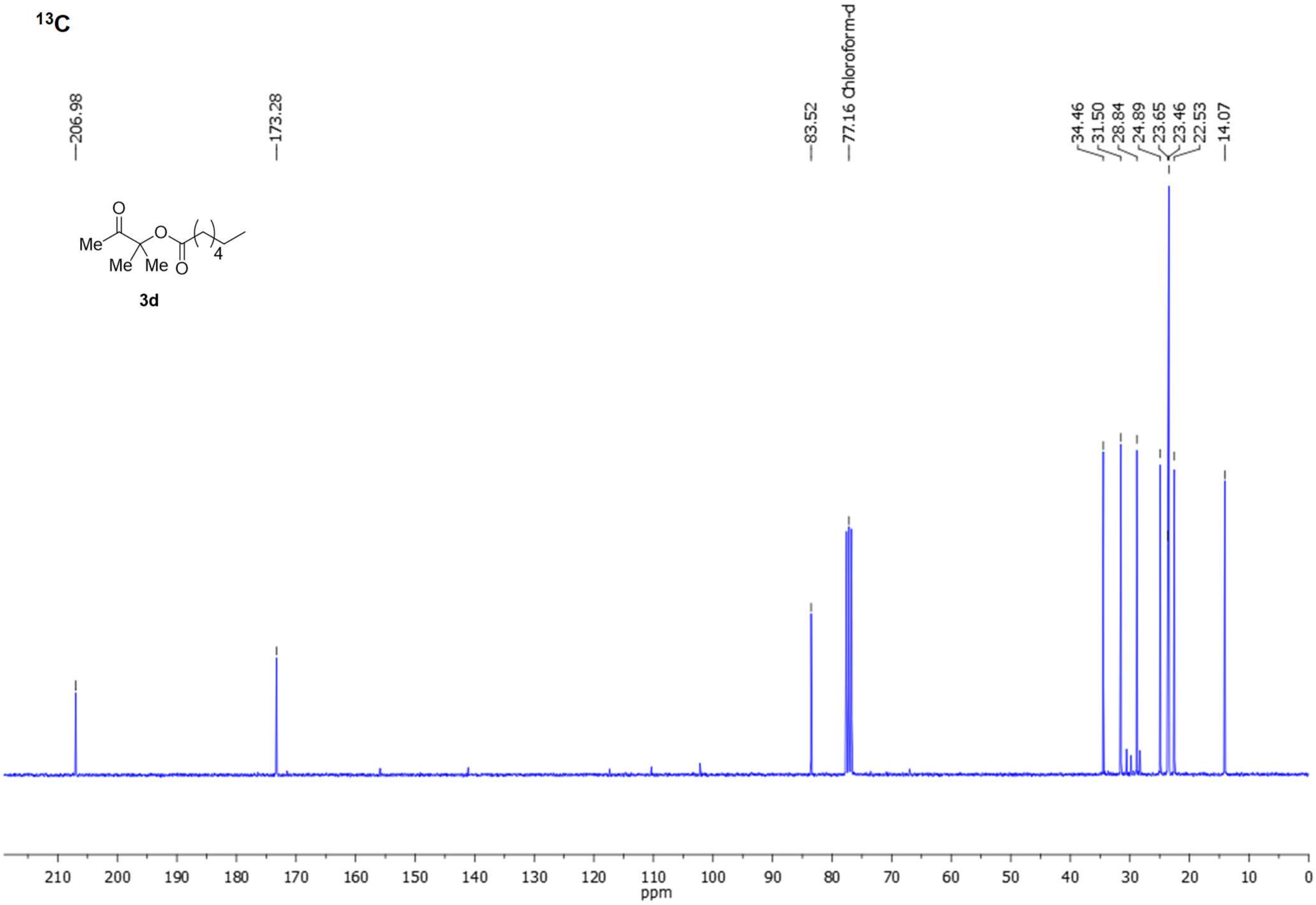
13C
206.98
173.28
83.52
77.16 Chloroform-d
34.46
31.50
28.84
24.89
23.65
23.46
22.53
14.07
Me
O
O
Me
Me
O
4
3d
ppm

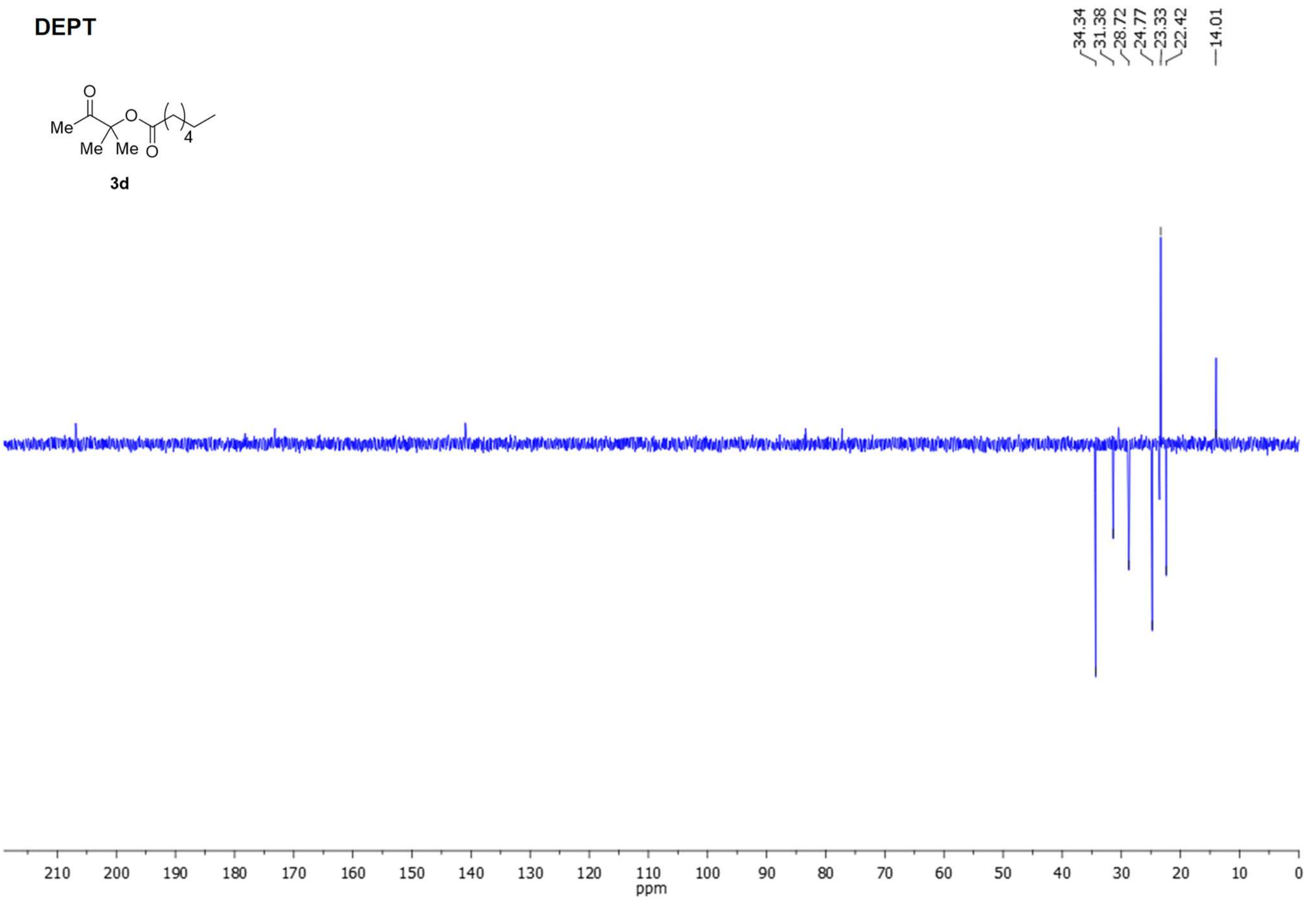
DEPT
34.34
31.38
28.72
24.77
23.33
22.42
14.01
Me
O
O
Me
Me
O
4
3d
ppm

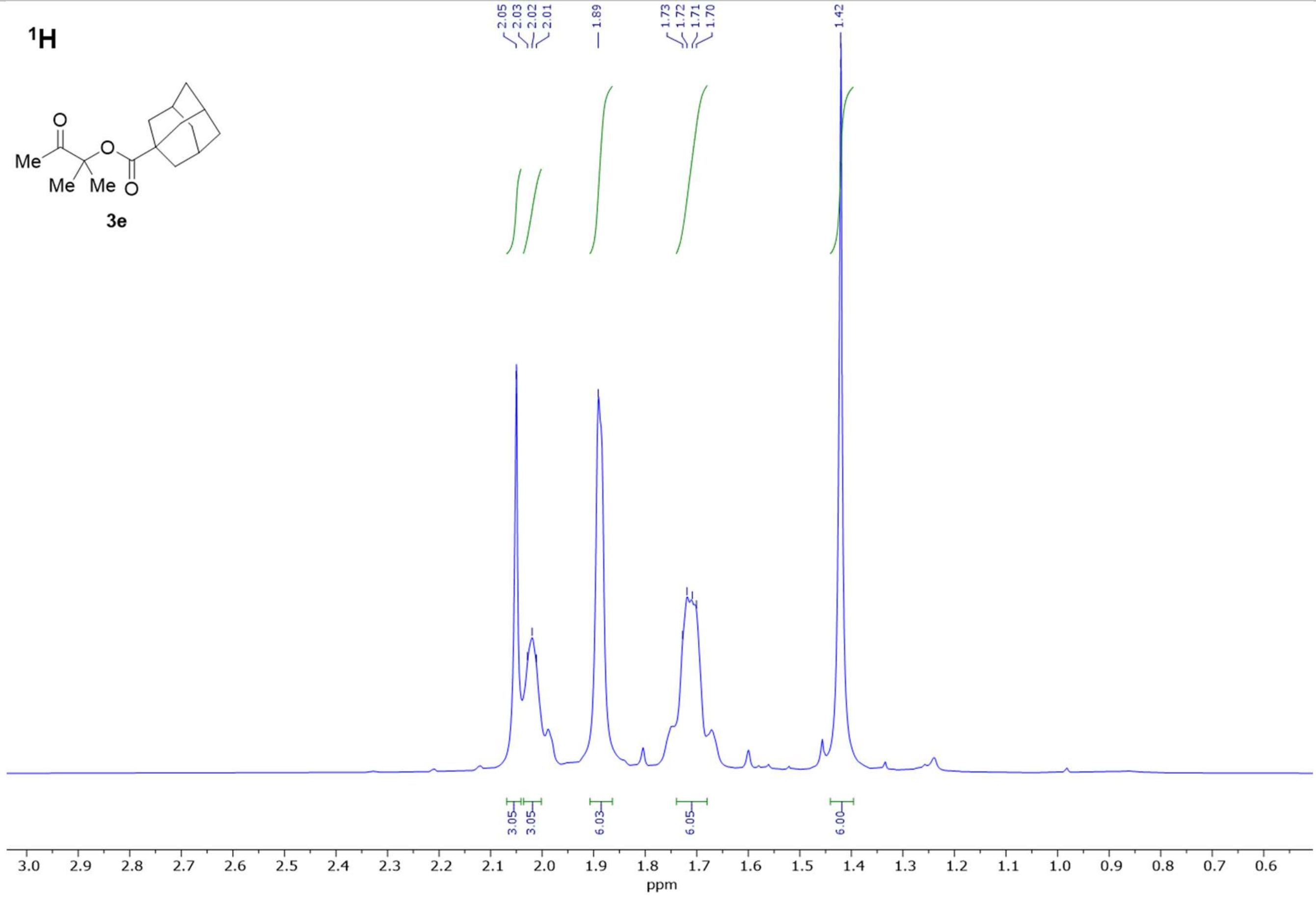
1H
Me
Me Me O
3e
2.05
2.03
2.02
2.01
1.89
1.73
1.72
1.71
1.70
1.42
3.05
3.05
6.03
6.05
6.00
ppm

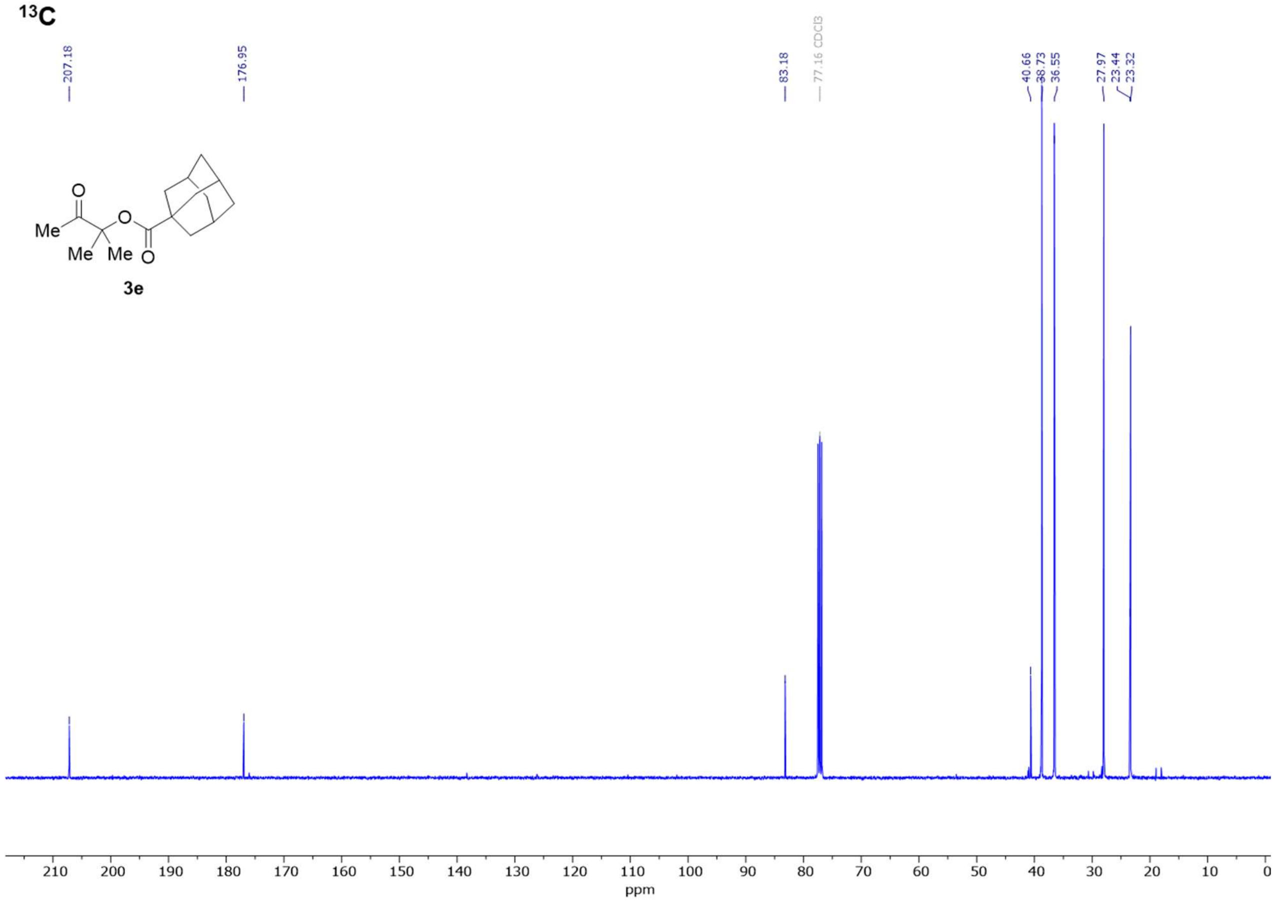
13C
207.18
176.95
83.18
77.16 CDCl3
40.66
38.73
36.55
27.97
23.44
23.32
Me
Me Me O
3e
ppm

DEPT

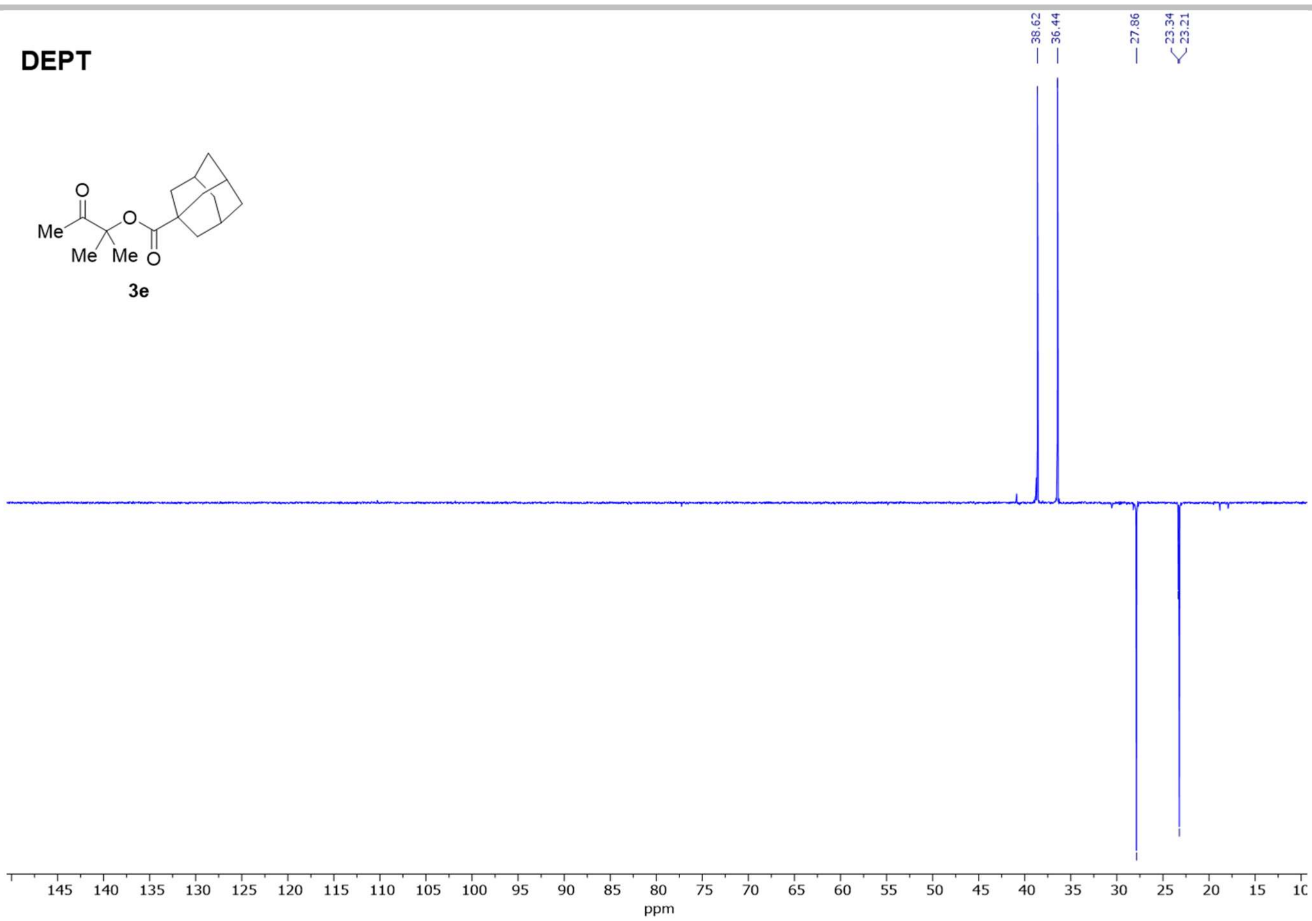
Me
Me
Me
O
O
O
3e
38.62
36.44
27.86
23.34
23.21
145
140
135
130
125
120
115
110
105
100
95
90
85
80
75
70
65
60
55
50
45
40
35
30
25
20
15
10
ppm

[1]H

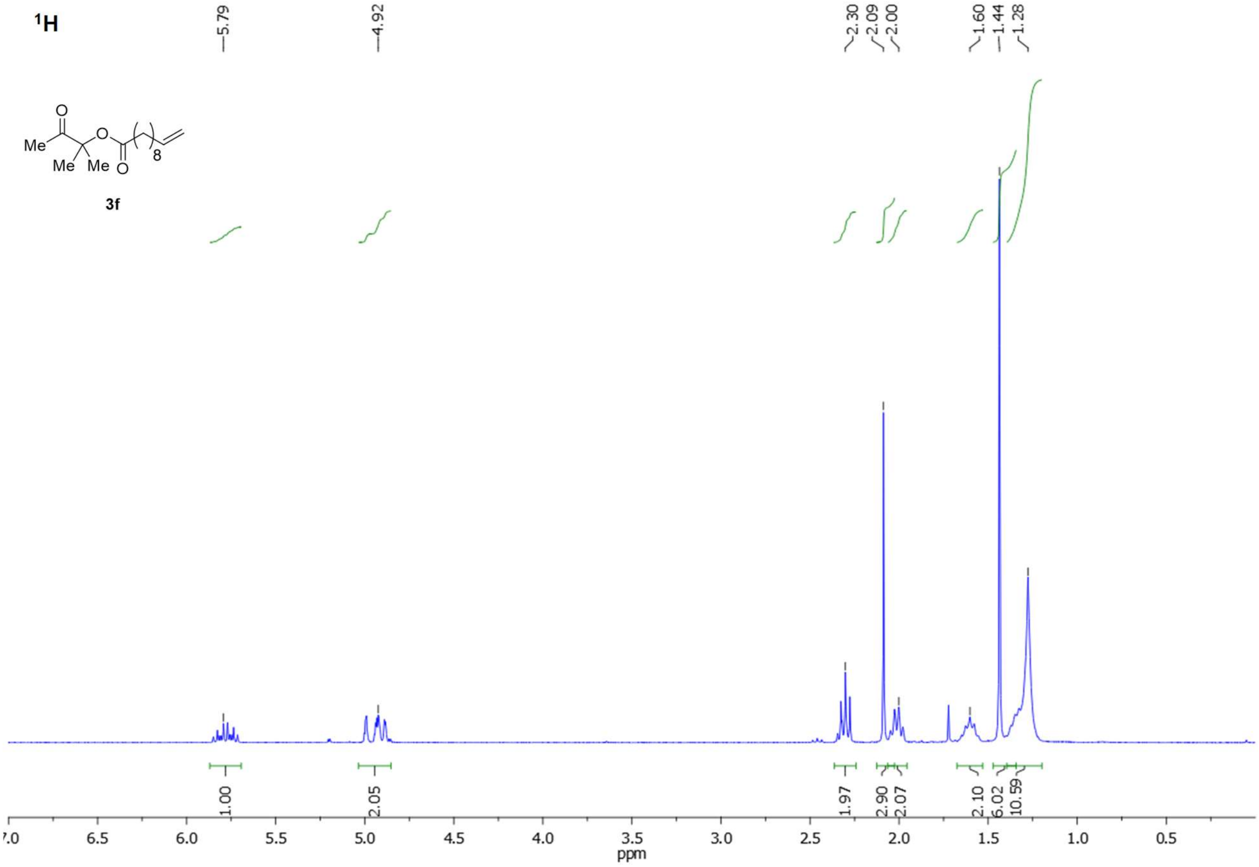
Me
Me
Me
O
O
O
8
3f
5.79
4.92
2.30
2.09
2.00
1.60
1.44
1.28
1.00
2.05
1.97
2.90
2.07
2.10
6.02
10.59
6.5
6.0
5.5
5.0
4.5
4.0
3.5
3.0
2.5
2.0
1.5
1.0
0.5
ppm

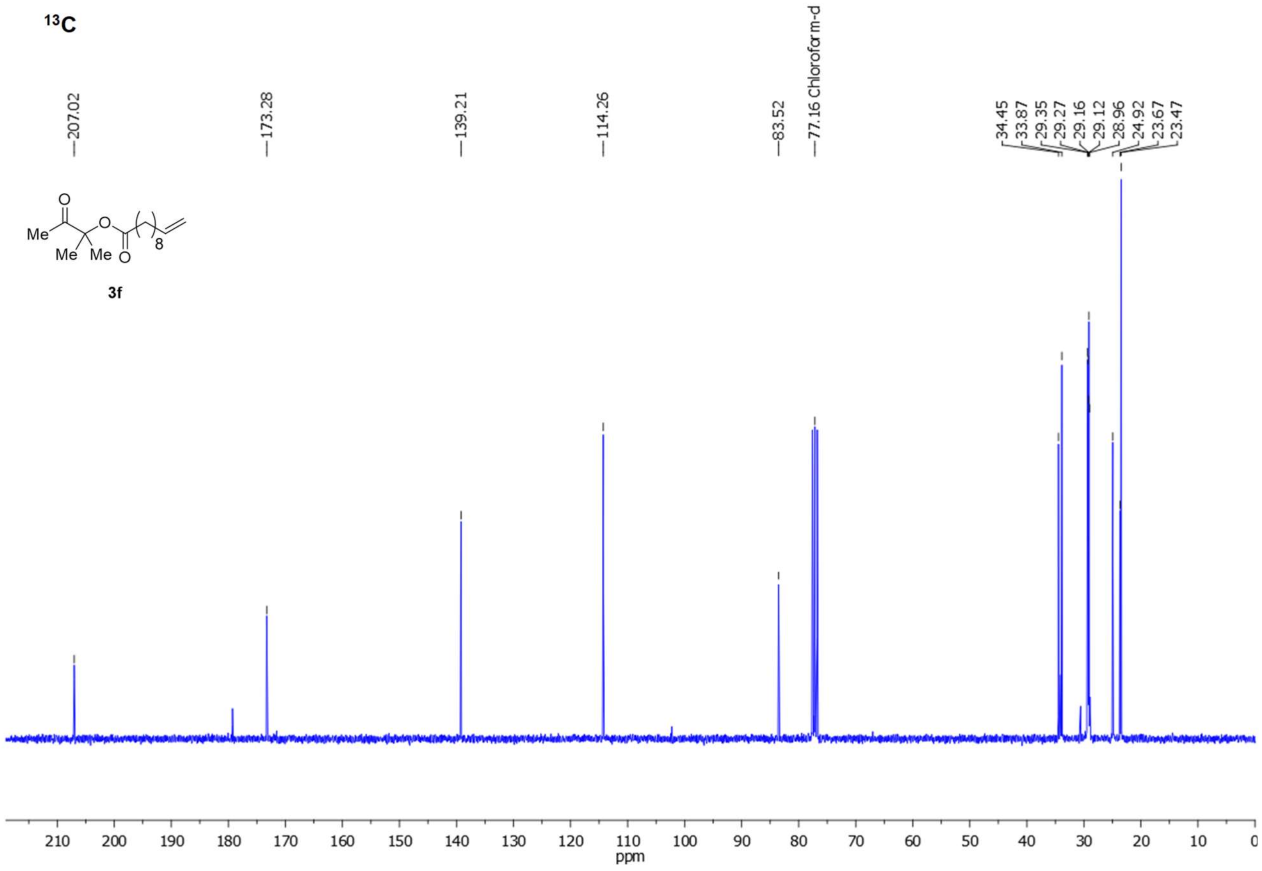
13C
207.02
173.28
139.21
114.26
83.52
77.16 Chloroform-d
34.45
33.87
29.35
29.27
29.16
29.12
28.96
24.92
23.67
23.47
Me
O
Me
Me
O
8
3f
210
200
190
180
170
160
150
140
130
120
110
100
90
80
70
60
50
40
30
20
10
0
ppm

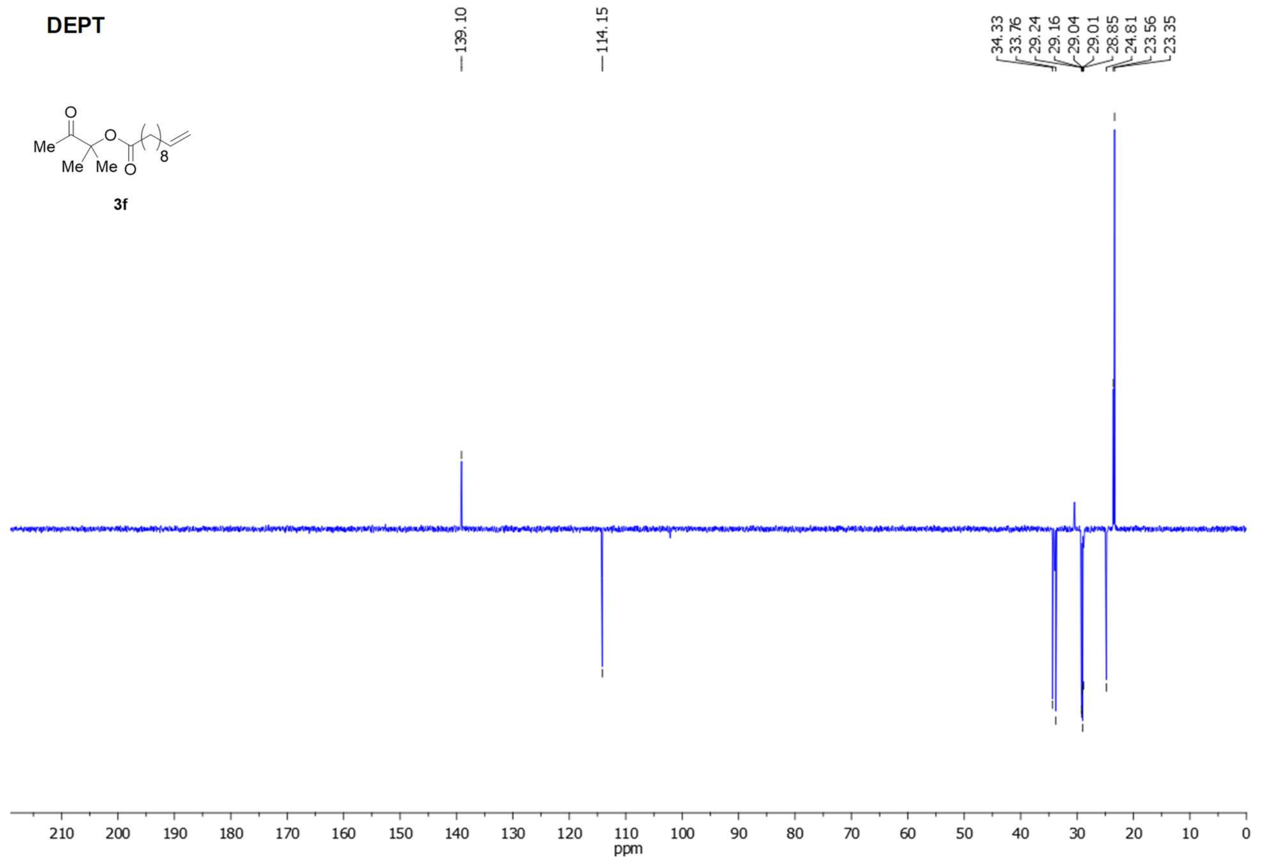
DEPT
139.10
114.15
34.33
33.76
29.24
29.16
29.04
29.01
28.85
24.81
23.56
23.35
Me
O
Me
Me
O
8
3f
210
200
190
180
170
160
150
140
130
120
110
100
90
80
70
60
50
40
30
20
10
0
ppm

^1H

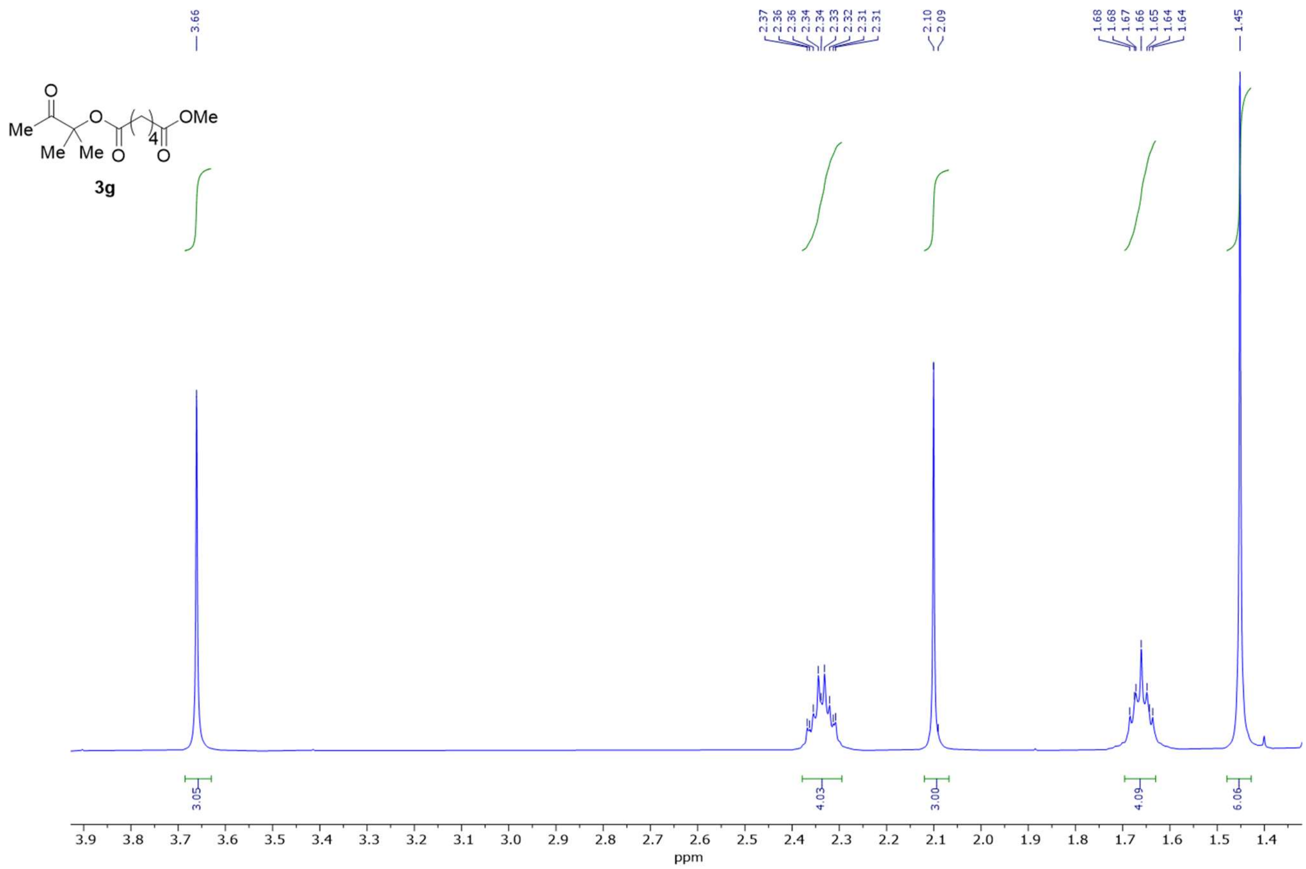
3.66
2.37
2.36
2.36
2.34
2.34
2.33
2.32
2.31
2.31
2.10
2.09
1.68
1.68
1.67
1.66
1.65
1.64
1.64
1.45
Me
O
O
Me
Me
O
4
OMe
O
3g
3.05
4.03
3.00
4.09
6.06
3.9
3.8
3.7
3.6
3.5
3.4
3.3
3.2
3.1
3.0
2.9
2.8
2.7
2.6
2.5
2.4
2.3
2.2
2.1
2.0
1.9
1.8
1.7
1.6
1.5
1.4
ppm

^{13}C

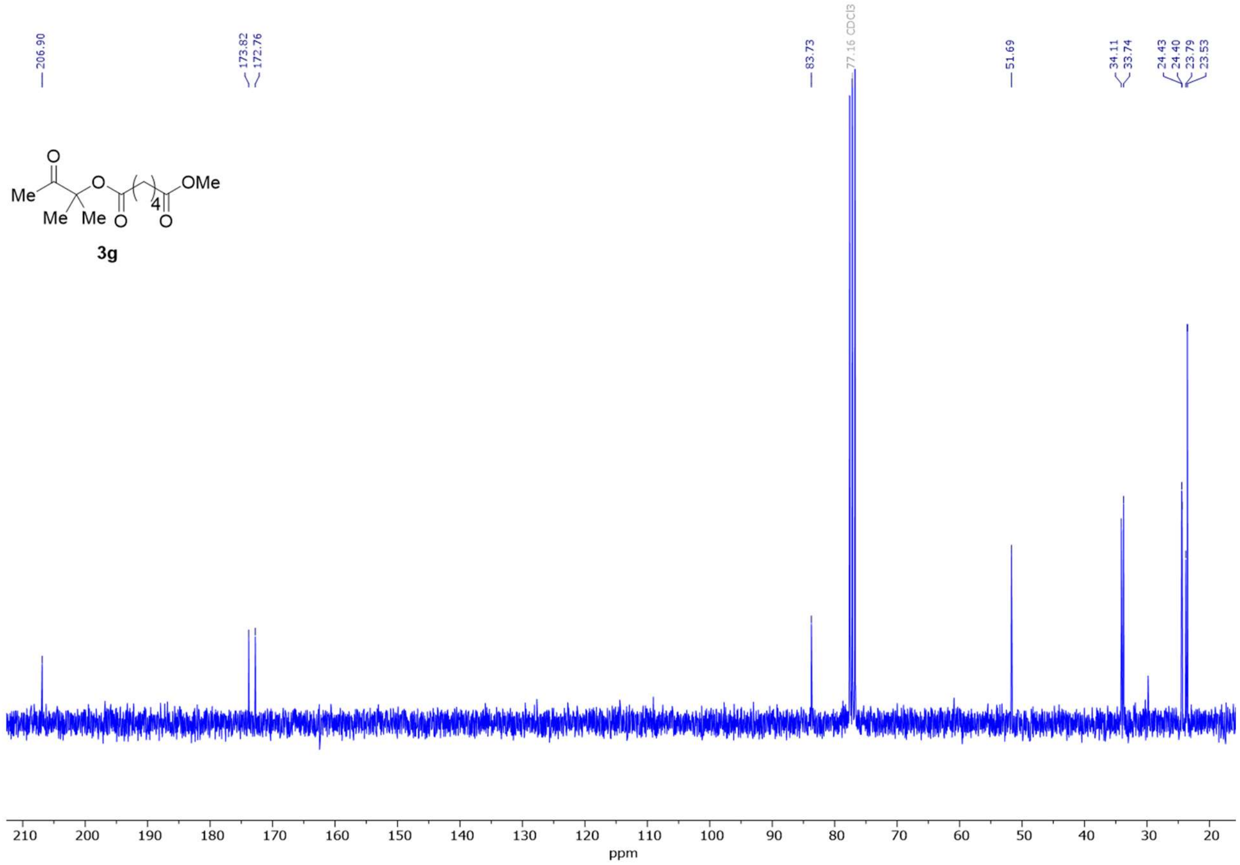
206.90
173.82
172.76
83.73
77.16 CDCl3
51.69
34.11
33.74
24.43
24.40
23.79
23.53
Me
O
O
Me
Me
O
4
OMe
O
3g
210
200
190
180
170
160
150
140
130
120
110
100
90
80
70
60
50
40
30
20
ppm

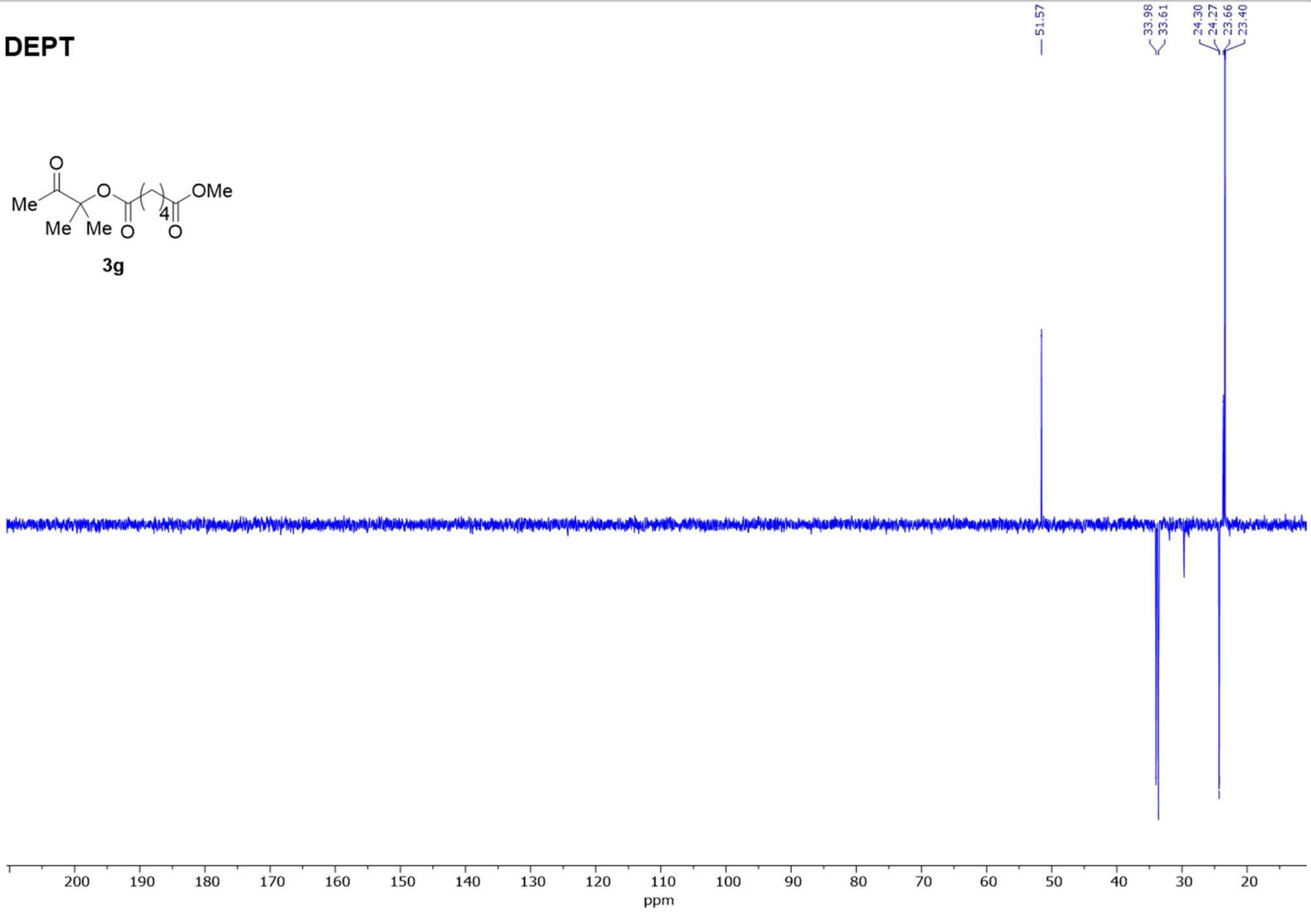
DEPT
51.57
33.98
33.61
24.30
24.27
23.66
23.40
O
Me
O
OMe
4
Me Me O
O
3g
200 190 180 170 160 150 140 130 120 110 100 90 80 70 60 50 40 30 20
ppm

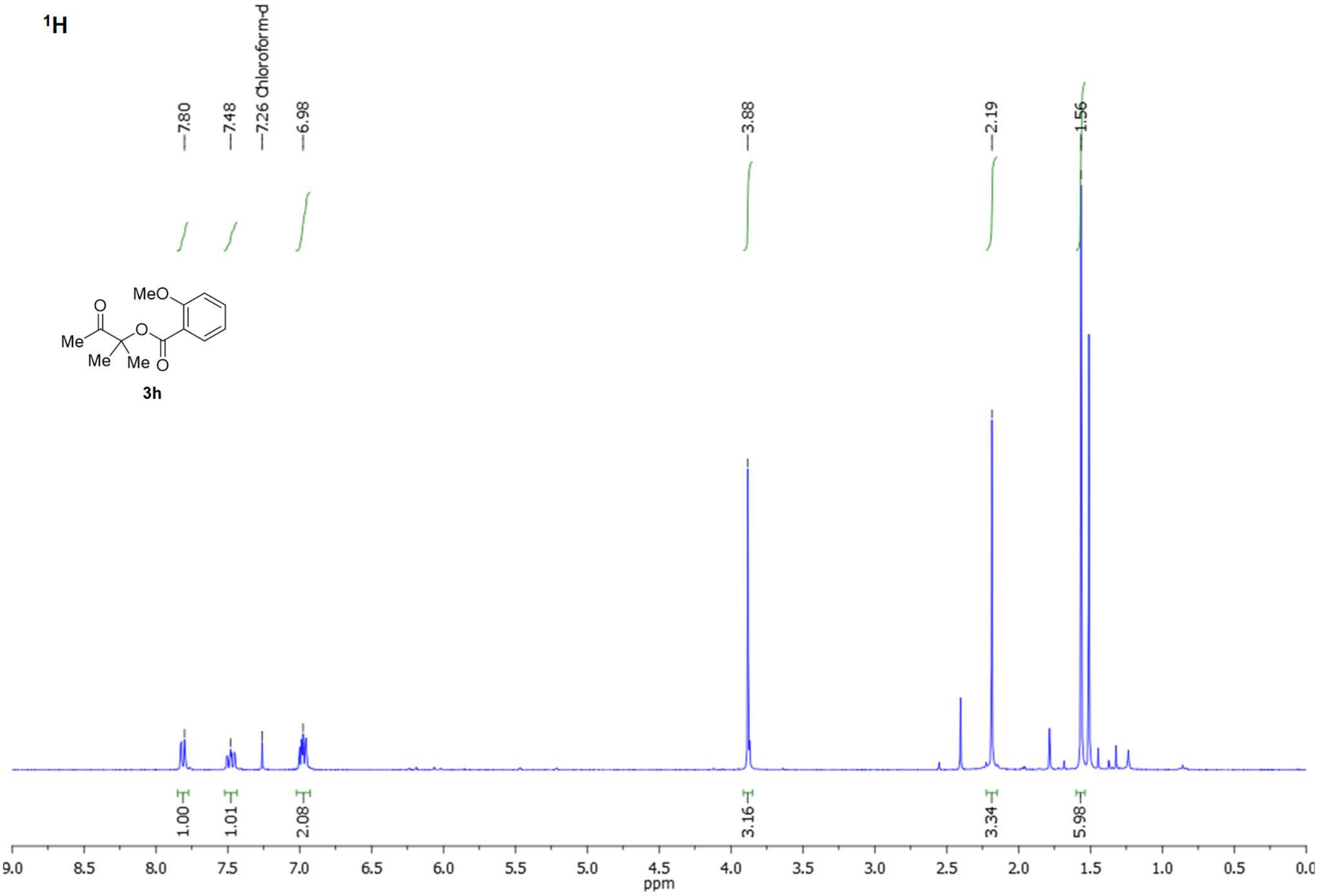
1H
7.80
7.48
7.26 Chloroform-d
6.98
3.88
2.19
1.56
MeO
O
Me
O
Me Me O
3h
1.00
1.01
2.08
3.16
3.34
5.98
9.0 8.5 8.0 7.5 7.0 6.5 6.0 5.5 5.0 4.5 4.0 3.5 3.0 2.5 2.0 1.5 1.0 0.5 0.0
ppm

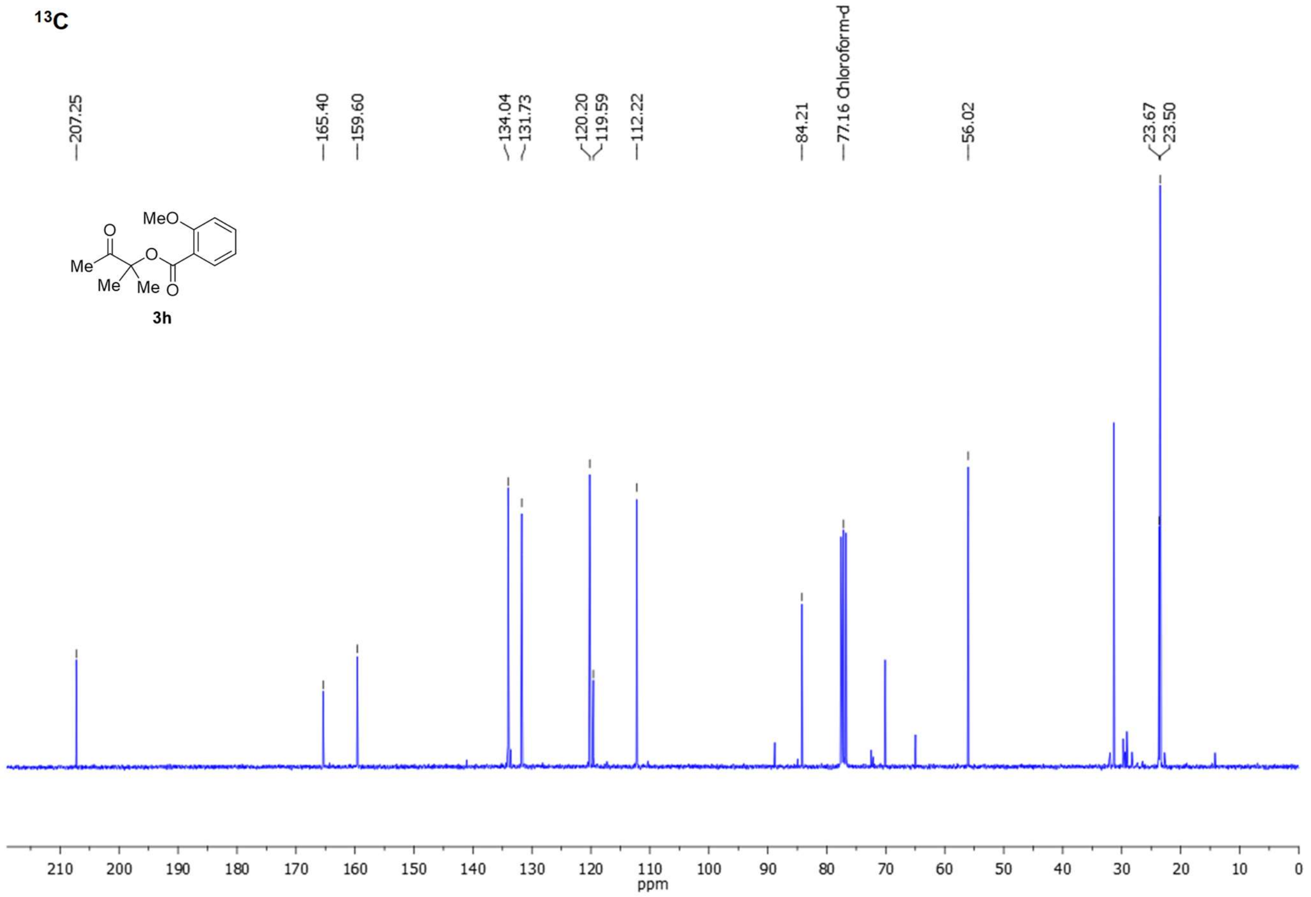
13C
207.25
165.40
159.60
134.04
131.73
120.20
119.59
112.22
84.21
77.16 Chloroform-d
56.02
23.67
23.50
MeO
O
Me
O
Me
Me
O
3h
210 200 190 180 170 160 150 140 130 120 110 100 90 80 70 60 50 40 30 20 10 0
ppm

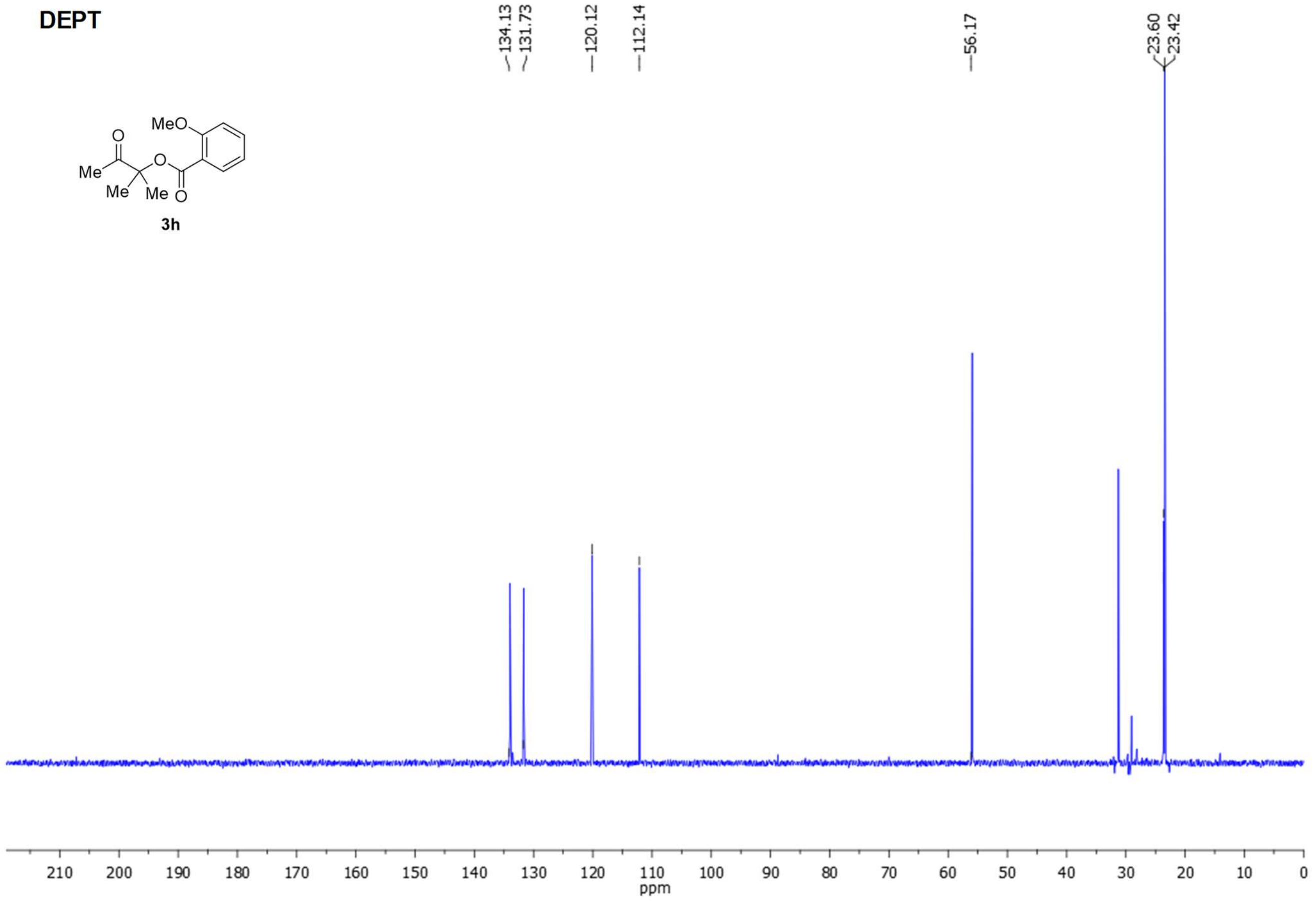
DEPT
134.13
131.73
120.12
112.14
56.17
23.60
23.42
MeO
O
Me
O
Me
Me
O
3h
210 200 190 180 170 160 150 140 130 120 110 100 90 80 70 60 50 40 30 20 10 0
ppm

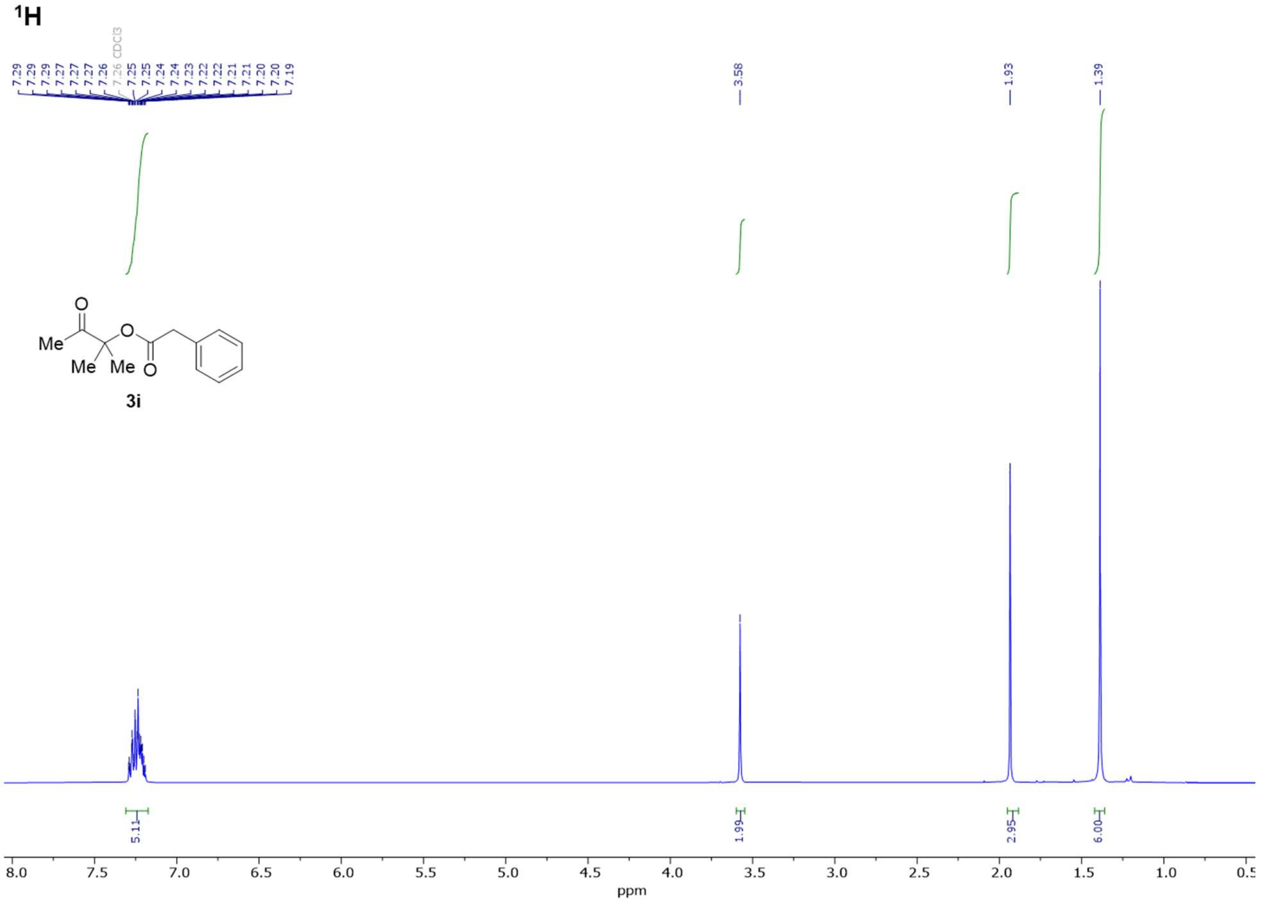
1H
7.29 7.29 7.29 7.27 7.27 7.27 7.26 7.26 CDCl3 7.25 7.25 7.24 7.24 7.23 7.22 7.22 7.21 7.21 7.20 7.20 7.19
3.58
1.93
1.39
Me
Me
Me
O
O
O
3i
5.11
1.99
2.95
6.00
ppm

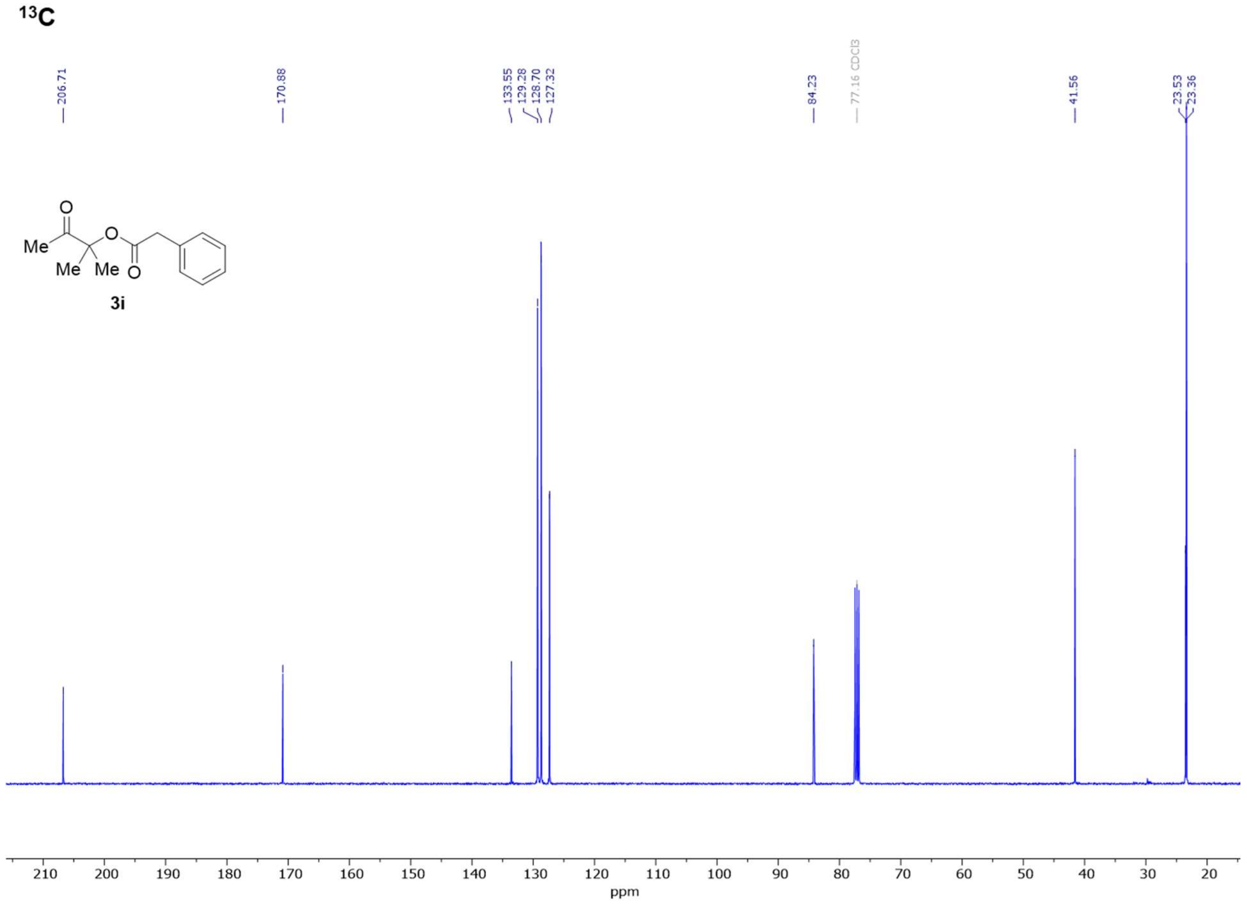
13C
206.71
170.88
133.55
129.28
128.70
127.32
84.23
77.16 CDCl3
41.56
23.53
23.36
Me
Me
Me
O
O
O
3i
ppm

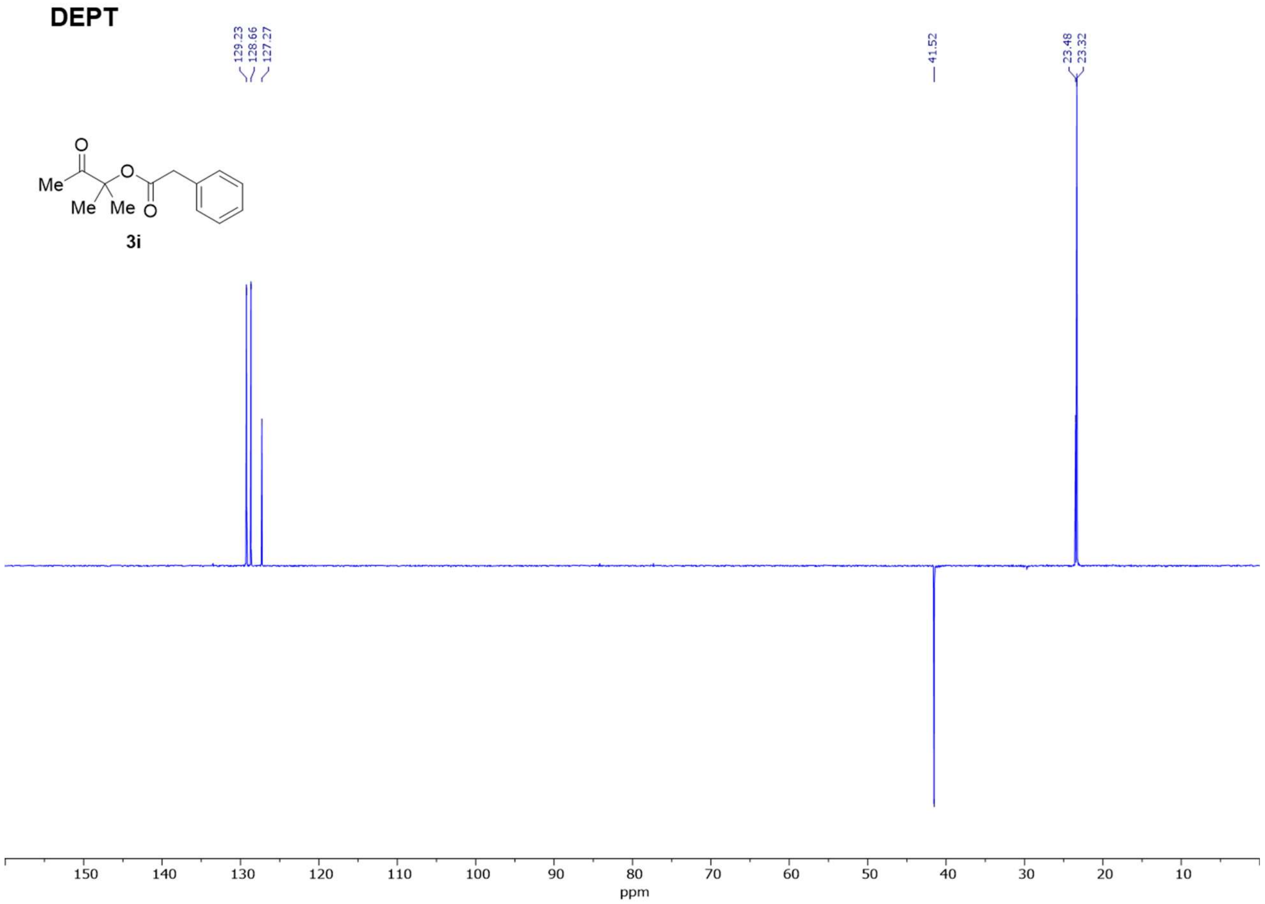
DEPT
129.23
128.66
127.27
41.52
23.48
23.32
Me
Me
Me
O
O
O
3i
150
140
130
120
110
100
90
80
70
60
50
40
30
20
10
ppm

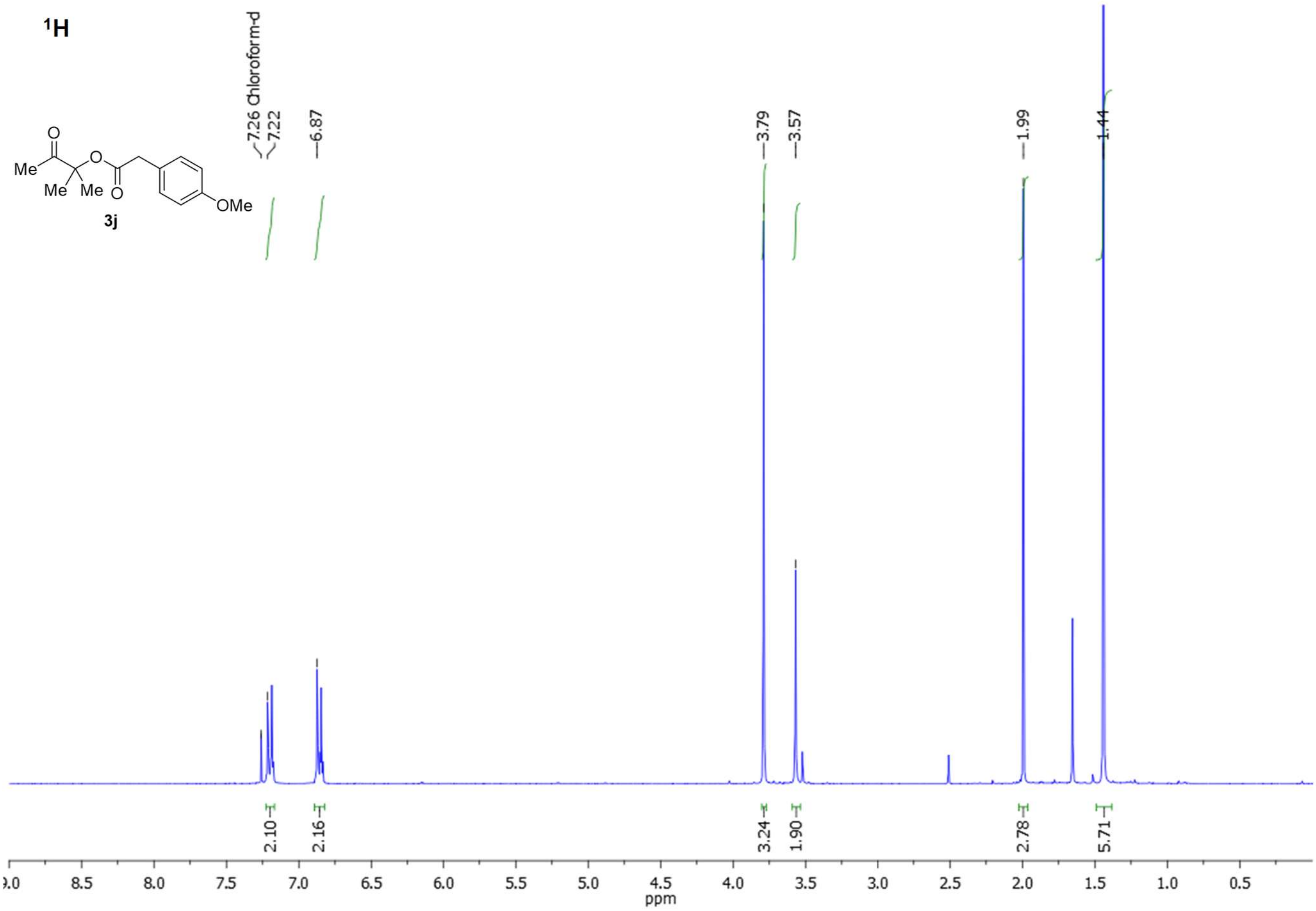
1H
7.26 Chloroform-d
7.22
6.87
3.79
3.57
1.99
1.44
Me
Me
Me
O
O
O
OMe
3j
2.10
2.16
3.24
1.90
2.78
5.71
8.5
8.0
7.5
7.0
6.5
6.0
5.5
5.0
4.5
4.0
3.5
3.0
2.5
2.0
1.5
1.0
0.5
ppm

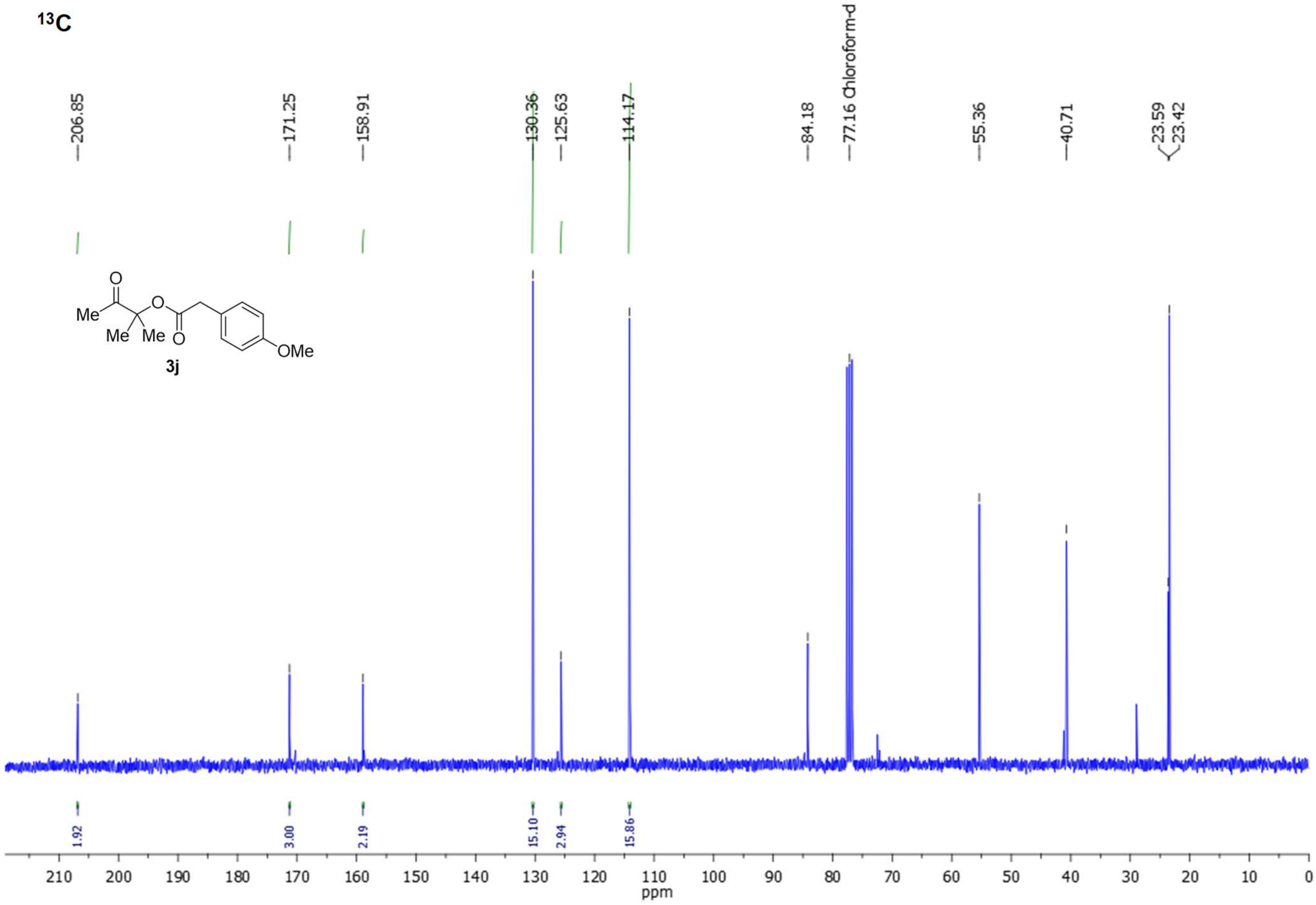
13C
206.85
171.25
158.91
130.36
125.63
114.17
84.18
77.16 Chloroform-d
55.36
40.71
23.59
23.42
Me
O
O
Me Me O
OMe
3j
1.92
3.00
2.19
15.10
2.94
15.86
210 200 190 180 170 160 150 140 130 120 110 100 90 80 70 60 50 40 30 20 10 0
ppm

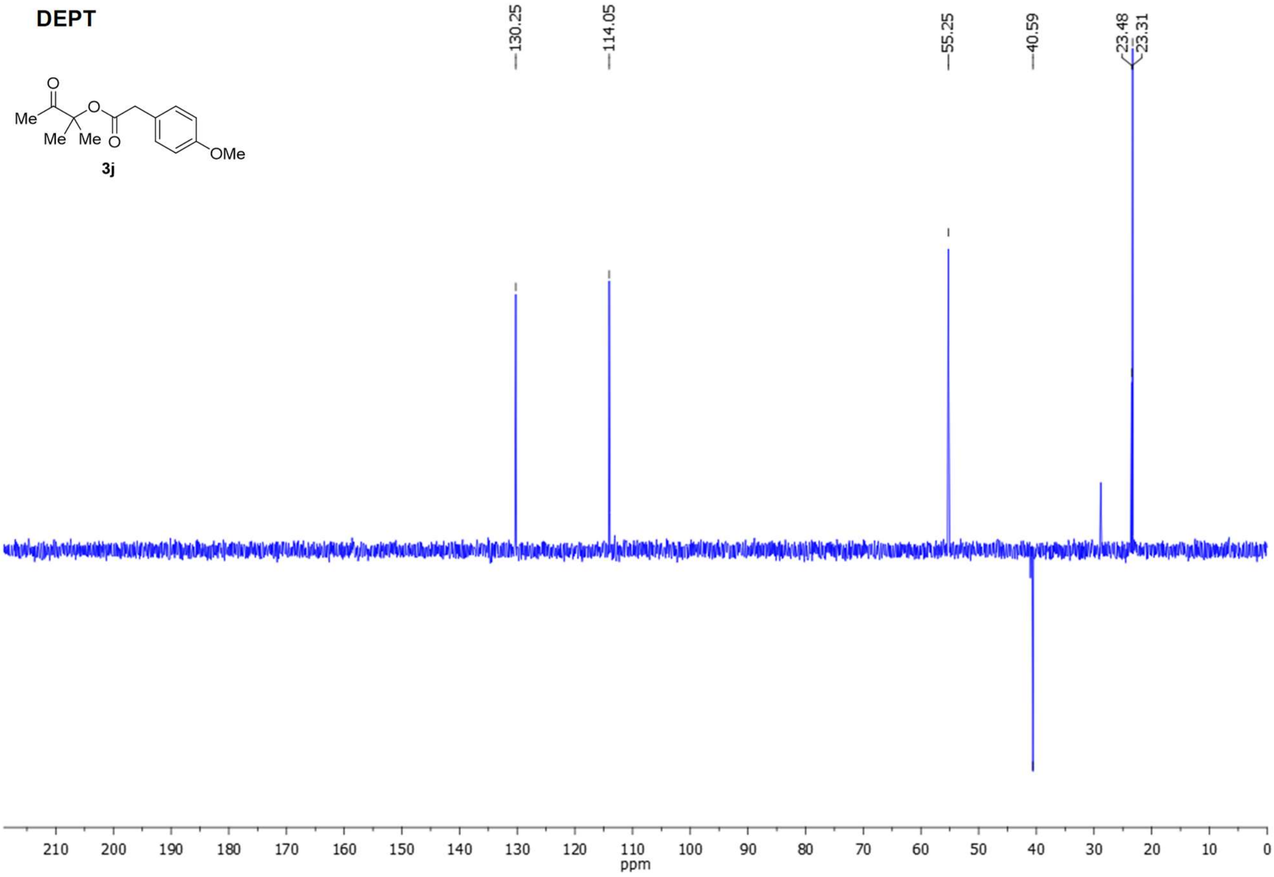
DEPT
130.25
114.05
55.25
40.59
23.48
23.31
Me
O
O
Me Me O
OMe
3j
210 200 190 180 170 160 150 140 130 120 110 100 90 80 70 60 50 40 30 20 10 0
ppm

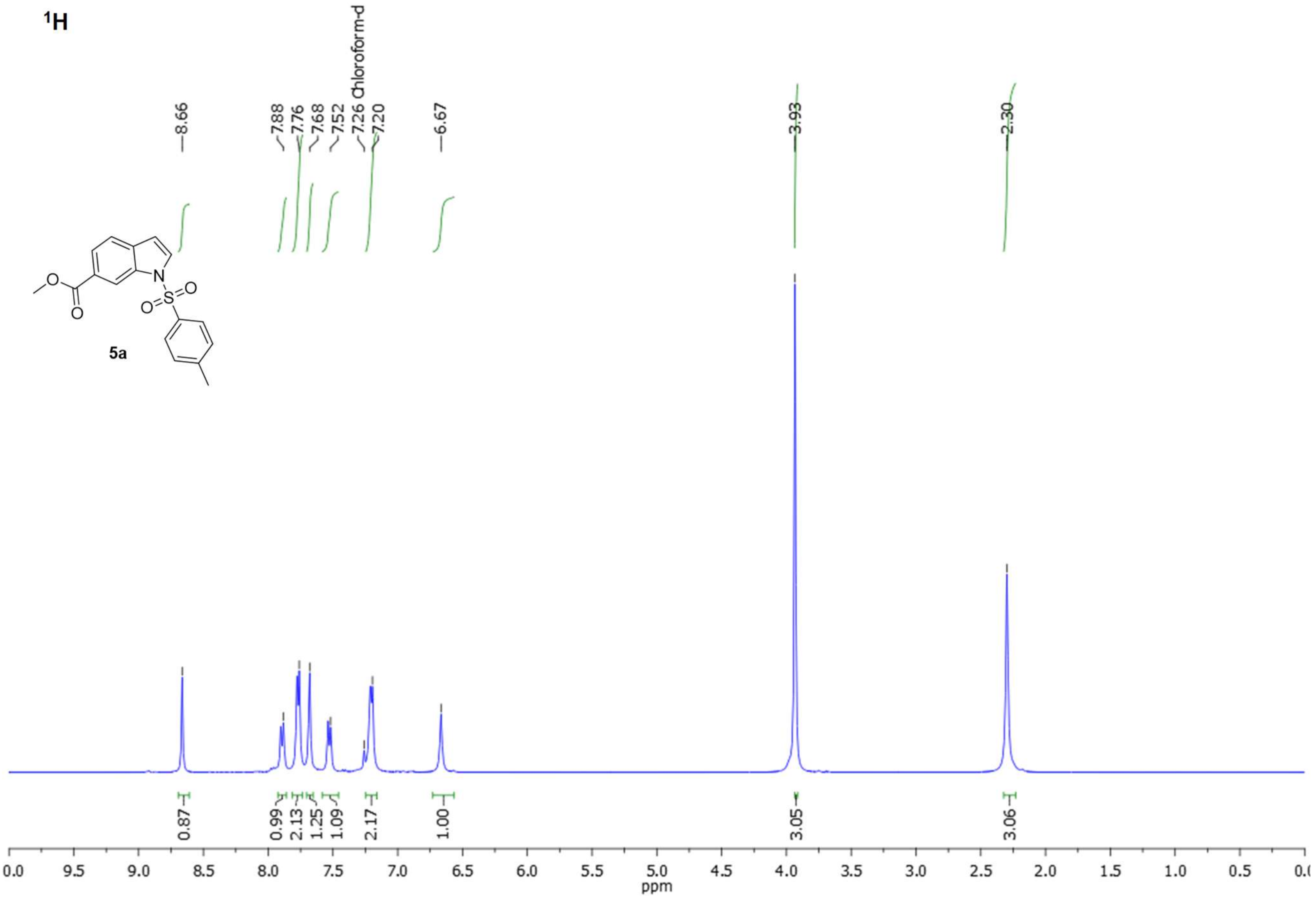
1H
8.66
7.88
7.76
7.68
7.52
7.26 Chloroform-d
7.20
6.67
3.93
2.30
5a
0.87
0.99
2.13
1.25
1.09
2.17
1.00
3.05
3.06
ppm

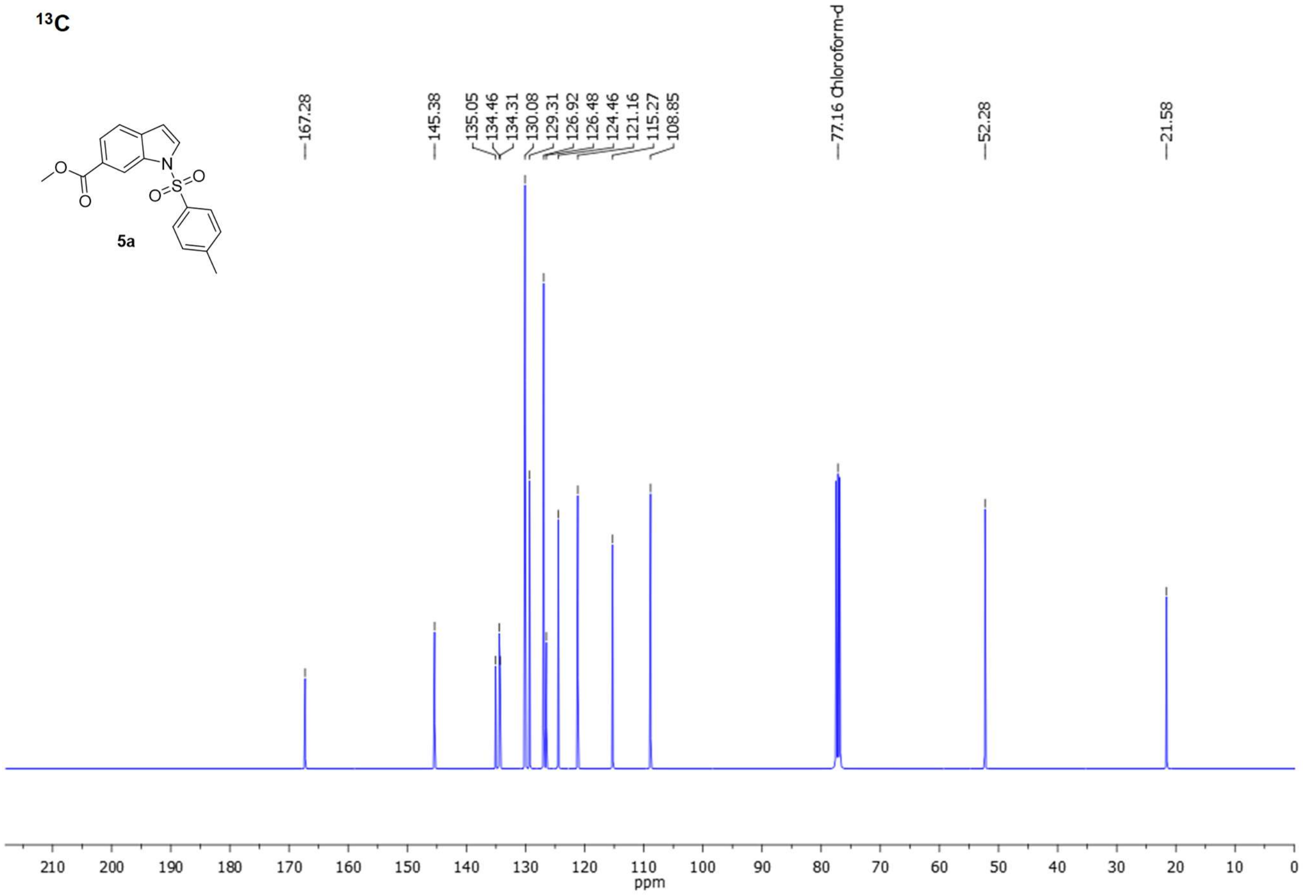
13C
167.28
145.38
135.05
134.46
134.31
130.08
129.31
126.92
126.48
124.46
121.16
115.27
108.85
77.16 Chloroform-d
52.28
21.58
5a
ppm

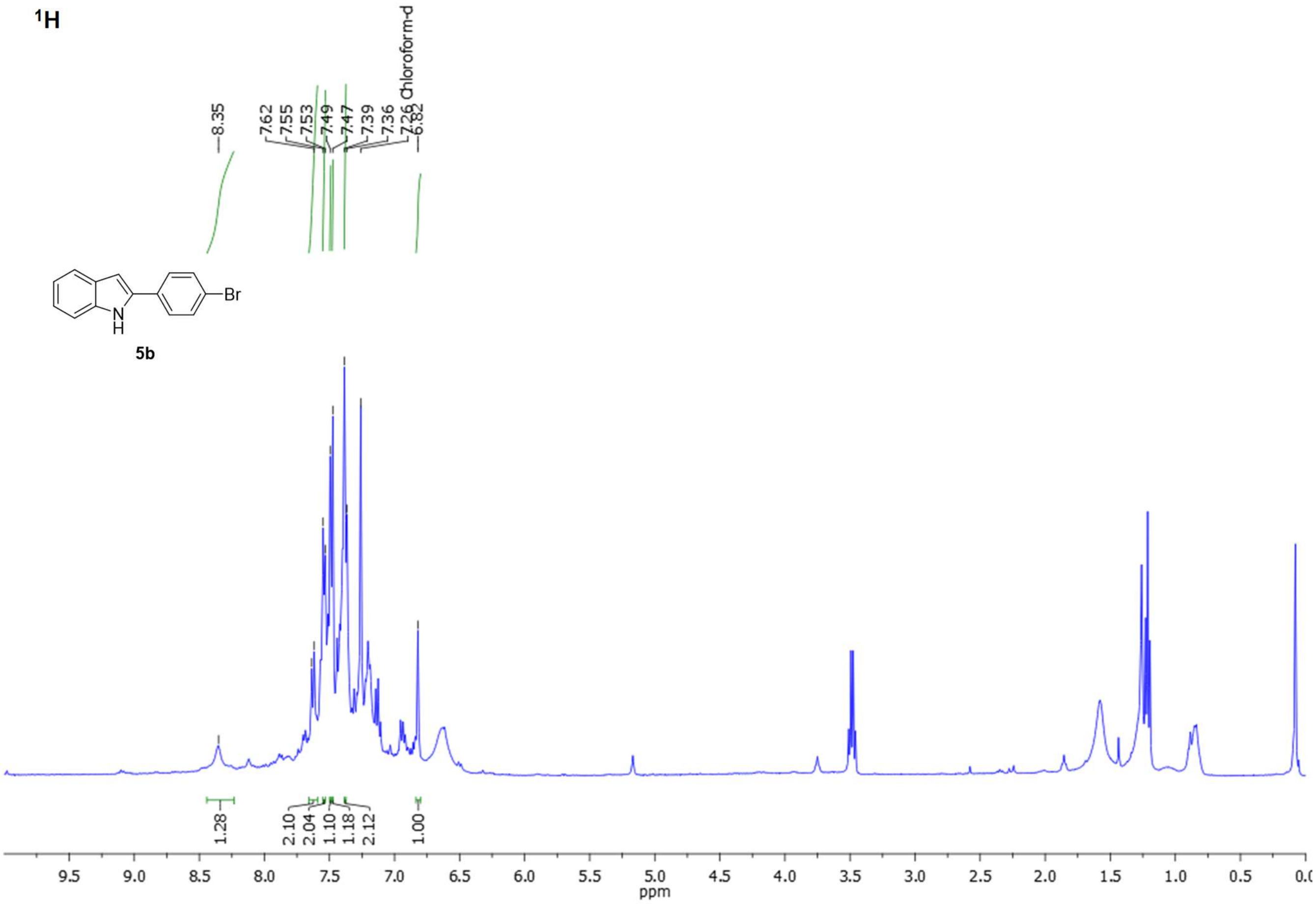
1H
5b
8.35
7.62
7.55
7.53
7.49
7.47
7.39
7.36
7.26 Chloroform-d
6.82
1.28
2.10
2.04
1.10
1.18
2.12
1.00
ppm

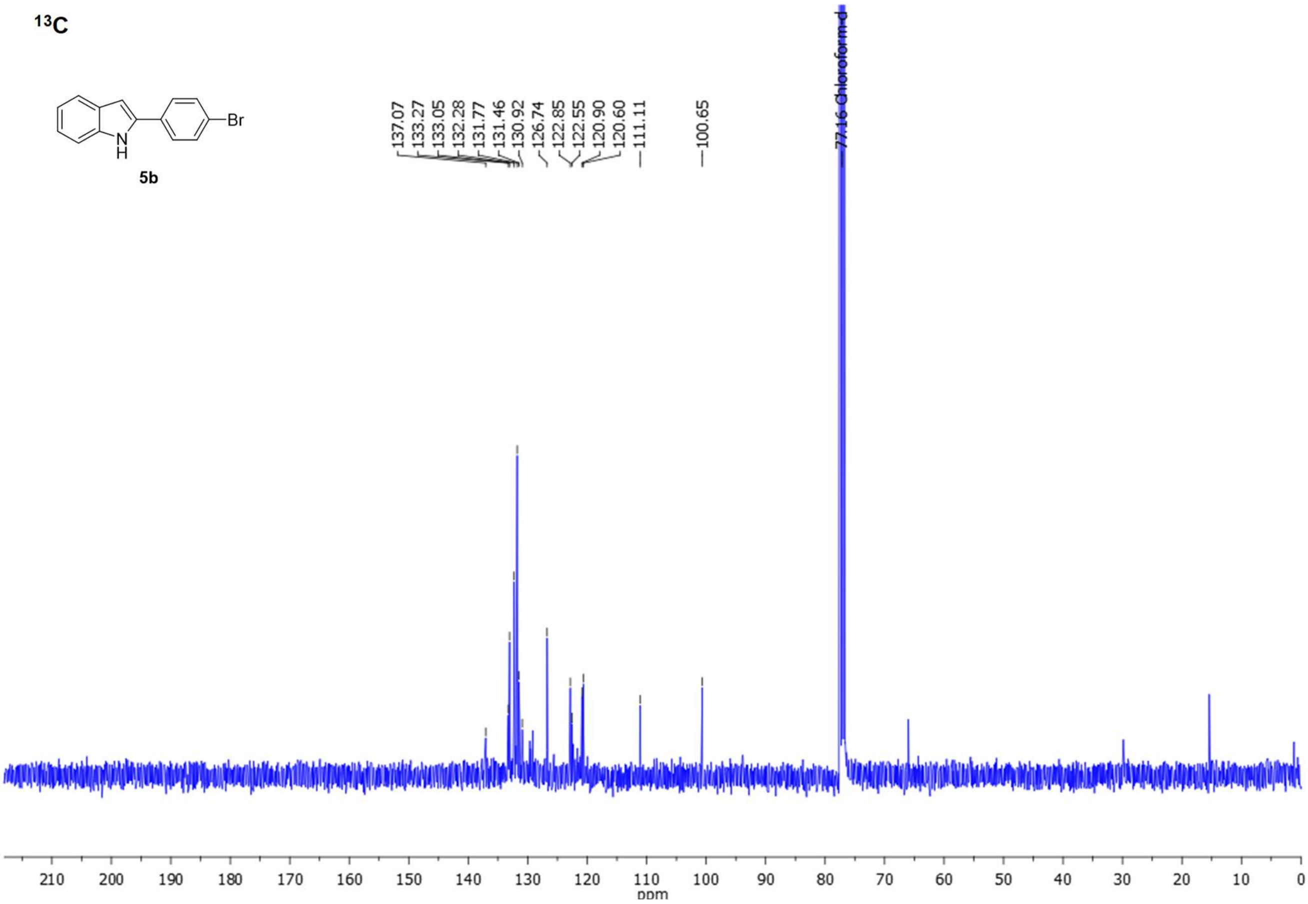
13C
5b
137.07
133.27
133.05
132.28
131.77
131.46
130.92
126.74
122.85
122.55
120.90
120.60
111.11
100.65
77.16 Chloroform-d
ppm

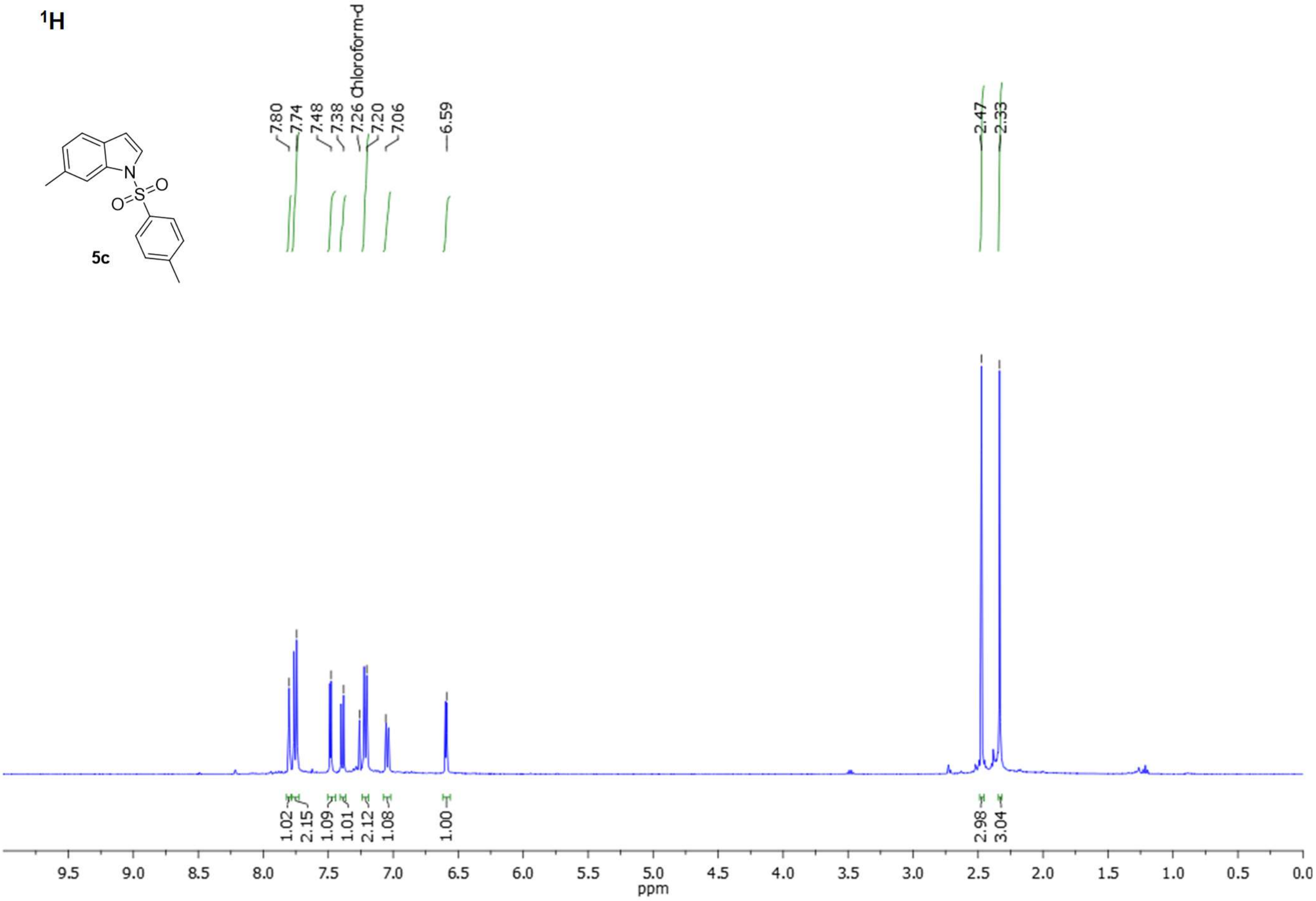
1H
5c
7.80
7.74
7.48
7.38
7.26 Chloroform-d
7.20
7.06
6.59
2.47
2.33
1.02
2.15
1.09
1.01
2.12
1.08
1.00
2.98
3.04
9.5 9.0 8.5 8.0 7.5 7.0 6.5 6.0 5.5 5.0 4.5 4.0 3.5 3.0 2.5 2.0 1.5 1.0 0.5 0.0
ppm

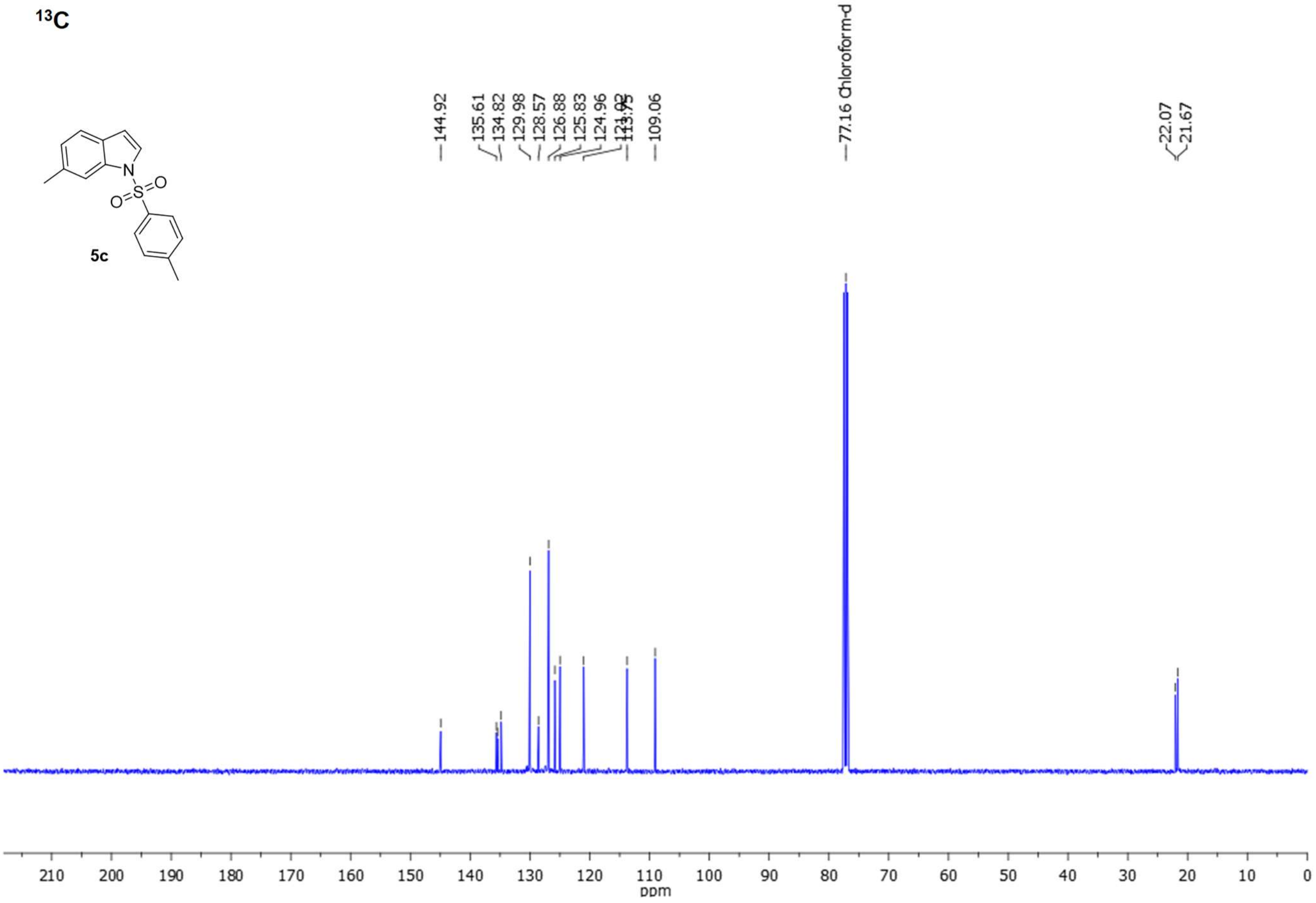
13C
5c
144.92
135.61
134.82
129.98
128.57
126.88
125.83
124.96
121.92
113.75
109.06
77.16 Chloroform-d
22.07
21.67
210 200 190 180 170 160 150 140 130 120 110 100 90 80 70 60 50 40 30 20 10 0
ppm

Author Contributions

D. P dlC-P. conducted and interpreted the experiments; M.M. designed and interpreted the experiments, and supervised the work; A.L.–P. conceived the idea and supervised the work. All authors have participated in the writing of the manuscript and have approved the final version.
